# Design, Synthesis, and Anticancer Evaluation of Novel Tetracaine Hydrazide-Hydrazones

**DOI:** 10.1021/acsomega.2c07192

**Published:** 2023-02-28

**Authors:** M. İhsan Han, Nalan İmamoğlu

**Affiliations:** † Department of Pharmaceutical Chemistry, Faculty of Pharmacy, Erciyes University, Kayseri 38039, Turkey; ‡ Department of Basic Sciences, Faculty of Pharmacy, Erciyes University, Kayseri 38039, Turkey

## Abstract

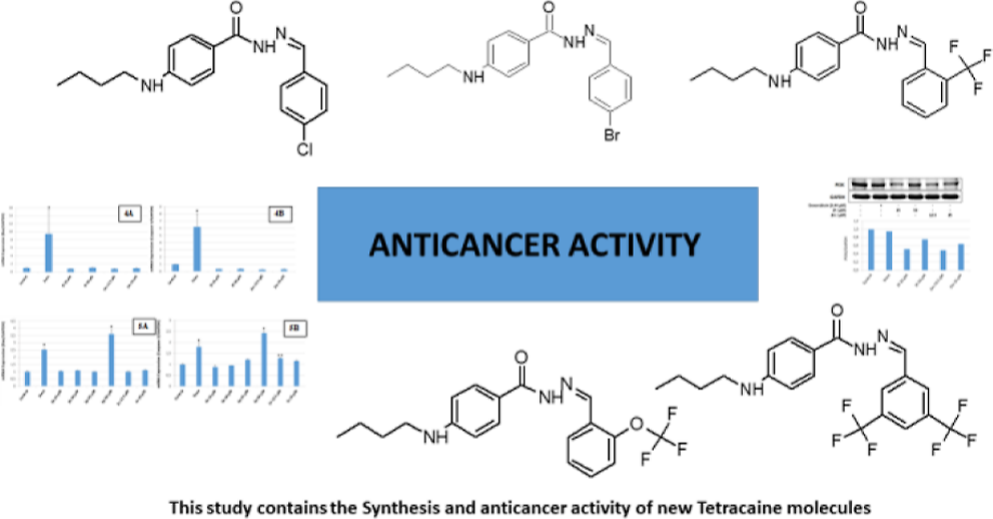

Tetracaine is an ester derivative used as a local anesthetic molecule. In this study, a series of novel Tetracaine derivatives bearing hydrazide-hydrazone moiety were designed, synthesized, and evaluated for anticancer activity. The structures of these compounds were characterized by spectral (^1^H NMR,^13^C NMR, FT-IR, and HRMS analyses) methods. All synthesized compounds were screened for anticancer activity against two different human cancer cell lines (Colo-205 and HepG2). Among the synthesized molecules, compounds **2f** and **2m** showed the most potent anticancer activity against the Colo-205 cell line (IC_50_ = 50.0 and 20.5 μM, respectively). Compounds **2k**, **2p**, and **2s** demonstrated the best anticancer activity against the HepG2 cell line (IC_50_ = 30.5, 35.9, and 20.8 μM, respectively). mRNA transcription levels of Bax and caspase-3 genes were determined by real-time polymerase chain reaction (qRT-PCR) analysis of both Colo-205 and HepG2 cell lines. Doxorubicin was used as a positive sensitivity reference standard. qRT-PCR analysis showed that there was a time-dependent rise in the expression levels of Bax and Caspase 3 on apoptosis. Inhibition of apoptotic proteins PI3K, Akt, PTEN, pPTEN, FoXO1, FoXO3a, TXNIP, and p27 was investigated in Colo-205 and HepG2 cells treated with compounds **2f, 2m, 2k, 2p**, and **2s** by using Western blotting.

## 1 Introduction

Cancer is a disease that includes abnormal cell growth with the potential to invade or metastasize to other parts of the body. Many studies aimed at treating cancer have been carried out for many years.^1,2^ Hepatocellular carcinoma (HCC) is adults’ most extensive type of primary liver cancer.^3^ Colon cancer cells that have evolved from normal epithelial cells to adenocarcinoma.^4^ Local anesthetics have an impressive history of safety and impact in dental and medical use.^5^ The structure of local anesthetics consists of three components: a lipophilic aromatic group, a hydrophilic amino group, and an intermediary link.^6^ Tetracaine is a local anesthetic molecule that is especially used to anesthetize the nose, eyes, or throat. To decrease pain, tetracaine may be implemented on the skin before beginning an intravenous procedure.^7^ The mechanism of tetracaine is explained to change the function of calcium release channels (ryanodine receptors). Tetracaine controls the leave of calcium from intracellular stores.^8^ Diverse research has been carried on in the last years with Tetracaine and its derivatives. Various pharmacological activities were investigated by many researchers such as antimicrobial^9,10^ and anticancer.^11,12^ Also, hydrazide-hydrazone derivatives have been reported to have diverse pharmacological activities, especially anticancer activity.^13−27^ Han *et al.* synthesized novel hydrazide-hydrazone molecules and controlled the anticancer activity on the HepG2 cell line.^22^ Anticancer activity studies of hydrazide-hydrazone molecules have been observed in many studies, and it has been determined that hydrazide-hydrazone molecules show activity against Colo-205 and HegG2 cell lines.^28−33^

**Figure 1:**
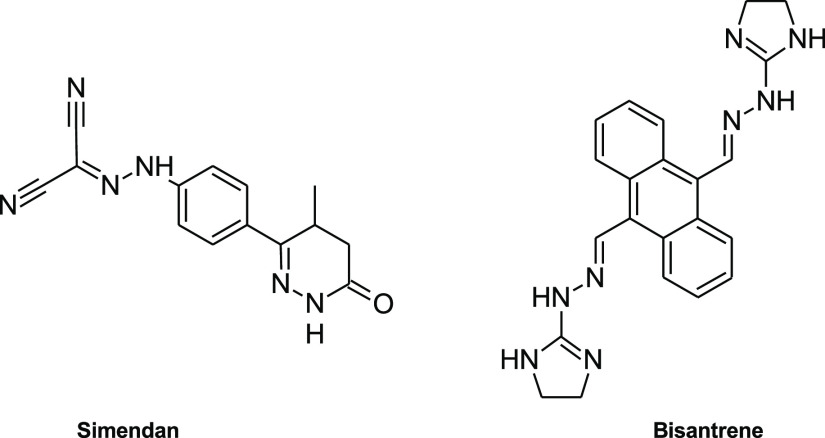
Chemical structures of hydrazone linkage-based drugs.

**Figure 2:**
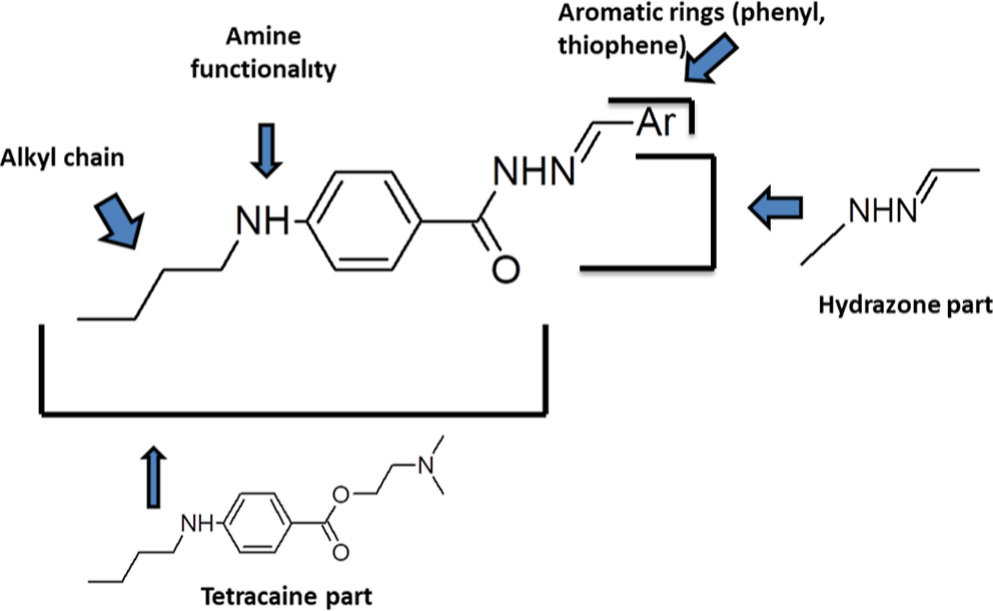
Design strategy of the synthesized compounds **2a–t**.

An azomethine (−NHN=CH−) proton becomes on hydrazide-hydrazones after the synthesis. This function forms an important class of compounds for new drug design.^34−38^ The activity of hydrazide-hydrazones is known to be related to the active pharmacophoric group. Many members of this class are simendan and bisantrene which are used for various diseases treatments (Figure 1). According to the general synthesis method, hydrazide-hydrazones are synthesized by reacting hydrazides with aldehydes. In this study, hydrazide-hydrazone molecules were designed from the hydrazides of Tetracaine. Molecular hybridization is one of the wide ways to design and develop new molecules. This process includes combining two or more pharmacophore groups in a chemical structure. It could be directly or by adding intermediate groups. In this way, new active molecules without side effects can be obtained by using the main structure of a molecule whose biological effect is known (Figure 2).

These investigations led us to synthesize tetracaine hydrazide–hydrazone derivatives. All compounds were characterized by fourier-transform infrared (FT-IR), nuclear magnetic resonance (^1^H NMR, ^13^C NMR), high-resolution mass spectrometry (HRMS), and spectroscopic methods. The anticancer activities of the synthesized compounds were controlled against Colo-205 (colon cancer) and HepG2 (HCC) human cancer cell lines.

## 2 Results and Discussion

### 2.1 Chemistry

**Scheme 1:**
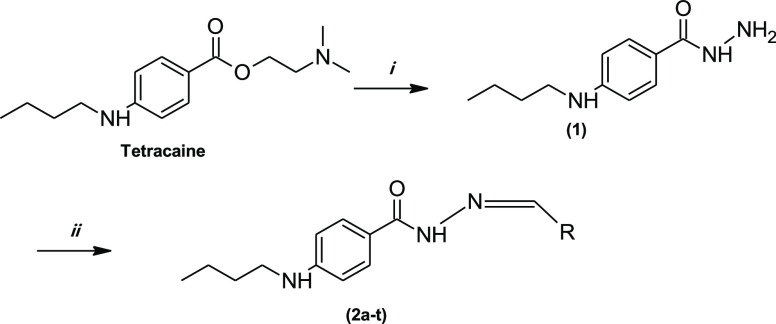
Synthesis Method of Tetracaine Derivatives: (i) NH_2_NH_2_·H_2_O/C_2_H_5_OH and (ii) C_2_H_5_OH/Ar-CHO

Tetracaine (2-(dimethylamino)ethyl 4-(butylamine)benzoate) was chosen as an initial molecule for the synthesis of new hydrazide–hydrazone compounds. In this study, we synthesized 20 novel molecules, as shown in Scheme 1. 4-(Butylamino)benzohydrazide (**1**) was prepared by heating hydrazine hydrate at (80%) and the Tetracaine in the ethanolic medium. Final hydrazide–hydrazone molecules (**2a–t**) were synthesized with compound **1** and selected many aromatic aldehydes in a few drops of glacial acetic acid in an ethanolic medium (Scheme 1). Compounds **2a–t** are original molecules. All synthesized molecules were characterized by their ^1^H NMR, ^13^C NMR, FT-IR, and HR-MS spectroscopic data.^20−22^

The results obtained from the spectral data were all appropriate to the suggested structures. FT-IR analysis of new hydrazide-hydrazones showed that hydrazide-hydrazone C=O stretching bands were recorded from 1643 to 1624 cm^–1^. In all compounds, N–H bands were recorded at 3305–3221 cm^–1^, and C=N bands were recorded at 1612–1593 cm^–1^, altogether proving the special hydrazide-hydrazone C=N stretching bands. In the ^1^H NMR spectra of all molecules (**2a–t**), the signal symbolizing most of the azomethine=CH protons found at 7.83–8.33 ppm singlets and −NH amide protons (−CONHN=CH−) was recorded singlets at 11.52–11.91 ppm. Secondary amine protons were recorded at 6.32–6.40 ppm. The other protons appeared at the expected chemical changes and integral values. The formation of hydrazide-hydrazones was also confirmed with ^13^C NMR studies. The signals belonging to the −C=O group at compounds **2a–t** have been detected at 172.92–161.80 ppm. Other important −C=N group signals were detected at 149.43–139.08 ppm. HR-MS results showed the molecular weights and empirical formulae of the compounds, with a <5 mmu tendency between the calculated and experimental *m*/*z* values of the molecules. All compounds gave the [M+1] peak at their molecular weight because of catching a hydrogen atom from a medium according to the used method with ESI method. Tetracaine hydrazide–hydrazones gave relatively steady molecular ion peaks in the appropriate mass spectra.

### 2.2 Biological Evaluation

#### 2.2.1 *In Vitro* Anticancer Activity

The anticancer activity of the synthesized new Tetracaine hydrazide-hydrazone molecules was evaluated on human HCC cell line HepG2 and colon carcinoma cell line Colo-205. The cytotoxic activity studies were implemented by 2H-tetrazolium and 2-(4,5-dimethyl-2-thiazolyl)-3,5-diphenyl-bromide (MTT) assay, and subsequently, IC_50_ values were determined. Doxorubicin was used as a positive control.

**Figure 3:**
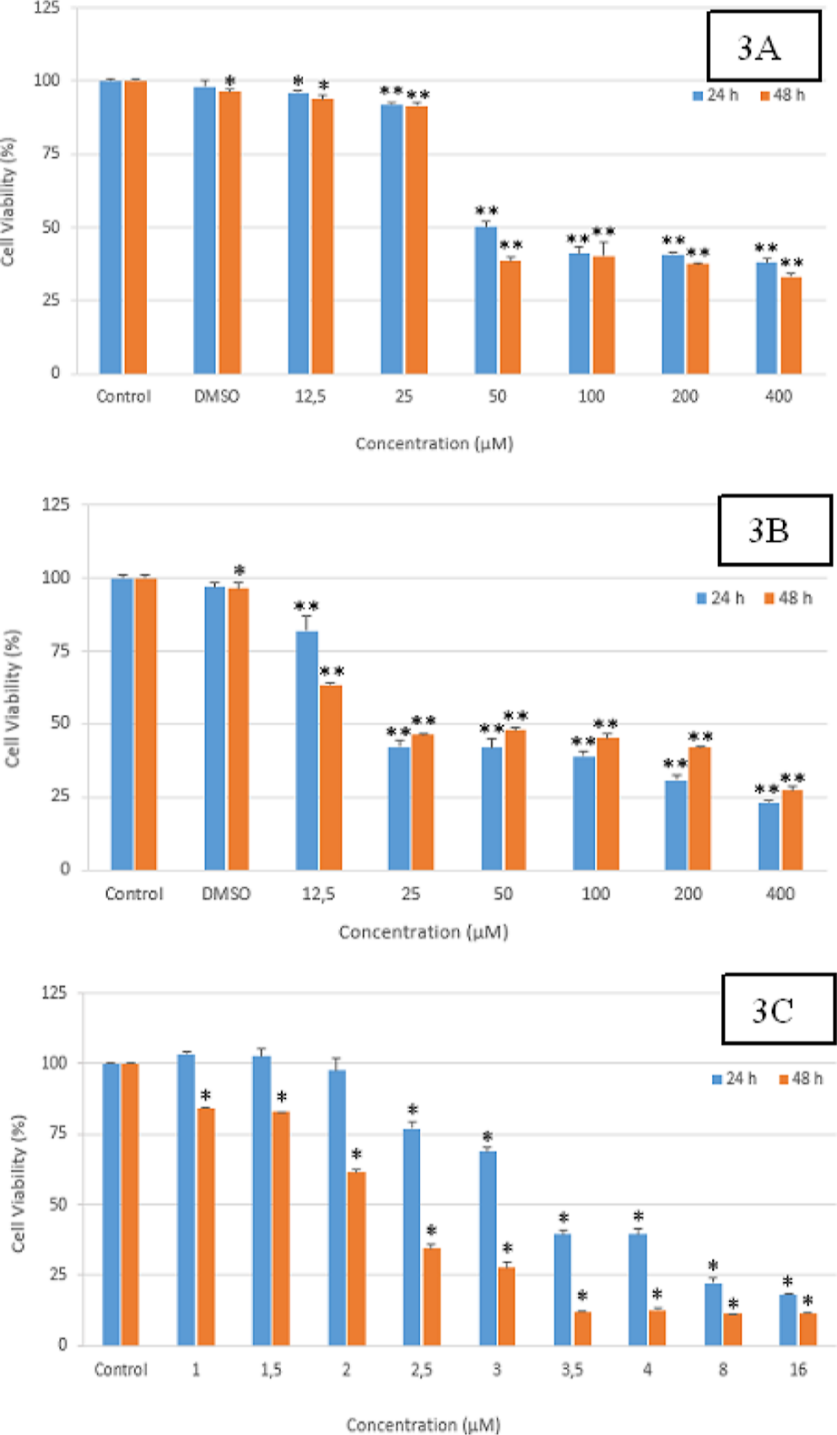
(A–C) Cell viability graphics of compounds **2f**, **2m**, and **DOXO** on the Colo-205 cell line. (3A): Compound 2f *p < 0.05, **p < 0.001, (*n* = 3); (3B): compound **2m**; *p < 0.05, **p < 0.001, (*n* = 3); 3C: Doxo; *p < 0.001, (*n* = 3).

**Figure 4:**
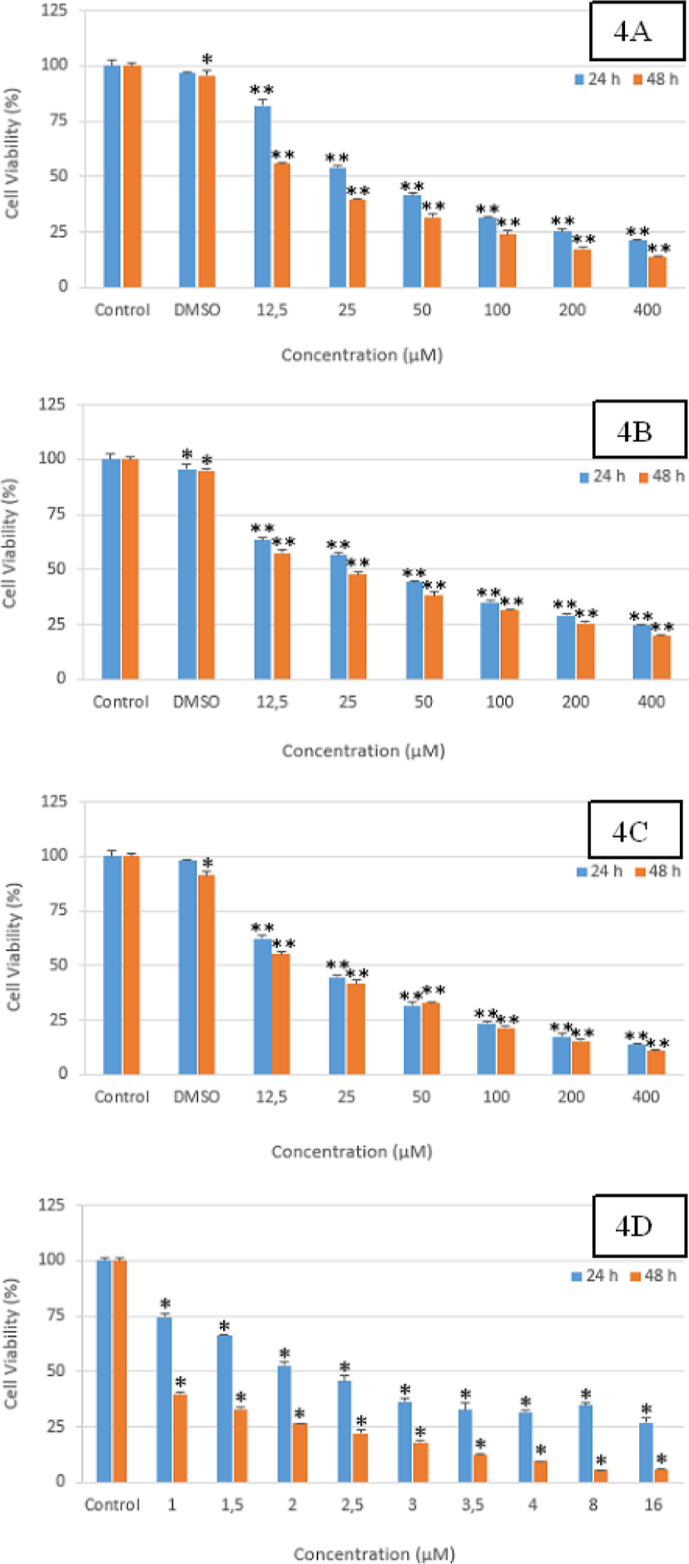
(A–D) Cell viability graphics of compounds **2k**, **2p**, **2s,** and DOXO on the HepG2 cell line. (4A): Compound **2k** *p < 0.05, **p < 0.001, (*n* = 3); (4B): compound **2p**; *p < 0.05, **p < 0.001, (*n* = 3); (4C): compound **2s**; *p < 0.05, **p < 0.001, (*n* = 3); (4D): Doxo; *p < 0.001, (*n* = 3).

Many compounds (**2f, 2m, 2k, 2p, and 2s**) showed antitumor activity with IC_50_ values in the range of 20.5–50.0 μM against the Colo-205 and HepG2 cancer cell lines. The compounds **2f** and **2m** showed anticancer activity against cancer cell line Colo-205 with IC_50_ values 50.0 and 20.5 μM, respectively for 24 h. For 48 h, compounds **2f** and **2m** showed anticancer activity with IC_50_ values 46.0 and 17.0 μM, respectively. Compounds **2k**, **2p**, and **2s** demonstrated anticancer activity against cancer cell line HepG2 with IC_50_ values of 30.5, 35.9, and 20.8 μM, respectively for 24 h and 14.8, 20.6, and 14.4 μM for 48 h. Because of the aromatic ring substituent, all Tetracaine hydrazide–hydrazones are dissimilar from each other. Compounds showing anticancer activity for both cell lines are different from each other. In the Colo-205 cell line, the presence of 4-chlorophenyl and 2-trifluoromethylphenyl substituents was required for cytotoxic activity. In the HepG2 cell line, 4-bromophenyl, 2-trifluoromethoxyphenyl, and 3,5-bistrifluoromethylphenyl substituents have shown the best anticancer activity. We controlled our starting material Tetracaine’s IC_50_ values for both the cell lines and compared Tetracaine and the novel hydrazide-hydrazone structure. Tetracaine showed anticancer activity against the Colo-205 and HepG2 cancer cell lines with IC_50_ values 129.2 and 117.4 μM, respectively, for 24 h. This result suggests that hydrazide–hydrazone moiety may show an efficient role in defining anticancer activity. The outcome of the synthesized compounds was noticed in terms of IC_50_ values in Table 1. Graphics of MTT studies are given in Figures 3A–C and 4A–D.

**Table 1:** IC_50_ Results (μM) of the Novel Tetracaine Compounds (**2a–t**)

Compounds	–R	Colo-205	HepG2
2a	–C_5_H_4_N (2)	130.4	71.5
2b	–C_5_H_4_N (3)	>400	>400
2c	–C_5_H_4_N (4)	>400	>400
2d	–C_6_H_4_–Cl (2)	>400	65.7
2e	–C_6_H_4_–Cl (3)	>400	>400
2f	–C_6_H_4_–Cl (4)	**50.0**	229.5
2g	–C_6_H_4_–Cl, F (2,6)	>400	361.9
2h	–C_6_H_4_–F (2)	>400	329.5
2i	–C_6_H_4_–F (3)	>400	196.5
2j	–C_6_H_4_–F (4)	>400	375
2k	–C_6_H_4_–Br (4)	>400	**30.5**
2l	–C_6_H_4_–CN (4)	>400	>400
2m	–C_6_H_4_–CF_3_ (2)	**20.5**	62.9
2n	–C_6_H_4_–CF_3_ (3)	>400	192.1
2o	–C_6_H_4_–CF_3_ (4)	–>400	>400
2p	–C_6_H_4_–OCF_3_ (2)	185.7	**35.9**
2q	–C_6_H_4_–OCF_3_ (3)	>400	>400
2r	–C_6_H_4_–OCF_3_ (4)	>400	>400
2s	–C_6_H_4_–CF_3_ (3,5)	299.4	**20.8**
2t	–C_4_H_3_S–phe (4)	>400	60.1
Tetracaine		129.2	117.3
Doxorubicin		**3.4**	**2.1**

#### 2.2.2 RT-PCR Studies

**Figure 5:**
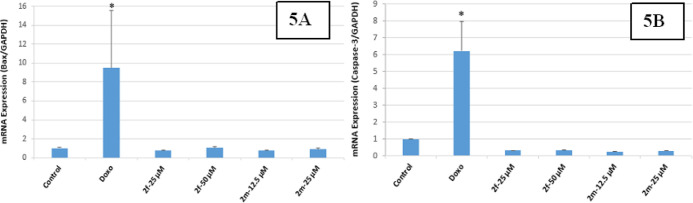
(A,B) (5A) Expression rate of compounds **2f** and **2m** on Bax/GAPDH [*p < 0.01, (*n* = 2)], (5B) effects of compounds **2f** and **2m** on caspase-3 activity on Colo-205 cells [*p = 0.001, (*n* = 2)].

**Figure 6:**
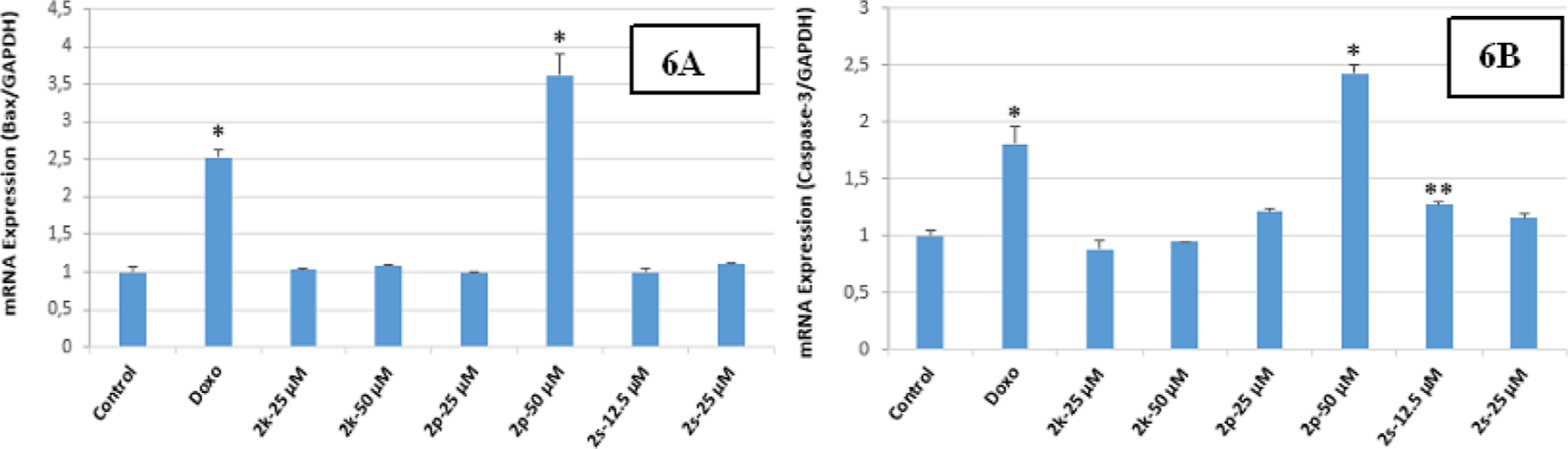
(A,B) (6A) Expression rate of compounds **2k**, **2p**, and **2s** on Bax/GAPDH [*p < 0.01, (*n* = 2)], (6B) effects of compounds **2k**, **2p**, and **2s** on caspase-3 activity on HepG2 cells [*p < 0.001, **p < 0.05 (*n* = 2)].

In this study, the impacts of compounds **2k, 2p, 2s, 2f**, and **2m** on the mitochondrial membrane potential were researched. Bax as a mitochondrial apoptotic marker and caspase-3 activation as a marker to the point of convergence of the intrinsic and extrinsic apoptotic ways were controlled in the HepG2 and Colo-205 cell lines. As a result of MTT studies with the compounds we synthesized, the effects of **2f** and **2m** compounds and **2k, 2p,** and **2s** compounds, which show mutual cytotoxic activity in Colo-205 and HepG2 cells analyzed with the RT-qPCR method on the mRNA transcription levels of pro-apoptotic Bax and Caspase-3 genes, play a role in apoptosis. The transcription levels of the genes were normalized with the transcription level of the GAPDH control gene. The results obtained were compared with the results obtained from Doxorubicin as a positive control in the study. HepG2 and Colo-205 cells were exposed to compounds **2k, 2p, 2s** (HepG2), **2f, and 2m** (Colo-205) for their cytotoxic dose. Messenger RNA (mRNA) expression analyses of the Bax and Caspase-3 were performed for evaluating apoptosis. Real-time polymerase chain reaction (rt-PCR) analysis showed that there was a time-dependent increase in the expression levels of Bax and Caspase-3 and compounds **2p** and **2s**-treated HepG2 cells compared with the control and Doxorubicin. None of the molecules showing cytotoxic activity demonstrated any change in Bax and Caspase-3 levels in the rt-PCR study for Colo-205 cells. These data showed that 24 h treatment with compounds **2p** and **2s** directly up-regulated Bax expression and induced caspase-3-dependent apoptosis in the HepG2 cell line. All these results showed that treatment with **2p** and **2s** caused a significant increase in apoptotic markers (Figures 5A,B and 6A,B).

#### 2.2.3 Western Blot Studies

In our study, PI3K, Akt, PTEN, pPTEN, FoXO1, FoXO3a, TXNIP, and p27 protein expressions were analyzed in Colo-205 and HepG2 cells using Western blotting for controlling the induction of apoptotic proteins. Colo-205 and HepG2 cells were treated with compounds **2f and 2m** (Colo-205) and **2k, 2p, and 2s** (HepG2) according to IC_50_ concentration for 24 h and compared with untreated control cells. Doxorubicin was utilized as a positive control.

The PI3K/Akt signaling pathway is an important signaling pathway that plays a role in cell proliferation, cell cycle regulation, and apoptosis. It is stated that apoptosis and growth inhibitory activities are suppressed with the activation of the Akt pathway, and tumor formation is supported.

In this study, the effects of compounds **2f** and **2m** on the PI3K/Akt signaling pathway that regulates cell survival, proliferation, and differentiation in Colo-205 cells and on FoxO signaling pathways are also important in cell proliferation and metabolism. All compounds were investigated for the first time at the molecular level since they are original molecules.

**Figure 7:**
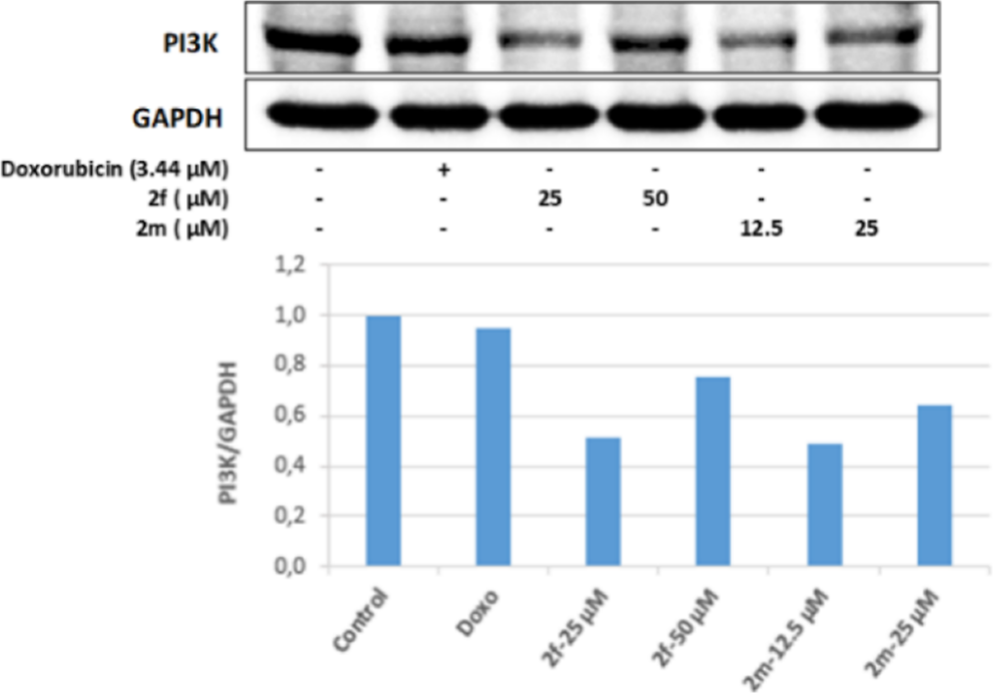
Western blot results of Colo-205 cells treated with IC_50_ concentrations of the compounds **2f**, **2m**, and doxorubicin for 24 h on PI3K levels.

**Figure 8:**
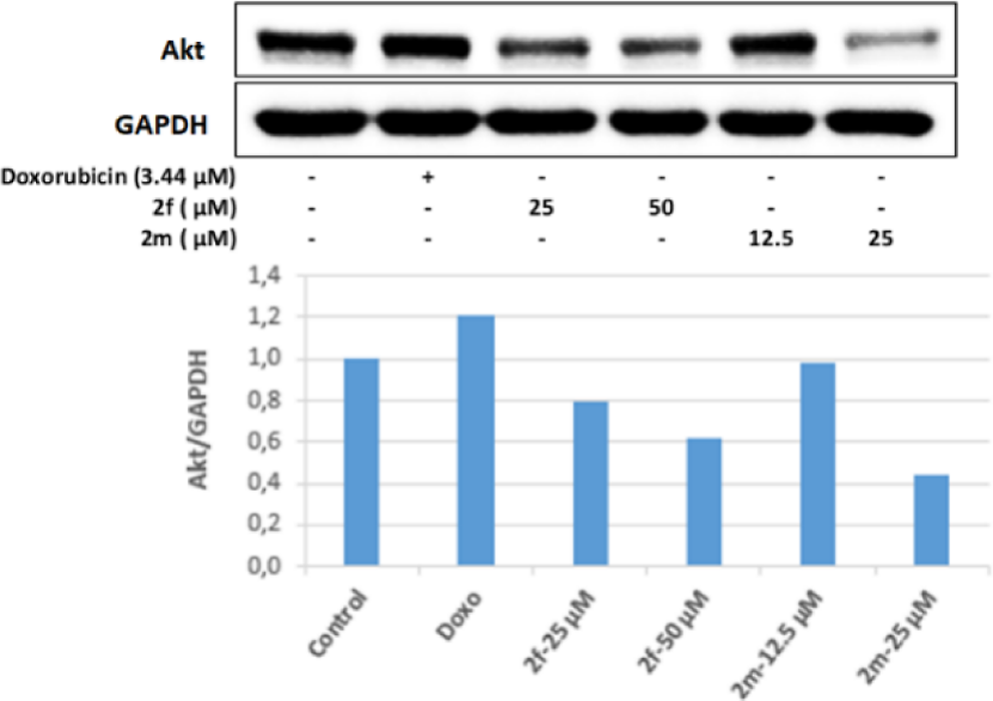
Western blot results of Colo-205 cells treated with IC_50_ concentrations of the compounds **2f**, **2m**, and Doxorubicin for 24 h on Akt levels.

It was observed that PI3K protein levels decreased effectively in cell groups incubated with 25 and 50 μM concentrations of compound **2f**, 12.5, and 25 μM concentrations of **2m**, compared to the control group. In addition, it was observed that the level of PI3K protein in cell groups incubated at both concentrations of **2f** and **2m** decreased in a dose-dependent manner (Figure 7). As a result of the Western blot analysis, while the Akt protein level in the cell group incubated with positive control doxorubicin did not decrease compared to the control group, Akt protein levels in the cell groups incubated with 25 and 50 μM concentrations of compound **2f** and 25 μM concentrations of compound **2m** compared to the control group were found to decrease. It was determined that the most effective reduction in the Akt protein level was in the cell group incubated with a 25 μM concentration of compound **2m** (Figure 8).

**Figure 9:**
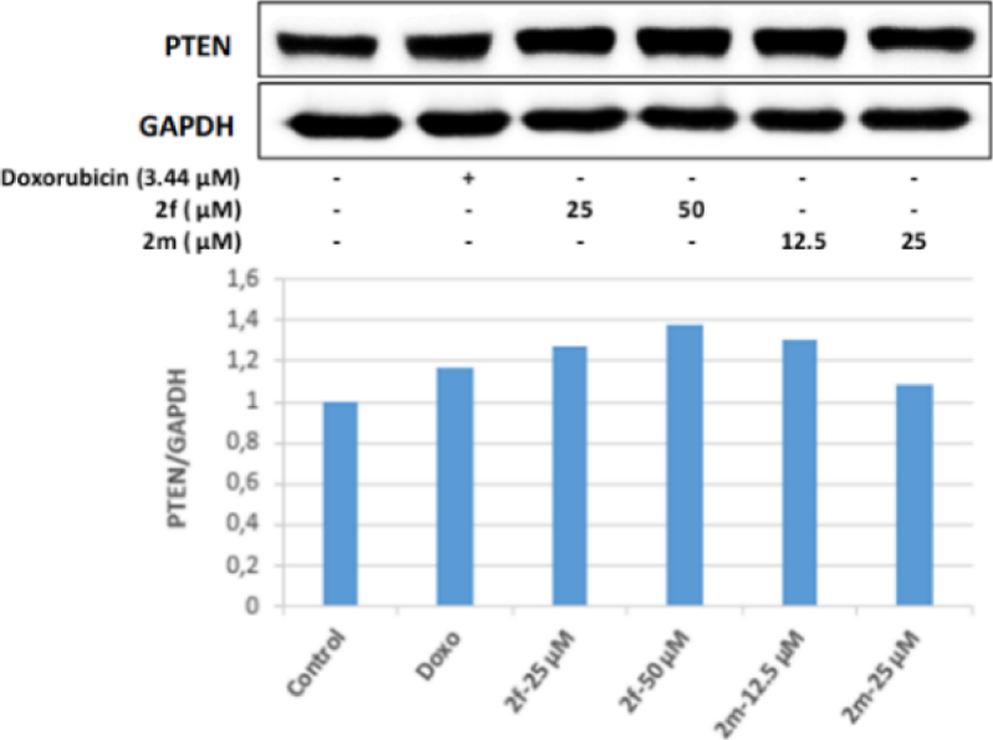
Western blot results of Colo-205 cells treated with IC_50_ concentrations of the compounds **2f**, **2m**, and Doxorubicin for 24 h on PTEN levels.

**Figure 10:**
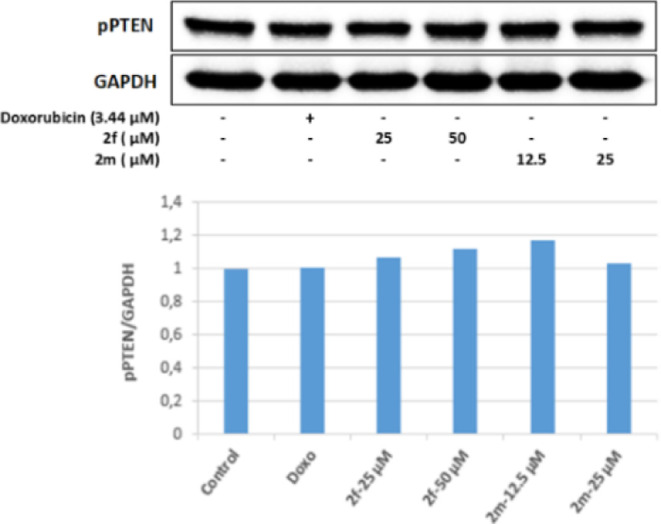
Western blot results of Colo-205 cells treated with IC_50_ concentrations of the compounds **2f**, **2m**, and Doxorubicin for 24 h on pPTEN levels.

**Figure 11:**
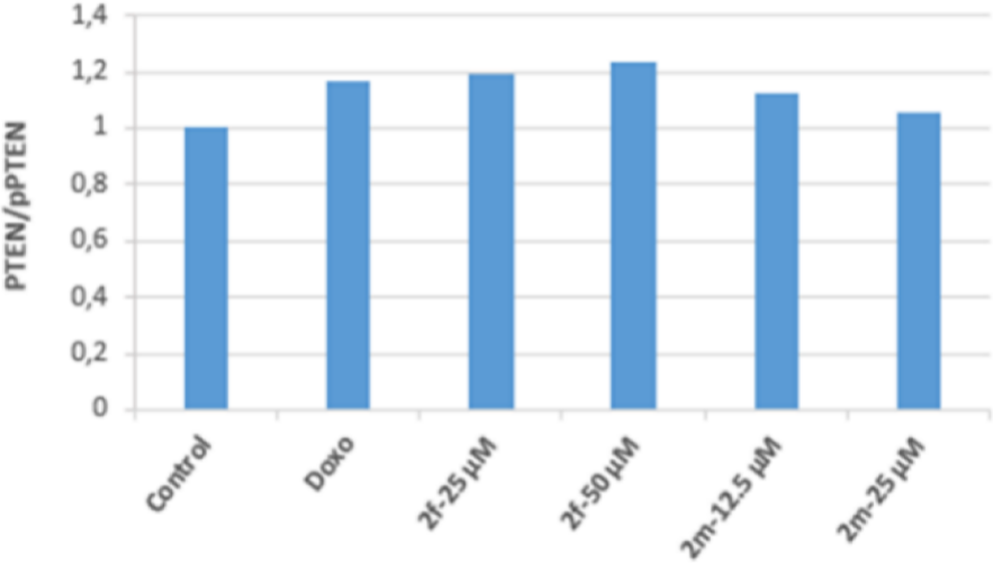
Western blot results of Colo-205 cells treated with IC_50_ concentrations of the compounds **2f**, **2m**, and Doxorubicin for 24 h on the pPTEN/PTEN ratio.

PTEN is a tumor suppressor protein and is also a negative regulator of Akt. It was determined that 25 and 50 μM concentrations of compound **2f** in Colo-205 cells effectively increased the ratio of active PTEN to inactive p-PTEN (pPTEN/PTEN). This result shows that 12.5 and 25 μM concentrations of **2m** may suppress the PI3K/Akt pathway by increasing the pPTEN/PTEN ratio in Colo-205 cells. As a result, the active PTEN ratio increased in Colo-205 cell groups incubated with 25 and 50 μM concentrations of compound **2f**; in parallel with this, the Akt protein level decreased, and PI3K was effectively inhibited in the same groups. This level revealed the antitumor activity. In the cell groups incubated with 50 μM concentrations of compound **2f**, it was observed that the pPTEN/PTEN ratio increased more effectively than the positive control doxorubicin, and this ratio increased approximately 1.2 times compared to the control group (Figures 9–11).

**Figure 12:**
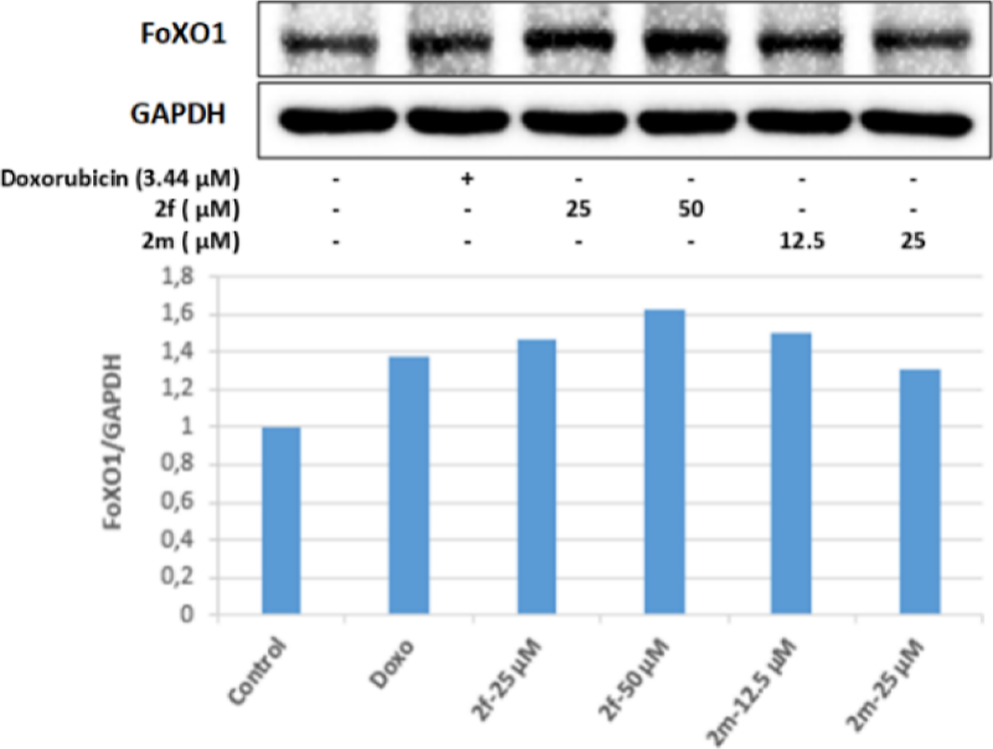
Western blot results of Colo-205 cells treated with IC_50_ concentrations of the compounds **2f**, **2m**, and Doxorubicin for 24 h on FoXO1 levels.

**Figure 13:**
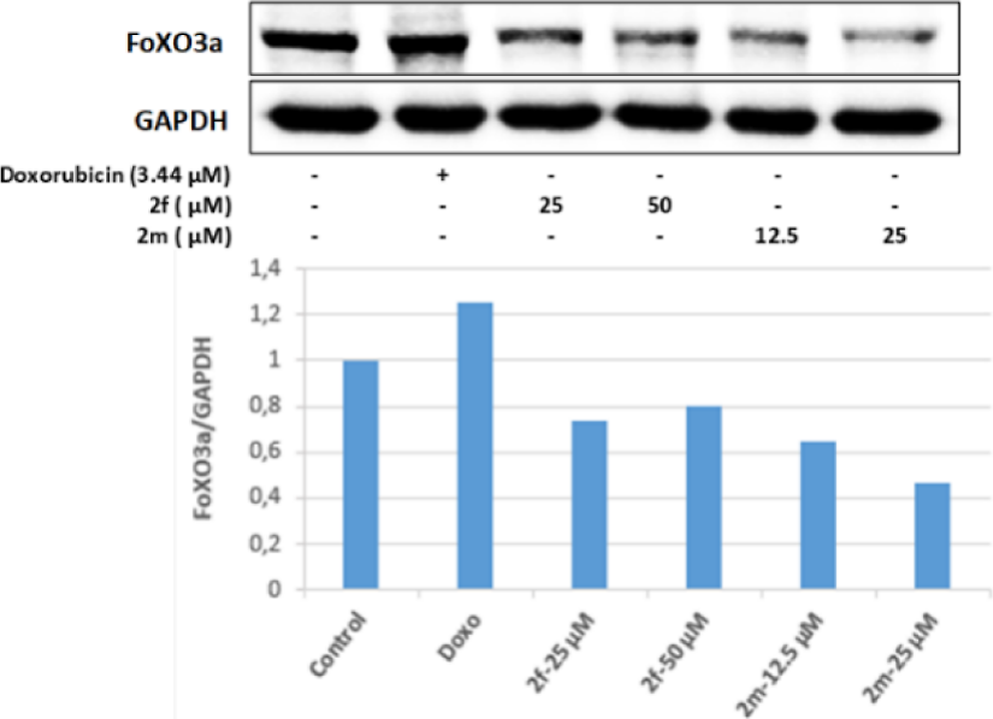
Western blot results of Colo-205 cells treated with IC_50_ concentrations of the compounds **2f**, **2m**, and Doxorubicin for 24 h on FoXO3a levels.

The FoxO pathway is another signaling pathway that is important for cell proliferation and metabolism. FoxO proteins regulate the functioning of the cell by controlling the expression of genes related to apoptosis, autophagy, and cell cycle control. FOXO1 is a transcription factor that regulates the cell cycle and apoptosis and is therefore thought to be involved in cell transformation and tumor development. FOXO3a is also known to play significant roles in apoptosis, autophagy, cell proliferation, DNA damage, and resistance to oxidative stress. Overexpression of FOXO3a has been demonstrated to inhibit proliferation, tumorigenesis, and invasion in cancer cells. In our study, it was observed that compounds **2f** and **2m**, whose antitumoral effects were investigated, increased the FoXO1 protein level in Colo-205 cells more effectively than the positive control doxorubicin. It was determined that the most effective increase in the FoXO1 protein level was in cell groups incubated with 50 μM concentrations of **2f** and 12.5 μM concentrations of **2m**, and the increase in FoXO1 protein expression in these groups was higher than in the control group (Figure 12). On the other hand, it was determined that the controlled substances in Colo-205 cells did not increase the level of FoXO3a protein (Figure 13).

**Figure 14:**
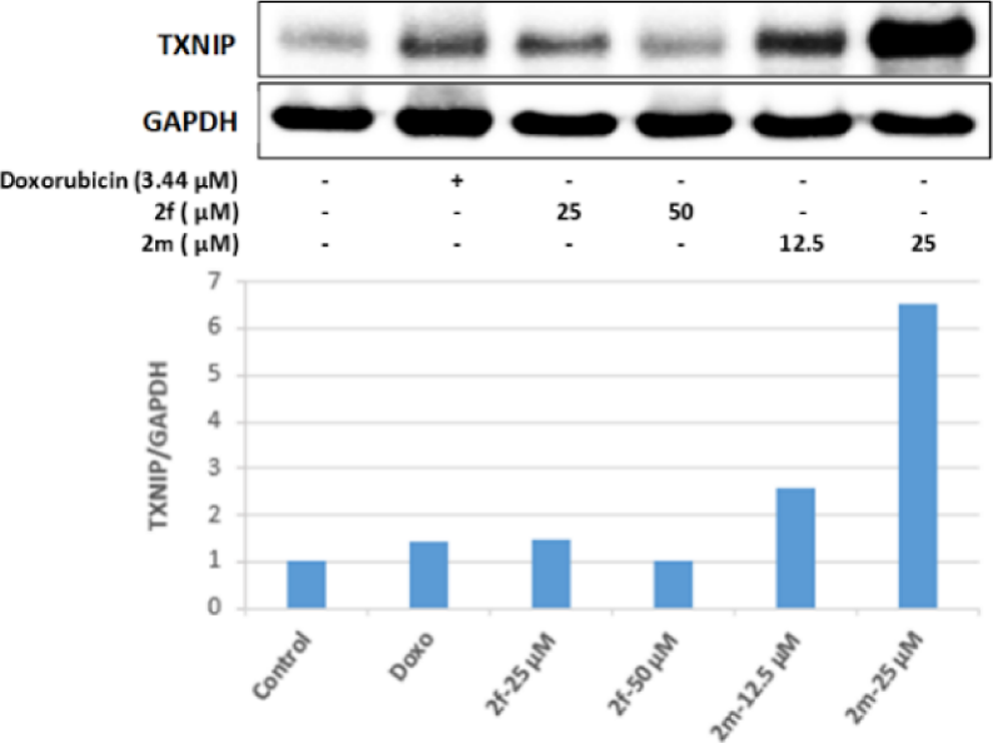
Western blot results of Colo-205 cells treated with IC_50_ concentrations of the compounds **2f**, **2m**, and Doxorubicin for 24 h on TXNIP levels.

TXNIP is one of the target genes of the FoxO pathway and is known to have a tumor suppressor role. It has been noticed that TXNIP expression decreases when the FoxO pathway is suppressed. In our study, 25, 12.5, and 25 μM concentrations of compound **2f**, whose antitumoral effects were investigated, increased TXNIP protein levels by FoXO1 protein levels in Colo-205 cells, and thus TXNIP stimulation occurred as a result of FoxO pathway activation (Figure 14).

**Figure 15:**
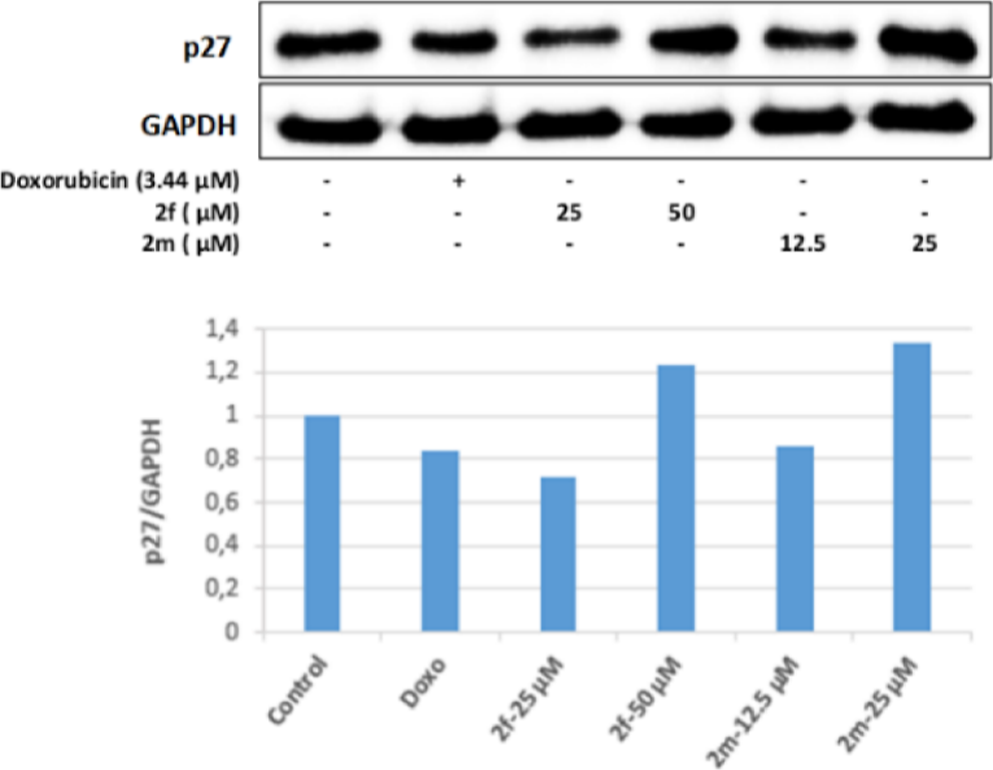
Western blot results of Colo-205 cells treated with IC_50_ concentrations of the compounds **2f**, **2m**, and Doxorubicin for 24 h on p27 levels.

It is known that TXNIP regulates the p27 protein and keeps cells in the G1 phase of the cell cycle. It is known that the CDK inhibitor p27 regulates cell proliferation and apoptosis; however, the level of p27 protein decreases in cancer cells. It was determined that 50 and 25 μM concentrations of compounds **2f** and **2m**, whose antitumoral effects were investigated, effectively increased p27 protein levels in Colo-205 cells in line with FoXO1 and TXNIP protein levels. These results revealed that compounds **2f** and **2m** exert their antiproliferative effects on Colo-205 cells by increasing the level of p27 protein, which keeps the cell cycle in the G1 phase (Figure 15).

In conclusion, it was determined that compounds **2f** and **2m**, whose antitumoral activities were investigated in our study, exert their antitumoral effects on Colo-25 cells through different cellular pathways. Accordingly, it was observed that **2f** and **2m** exert their antiproliferative effect on Colo-205 cells by inhibiting the PI3K/Akt signaling pathway, by inhibiting PI3K and Akt levels, and by reducing the phosphorylation of PTEN. It was determined that **2f** and **2m** exert their antiproliferative effects on Colo-205 cells through the activation of the FoxO pathway by increasing FoXO1, TXNIP, and p27 protein levels.

The effects of compounds **2k**, **2p**, and **2s** on the PI3K/Akt signaling pathway, which regulates cell survival, proliferation, and differentiation, and FoxO signaling pathways, which are also important in cell proliferation, and metabolism, were investigated for the first time at the molecular level in HepG2 cells.

**Figure 16:**
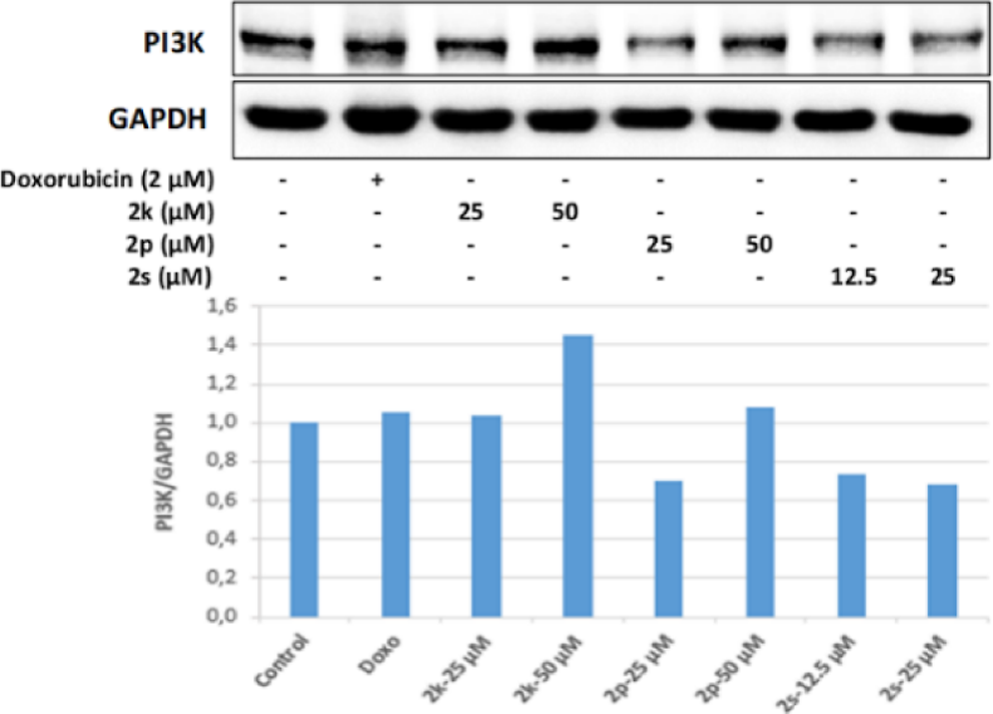
Western blot results of HepG2 cells treated with IC_50_ concentrations of the compounds **2k**, **2p**, **2s**, and Doxorubicin for 24 h on PI3K levels.

**Figure 17:**
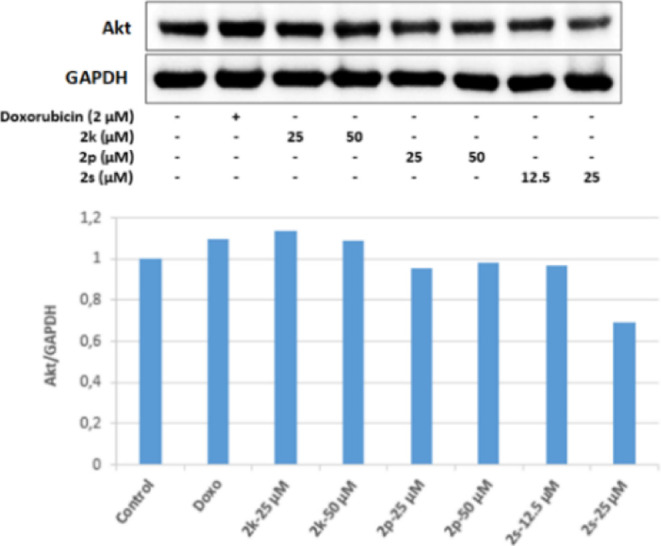
Western blot results of HepG2 cells treated with IC_50_ concentrations of the compounds **2k**, **2p**, **2s**, and Doxorubicin for 24 h on Akt levels.

In our study, 25 μM concentration of compound **2p**, whose antitumoral effects were investigated, and 12.5 and 25 μM concentrations of **2s** were found to inhibit PI3K effectively in HepG2 cells. Similarly, 25 and 50 μM concentrations of **2p** and 12.5 and 25 μM concentrations of **2s** also decreased Akt protein levels in HepG2 cells compared to the control group; particulary, it was observed that 25 μM concentration of **2s** effectively inhibited Akt protein expression. These results are also compatible with quantitative real-time PCR (qRT-PCR) results, and as a result of the qRT-PCR analysis, it was determined that 50 μM concentration of **2p** increased the mRNA expression of pro-apoptotic Bax more effectively than the positive control doxorubicin has done. Similarly, 25 and 50 μM concentrations of **2p** and 12.5 and 25 μM concentrations of **2s** increased the mRNA expression of caspase-3 in HepG2 cells, and 50 μM concentrations of **2p** and 12.5 μM concentrations of **2s** significantly increased caspase-3 mRNA expression compared to the control group (Figures 16 and 17).

**Figure 18:**
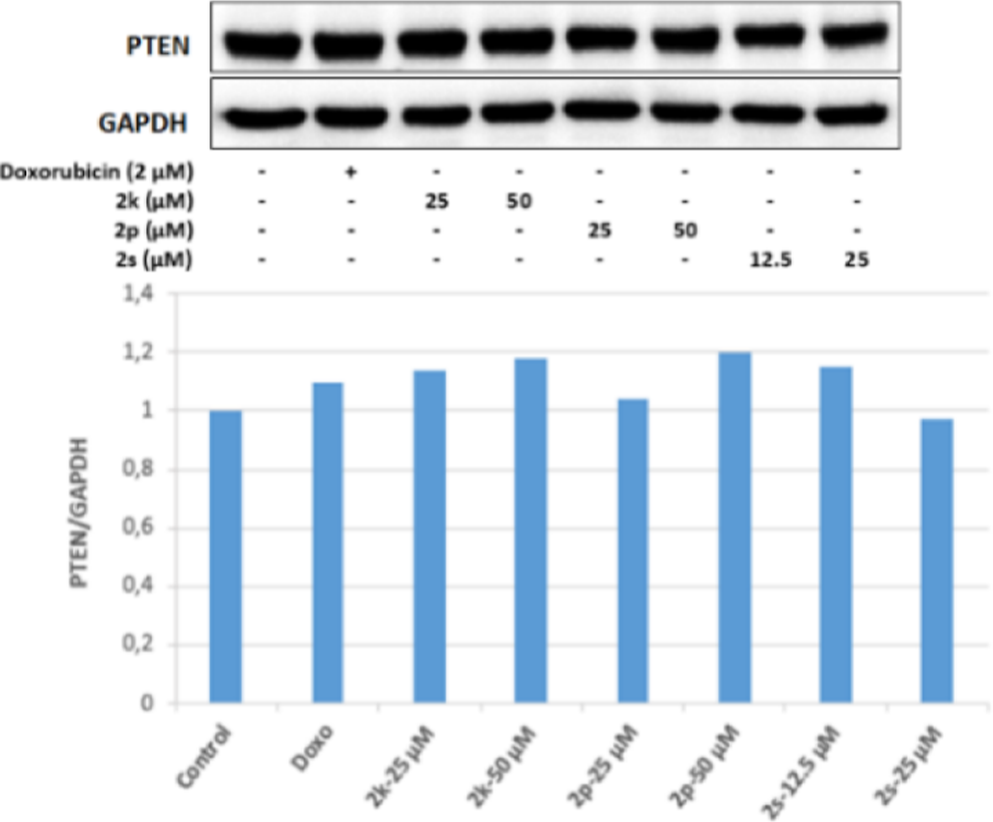
Western blot results of HepG2 cells treated with IC_50_ concentrations of the compounds **2k**, **2p**, **2s**, and Doxorubicin for 24 h on PTEN levels.

**Figure 19:**
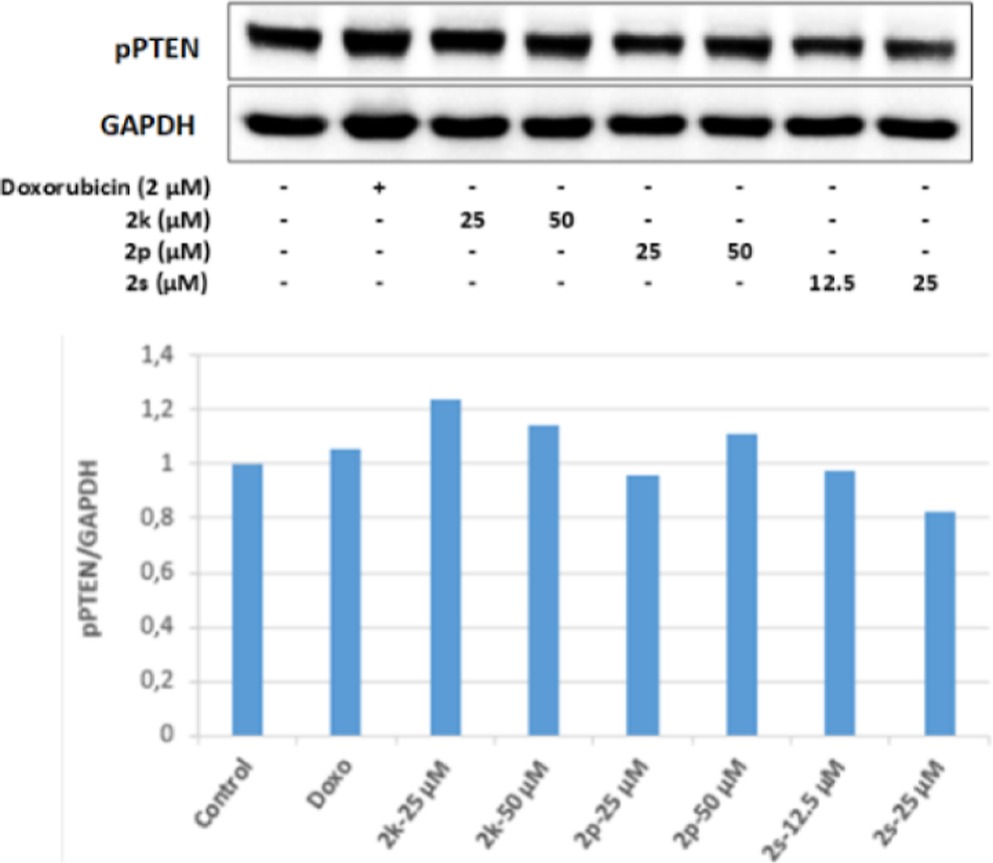
Western blot results of HepG2 cells treated with IC_50_ concentrations of the compounds **2k**, **2p**, **2s**, and Doxorubicin for 24 h on pPTEN levels.

**Figure 20:**
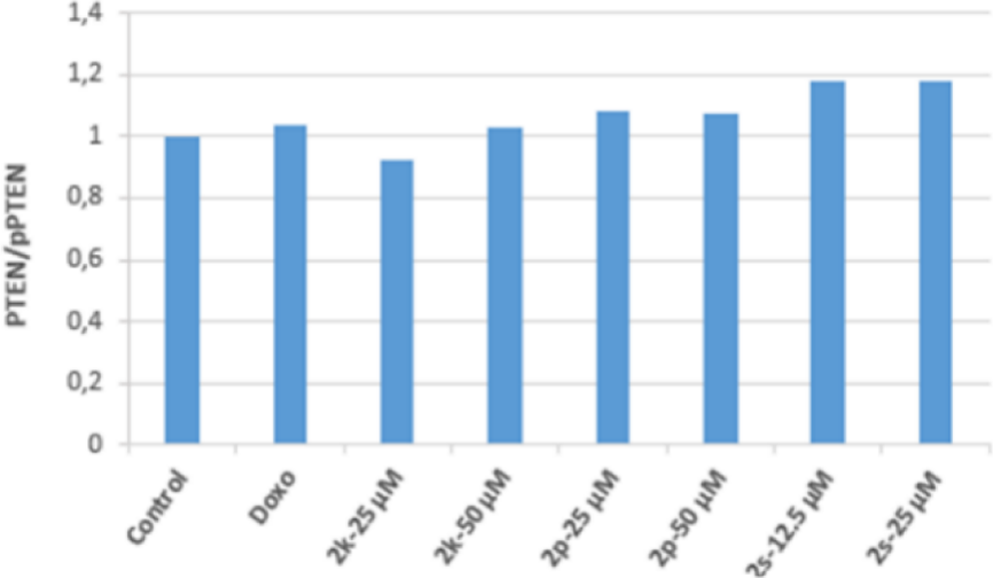
Western blot results of HepG2 cells treated with IC_50_ concentrations of the compounds **2k**, **2p**, **2s**, and Doxorubicin for 24 h on the PTEN/pPTEN ratio.

It was determined that 12.5 and 25 μM concentrations of **2s** in HepG2 cells effectively increased the ratio of active PTEN to inactive p-PTEN (PTEN/p-PTEN). This result shows that 12.5 and 25 μM concentrations of **2s** may suppress the PI3K/Akt pathway by increasing the PTEN/p-PTEN ratio in HepG2 cells. As a result, the active PTEN ratio increased in HepG2 cell groups incubated with 12.5 and 25 μM concentrations of **2s**, decreased the Akt protein level in parallel to this situation, and effectively inhibited PI3K in the same groups. It has been revealed that it performs its antitumoral activity on HepG2 cells by inhibiting the PI3K/Akt signaling pathway (Figures 18–20).

**Figure 21:**
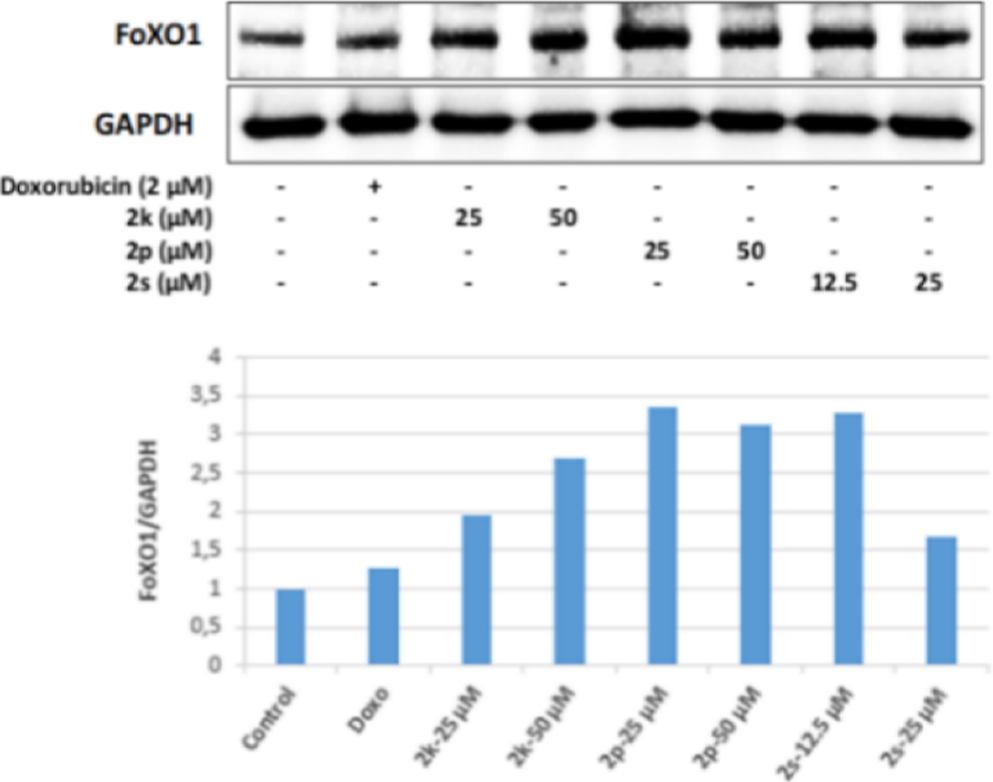
Western blot results of HepG2 cells treated with IC_50_ concentrations of the compounds **2k**, **2p**, **2s**, and Doxorubicin for 24 h on FoXO1 levels.

**Figure 22:**
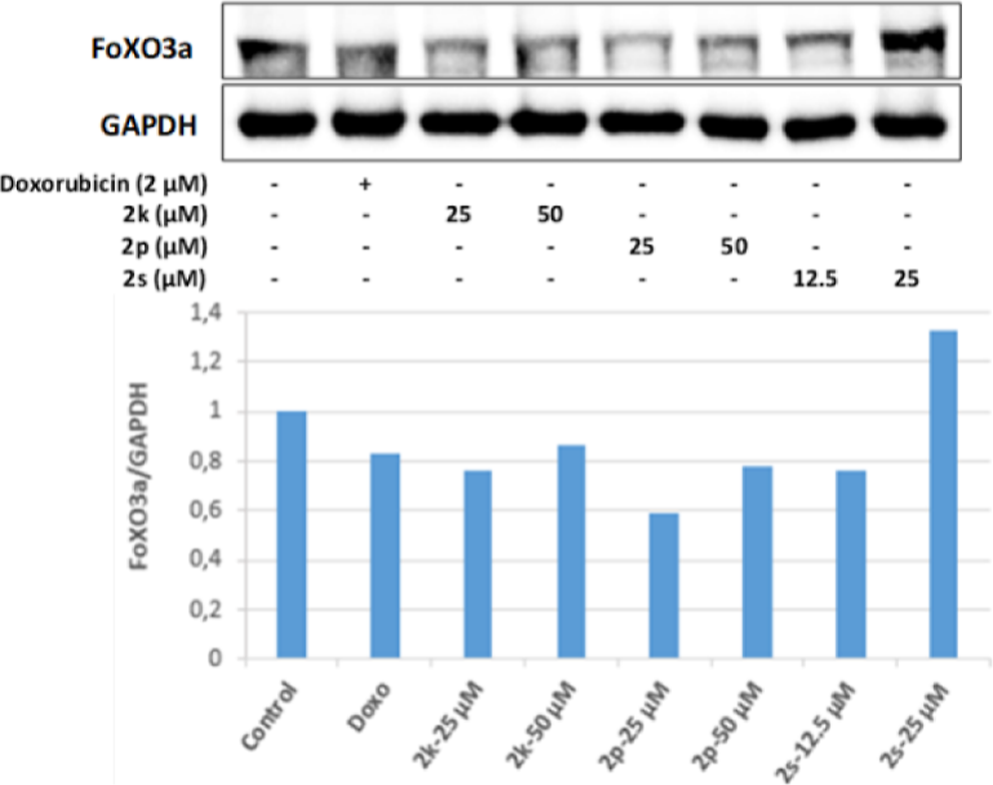
Western blot results of HepG2 cells treated with IC_50_ concentrations of the compounds **2k**, **2p**, **2s**, and Doxorubicin for 24 h on FoXO3a levels.

It was observed that all of the **2k**, **2p**, and **2s** substances, whose antitumoral effects were investigated, increased the FoXO1 protein level in HepG2 cells more effectively than the positive control Doxorubicin. The most effective increase in the FoXO1 protein level was in the cell groups incubated with 25 and 50 μM concentrations of **2p** and 12.5 μM for **2s**, and the increase in FoXO1 protein expression in these groups was detected more than 3 times compared to the control group. On the other hand, it was defined that the 25 μM concentration of **2s** in HepG2 cells effectively increased the FoXO3a protein level as well as the increase in FoXO1 (Figures 21 and 22).

In our study, 25 and 50 μM concentrations of **2k**, 25 μM of **2p**, and 12.5 μM of **2s**, whose antitumoral effects were investigated, increased TXNIP protein levels in HepG2 cells in line with FoXO1 protein levels, thus increasing FoxO protein levels. It was determined that TXNIP stimulation occurred as a result of the activation of the pathway.

**Figure 23:**
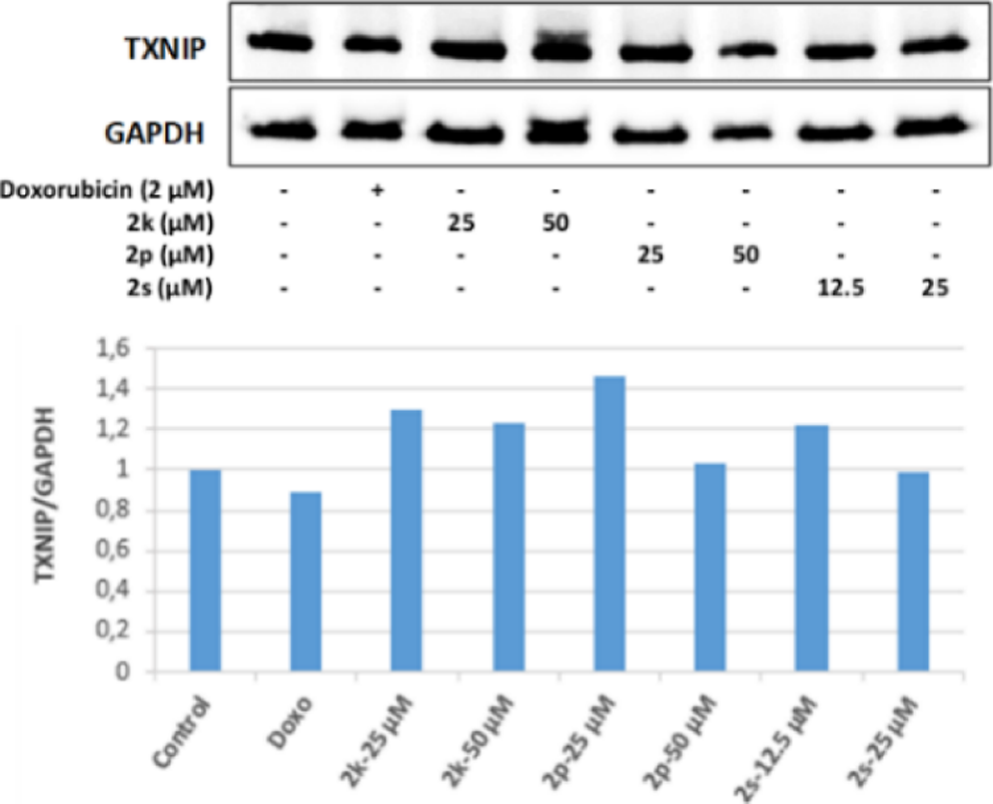
Western blot results of HepG2 cells treated with IC_50_ concentrations of the compounds **2k**, **2p**, **2s**, and Doxorubicin for 24 h on TXNIP levels.

**Figure 24:**
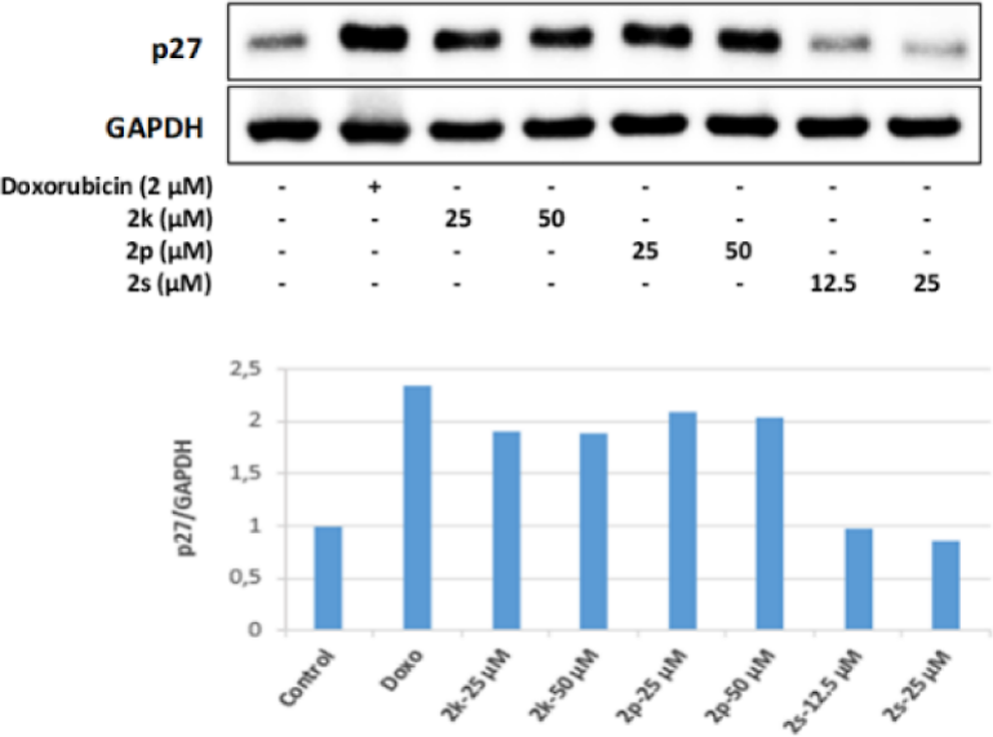
Western blot results of HepG2 cells treated with IC_50_ concentrations of the compounds **2k**, **2p**, **2s**, and Doxorubicin for 24 h on p27 levels.

In this study, 25 and 50 μM concentrations of **2k** and **2p**, whose antitumoral effects were investigated, were found to increase p27 protein levels effectively following FoXO1 and TXNIP protein levels in HepG2 cells. These results revealed that **2k** and **2s** exert their antiproliferative effects on HepG2 cells by increasing the level of p27 protein, which keeps the cell cycle in the G1 phase (Figures 23 and 24).

Finally, it was determined that **2k**, **2p**, and **2s** substances, whose antitumoral activities were investigated in our study, exert their antitumoral effects on HepG2 cells through different cellular pathways. Accordingly, it has been observed that **2s** exerts its antiproliferative effect on HepG2 cells by inhibiting PI3K and Akt levels and by inhibiting the PI3K/Akt signaling pathway by reducing the phosphorylation of PTEN. It has been determined that **2k** and **2p** exert their antiproliferative effects on HepG2 cells through the activation of the FoxO pathway by increasing FoXO1, TXNIP, and p27 protein levels.

## 3 Experimental Section

### 3.1 Chemistry

All chemicals were bought from Sigma-Aldrich. All other chemicals and solvents were purchased from Merck. Melting points were taken on Schmelzpunktbestimmer 9300 SMP II apparatus and were uncorrected. Synthesis of these compounds was carried out in the AREX-6 DIGITAL PRO heating magnetic stirrer. Merck silica gel 60 F254 plates were used for analytical TLC. The purity of the compounds was controlled on TLC plates precoated with silica gel G in a solvent system comprising petroleum ether: ethyl acetate (40:60. v/v. t: 25 °C) mixture as eluent. The spots were located under UV light (254 nm). Ethanol and methanol were used in purification studies. ^1^H-(400 MHz) and ^13^C NMR spectra (100 MHz) were obtained on a BRUKER AM 400 spectrometer in (d6) DMSO solution at the Erciyes University Technology Research and Application Center. MS spectra were carried out on an LC/MS high-resolution time of flight (Q-TOF) Agilent 1200/6530 instrument at the Atatürk University-East Anatolian High Technology Research and Application Center (DAYTAM).

#### 3.1.1 Preparation of the Tetracaine Hydrazide (**1**)

Tetracaine (0.01 mol) and hydrazine hydrate (80%, 20 mL) were refluxed in an ethanolic medium. After that, the reaction mixture was then cooled, diluted with water, and allowed to stand overnight. The precipitated solid was dried and recrystallized from ethanol.

#### 3.1.2 General Procedure for the Synthesis of 4-(butylamino)-*N*′-[(substituted phenyl/thiophene/pyridine)methylidene]benzohydrazide (**2a–t**)

Tetracaine hydrazide (1) (0.01 mol) and substituted aromatic aldehyde (0.011 mol) were refluxed for 12 h with ethanol (20 mL) and with a few drops of glacial acetic acid. The end of the reaction was controlled with TLC, and ethanol was evaporated and washed with cold ethanol. The solid compound was filtered, dried, and recrystallized from ethanol.

##### 3.1.2.1 4-(Butylamino)-*N*′-[(pyridin-2-yl)methylidene]benzohydrazide (**2a**)

Yellow solid, yield 85%, mp 106 °C, *R*_*f*_ 0.11; FT-IR ν max. (cm^–1^): 3305 (N–H), 2954 (Arom C–H), 2927 (Aliph. C–H), 1639 (C=O), 1604 (C=N), 1554, 1527, 1469, 1431 (arom. C=C, N–H, C–N), 1269 (C–O); ^1^H NMR (400 MHz. DMSO-*d*_6_) δ 0.89 (t, *J* = 12 Hz, 3H, −CH_3_), 1.36 (m, 2H, −CH_2_−), 1.52 (m, 2H, −CH_2_−), 3.06 (t, *J* = 20 Hz, 2H, −CH_2_−), 6.36 (s, 1H, −NH−), 6.48–7.94 (m, 8H, Ar–H), 8.43 (s, 1H, −N=CH−), 11.72 (s, 1H, −CO–NH−); ^13^C NMR (100 MHz. DMSO-*d*_6_) δ 164.19 (C=O), 153.78 (C1), 152.78 (C7), 149.75 (C8), 146.57 (C=N), 137.44 (C10), 130.03 (C4), 124.97 (C5), 124.71 (C3), 120.57 (C11), 118.68 (C9), 111.74 (C2), 111.22 (C6), 42.42 (CH_2_), 30.98 (CH_2_), 20.15 (CH_2_), 14.17 (CH_3_); HR-MS (ESI): *m/z* calcd for C_17_H_20_N_4_O [M + H]^+^, 297.1709; found, 297.1716.

##### 3.1.2.2 4-(Butylamino)-*N*′-[(pyridin-3-yl)methylidene]benzohydrazide (**2b**)

Pale yellow solid, yield 87%, mp 140 °C, *R*_*f*_ 0.10; FT-IR ν max. (cm^–1^): 3290 (N–H), 2927 (Arom C–H), 2866 (Aliph. C–H), 1639 (C=O), 1600 (C=N), 1554, 1523, 1477, 1415 (arom. C=C, N–H, C–N), 1273 (C–O); ^1^H NMR (400 MHz. DMSO-*d*_6_) δ 0.89 (t, *J* = 12 Hz, 3H, −CH_3_), 1.37 (m, 2H, −CH_2_−), 1.53 (m, 2H, −CH_2_−), 3.06 (t, *J* = 20 Hz, 2H, −CH_2_−), 6.36 (s, 1H. −NH−), 6.59–8.58 (m, 8H, Ar–H), 8.82 (s, 1H, −N=CH−), 11.68 (s, 1H, −CO–NH−); ^13^C NMR (100 MHz. DMSO-*d*_6_) δ 164.19 (C=O), 152.67 (C1), 150.65 (C9), 148.78 (C8), 143.77 (C=N), 134.10 (C4), 131.00 (C11), 129.92 (C7), 124.55 (C5), 124.55 (C3), 118.76 (C10), 111.25 (C2), 111.25 (C6), 42.44 (CH_2_), 30.98 (CH_2_), 20.14 (CH_2_), 14.14 (CH_3_); HR-MS (ESI): *m/z* calcd for C_17_H_20_N_4_O [M + H]^+^, 297.1709; found, 297.1712.

##### 3.1.2.3 4-(Butylamino)-*N*′-[(pyridin-4-yl)methylidene]benzohydrazide (**2c**)

Yellow solid, yield 97%, mp 116 °C, *R*_*f*_ 0.21; FT-IR ν max. (cm^–1^): 3240 (N–H), 2958 (Arom C–H), 2931 (Aliph. C–H), 1643 (C=O), 1600 (C=N), 1554, 1527, 1473, 1415 (arom. C=C, N–H, C–N), 1277 (C–O); ^1^H NMR (400 MHz. DMSO-*d*_6_) δ 0.90 (t, *J* = 16 Hz, 3H, −CH_3_), 1.38 (m, 2H, −CH_2_−), 1.53 (m, 2H, −CH_2_−), 3.06 (t, *J* = 16 Hz, 2H, −CH_2_−), 6.39 (s, 1H, −NH−), 6.59–8.63 (m, 8H, Ar–H), 8.37 (s, 1H, −N=CH−), 11.76 (s, 1H, −CO–NH−); ^13^C NMR (100 MHz. DMSO-*d*_6_) δ 172.92 (C=O), 152.77 (C1), 150.46 (C9), 150.46 (C10), 142.40 (C=N), 131.66 (C4), 130.06 (C5), 130.06 (C3), 121.46 (C8), 121.46 (C11), 118.58 (C7), 111.24 (C2), 111.24 (C6), 42.41 (CH_2_), 30.98 (CH_2_), 20.15 (CH_2_), 14.17 (CH_3_); HR-MS (ESI): *m/z* calcd for C_17_H_20_N_4_O [M + H]^+^, 297.1709; found, 297.1708.

##### 3.1.2.4 4-(Butylamino)-*N*′-[(2-chlorophenyl)methylidene]benzohydrazide (**2d**)

Beige solid, yield 90%, mp 151 °C, *R*_*f*_ 0.73; FT-IR ν max. (cm^–1^): 3244 (N–H), 2954 (Arom C–H), 2927 (Aliph. C–H), 1627 (C=O), 1597 (C=N), 1543, 1519, 1489, 1473 (arom. C=C, N–H, C–N), 1273 (C–O), 1141 (C–Cl); ^1^H NMR (400 MHz. DMSO-*d*_6_) δ 0.90 (t, *J* = 12 Hz, 3H, −CH_3_), 1.37 (m, 2H, −CH_2_−), 1.52 (m, 2H, −CH_2_−), 3.05 (t, *J* = 16 Hz, 2H, −CH_2_−), 6.35 (s, 1H, −NH−), 6.58–7.72 (m, 8H, Ar–H), 8.38 (s, 1H, −N=CH−), 11.57 (s, 1H, −CO–NH−); ^13^C NMR (100 MHz. DMSO-*d*_6_) δ 163.68 (C=O), 152.53 (C1), 145.08 (C=N), 135.92 (C8), 134.56 (C4), 134.03 (C7), 128.96 (C5), 129.82 (C10), 129.32 (C12), 128.96 (C3), 127.06 (C11), 119.15 (C9), 111.16 (C2), 111.16 (C6), 42.46 (CH_2_), 31.05 (CH_2_), 20.21 (CH_2_), 14.22 (CH_3_); HR-MS (ESI): *m/z* calcd for C_18_H_20_ClN_3_O [M + H]^+^, 330.1367; found, 330.1367.

##### 3.1.2.5 4-(Butylamino)-*N*′-[(3-chlorophenyl)methylidene]benzohydrazide (**2e**)

Beige solid, yield 81%, mp 132 °C, *R*_*f*_ 0.68; FT-IR ν max. (cm^–1^): 3221 (N–H), 2954 (Arom C–H), 2924 (Aliph. C–H), 1627 (C=O), 1612 (C=N), 1539, 1519, 1469, 1419 (arom. C=C, N–H, C–N), 1269 (C–O), 1141 (C–Cl); ^1^H NMR (400 MHz. DMSO-*d*_6_) δ 0.90 (t, *J* = 16 Hz, 3H, −CH_3_), 1.38 (m, 2H, −CH_2_−), 1.53 (m, 2H, −CH_2_−), 3.05 (t, *J* = 20 Hz, 2H, −CH_2_−), 6.35 (s, 1H, −NH−), 6.58–7.74 (m, 8H, Ar–H), 8.37 (s, 1H, −N=CH−), 11.63 (s, 1H, −CO–NH−); ^13^C NMR (100 MHz. DMSO-*d*_6_) δ 164.08 (C=O), 152.63 (C1), 144.98 (C=N), 137.17 (C7), 134.08 (C4), 131.15 (C9), 129.87 (C5), 129.87 (C3), 126.52 (C12), 126.17 (C8), 125.03 (C10), 118.78 (C11), 111.23 (C2), 111.23 (C6), 42.42 (CH_2_), 30.97 (CH_2_), 20.15 (CH_2_), 14.17 (CH_3_); HR-MS (ESI): *m/z* calcd for C_18_H_20_ClN_3_O [M + H]^+^, 330.1367; found, 330.1369.

##### 3.1.2.6 4-(Butylamino)-*N*′-[(4-chlorophenyl)methylidene]benzohydrazide (**2f**)

Beige solid, yield 83%, mp 147 °C, *R*_*f*_ 0.63; FT-IR ν max. (cm^–1^): 3240 (N–H), 2954 (Arom C–H), 2931 (Aliph. C–H), 1627 (C=O), 1597 (C=N), 1543, 1519, 1465, 1438 (arom. C=C, N–H, C–N), 1273 (C–O), 1141 (C–Cl); ^1^H NMR (400 MHz. DMSO-*d*_6_) δ 0.90 (t, *J* = 16 Hz, 3H, −CH_3_), 1.38 (m, 2H, −CH_2_−), 1.53 (m, 2H, −CH_2_−), 3.06 (t, *J* = 16 Hz, 2H, −CH_2_−), 6.36 (s, 1H, −NH−), 6.59–7.75 (m, 8H, Ar–H), 8.81 (s, 1H, −N=CH−), 11.74 (s, 1H, −CO–NH−); ^13^C NMR (100 MHz. DMSO-*d*_6_) δ 164.03 (C=O), 152.67 (C1), 142.65 (C=N), 132.15 (C7), 133.42 (C4), 127.34 (C9), 128.00 (C5), 128.00 (C3), 130.26 (C12), 129.95 (C8), 131.68 (C10), 127.34 (C11), 111.22 (C2), 111.22 (C6), 42.42 (CH_2_), 30.98 (CH_2_), 20.15 (CH_2_), 14.17 (CH_3_); HR-MS (ESI): *m/z* calcd for C_18_H_20_ClN_3_O [M + H]^+^, 330.1367; found, 330.1371.

##### 3.1.2.7 4-(Butylamino)-*N*′-[(2-chloro-6-fluorophenyl)methylidene]benzohydrazide (**2g**)

White solid, yield 81%, mp 146 °C, *R*_*f*_ 0.71; FT-IR ν max. (cm^–1^): 3240 (N–H), 2951 (Arom C–H), 2931 (Aliph. C–H), 1631 (C=O), 1597 (C=N), 1543, 1519, 1462, 1438 (arom. C=C, N–H, C–N), 1269 (C–O), 1141 (C–Cl), 1072 (C–F); ^1^H NMR (400 MHz. DMSO-*d*_6_) δ 0.90 (t, *J* = 12 Hz, 3H, −CH_3_), 1.38 (m, 2H, −CH_2_−), 1.53 (m, 2H, −CH_2_−), 3.06 (t, *J* = 20 Hz, 2H, −CH_2_−), 6.37 (s, 1H, −NH−), 6.58–7.75 (m, 8H, Ar–H), 8.64 (s, 1H, −N=CH−), 11.71 (s, 1H, −CO–NH−); ^13^C NMR (100 MHz. DMSO-*d*_6_) δ 161.80 (C=O), 159.24 (C12), 152.61 (C1), 139.46 (C=N), 134.17 (C4), 131.76 (C10), 130.00 (C7), 126.46 (C5), 126.46 (C3), 118.98 (C8), 116.11 (C11), 115.89 (C9), 111.12 (C2), 111.12 (C6), 42.46 (CH_2_), 31.07 (CH_2_), 20.21 (CH_2_), 14.22 (CH_3_); HR-MS (ESI): *m/z* calcd for C_18_H_19_ClFN_3_O [M + H]^+^, 348.1273; found, 348.1280.

##### 3.1.2.8 4-(Butylamino)-*N*′-[(2-fluorophenyl)methylidene]benzohydrazide (**2h**)

White solid, yield 83%, mp 123 °C, *R*_*f*_ 0.70; FT-IR ν max. (cm^–1^): 3244 (N–H), 2951 (Arom C–H), 2931 (Aliph. C–H), 1627 (C=O), 1597 (C=N), 1543, 1519, 1481, 1454 (arom. C=C, N–H, C–N), 1273 (C–O), 1099 (C–F); ^1^H NMR (400 MHz. DMSO-*d*_6_) δ 0.90 (t, *J* = 16 Hz, 3H, −CH_3_), 1.38 (m, 2H, −CH_2_−), 1.53 (m, 2H, −CH_2_−), 3.06 (t, *J* = 12 Hz, 2H, −CH_2_−), 6.37 (s, 1H, −NH−), 6.58–7.93 (m, 8H, Ar–H), 8.64 (s, 1H, −N=CH−), 11.64 (s, 1H, −CO–NH−); ^13^C NMR (100 MHz. DMSO-*d*_6_) δ 162.32 (C=O), 159.85 (C8), 152.59 (C1), 139.08 (C=N), 132.08 (C4), 129.87 (C7), 126.70 (C5), 126.70 (C3), 125.76 (C10), 122.65 (C11), 119.02 (C12), 116.25 (C9), 111.17 (C2), 111.17 (C6), 42.46 (CH_2_), 31.05 (CH_2_), 20.20 (CH_2_), 14.21 (CH_3_); HR-MS (ESI): *m/z* calcd for C_18_H_20_FN_3_O [M + H]^+^, 314.1663; found, 314.1663.

##### 3.1.2.9 4-(Butylamino)-*N*′-[(3-fluorophenyl)methylidene]benzohydrazide (**2i**)

White solid, yield 85%, mp 139 °C, *R*_*f*_ 0.75; FT-IR ν max. (cm^–1^): 3224 (N–H), 2954 (Arom C–H), 2924 (Aliph. C–H), 1624 (C=O), 1612 (C=N), 1543, 1519, 1477, 1446 (arom. C=C, N–H, C–N), 1269 (C–O), 1060 (C–F); ^1^H NMR (400 MHz. DMSO-*d*_6_) δ 0.90 (t, *J* = 16 Hz, 3H, −CH_3_), 1.38 (m, 2H, −CH_2_−), 1.53 (m, 2H, −CH_2_−), 3.06 (t, *J* = 12 Hz, 2H, −CH_2_−), 6.36 (s, 1H, −NH−), 6.59–7.74 (m, 8H, Ar–H), 8.40 (s, 1H, −N=CH−), 11.62 (s, 1H, −CO–NH−); ^13^C NMR (100 MHz. DMSO-*d*_6_) δ 164.05 (C=O), 113.26 (C8), 152.63 (C1), 145.35 (C=N), 137.56 (C4), 131.35 (C7), 123.96 (C5), 123.96 (C3), 117.09 (C10), 129.88 (C11), 118.81 (C12), 161.63 (C9), 111.23 (C2), 111.23 (C6), 42.42 (CH_2_), 30.98 (CH_2_), 20.15 (CH_2_), 14.16 (CH_3_); HR-MS (ESI): *m/z* calcd for C_18_H_20_FN_3_O [M + H]^+^, 314.1663; found, 314.1664.

##### 3.1.2.10 4-(Butylamino)-*N*′-[(4-fluorophenyl)methylidene]benzohydrazide (**2j**)

Beige solid, yield 84%, mp 129 °C, *R*_*f*_ 0.65; FT-IR ν max. (cm^–1^): 3228 (N–H), 2954 (Arom C–H), 2927 (Aliph. C–H), 1624 (C=O), 1604 (C=N), 1546, 1523, 1504, 1469 (arom. C=C, N–H, C–N), 1269 (C–O), 1060 (C–F); ^1^H NMR (400 MHz. DMSO-*d*_6_) δ 0.89 (t, *J* = 16 Hz, 3H, −CH_3_), 1.37 (m, 2H, −CH_2_−), 1.52 (m, 2H, −CH_2_−), 3.06 (t, *J* = 12 Hz, 2H, −CH_2_−), 6.32 (s, 1H, −NH−), 6.58–7.75 (m, 8H, Ar–H), 8.38 (s, 1H, −N=CH−), 11.52 (s, 1H, −CO–NH−); ^13^C NMR (100 MHz. DMSO-*d*_6_) δ 164.60 (C=O), 162.14 (C10), 152.54 (C1), 145.60 (C=N), 131.56 (C4), 129.78 (C7), 129.60 (C8), 129.60 (C12), 129.52 (C5), 129.52 (C3), 116.36 (C9), 116.14 (C11), 111.21 (C2), 111.21 (C6), 42.44 (CH_2_), 30.99 (CH_2_), 20.15 (CH_2_), 14.17 (CH_3_); HR-MS (ESI): *m/z* calcd for C_18_H_20_FN_3_O [M + H]^+^, 314.1663; found, 314.1667.

##### 3.1.2.11 4-(Butylamino)-*N*′-[(4-bromophenyl)methylidene]benzohydrazide (**2k**)

Beige solid, yield 80%, mp 167 °C, *R*_*f*_ 0.63; FT-IR ν max. (cm^–1^): 3244 (N–H), 2951 (Arom C–H), 2927 (Aliph. C–H), 1627 (C=O), 1593 (C=N), 1543, 1519, 1485, 1473 (arom. C=C, N–H, C–N), 1273 (C–O), 1192 (C–Br); ^1^H NMR (400 MHz. DMSO-*d*_6_) δ 0.89 (t, *J* = 4 Hz, 3H, −CH_3_), 1.36 (m, 2H, −CH_2_−), 1.52 (m, 2H, −CH_2_−), 3.05 (t, *J* = 4 Hz, 2H, −CH_2_−), 6.34 (s, 1H, −NH−), 6.60–7.73 (m, 8H, Ar–H), 8.35 (s, 1H, −N=CH−), 11.60 (s, 1H, −CO–NH−); ^13^C NMR (100 MHz. DMSO-*d*_6_) δ 163.81 (C=O), 152.56 (C1), 145.29 (C=N), 134.32 (C4), 132.21 (C7), 129.84 (C9), 129.84 (C11), 129.22 (C8), 129.22 (C12), 123.35 (C5), 123.35 (C3), 119.04 (C10), 111.18 (C2), 111.18 (C6), 42.45 (CH_2_), 31.02 (CH_2_), 20.20 (CH_2_), 14.21 (CH_3_); HR-MS (ESI): *m/z* calcd for C_18_H_20_BrN_3_O [M + H]^+^, 374.0862; found, 374.0866.

##### 3.1.2.12 4-(Butylamino)-*N*′-[(4-cyanophenyl)methylidene]benzohydrazide (**2l**)

Yellow solid, yield 87%, mp 217 °C, *R*_*f*_ 0.52; FT-IR ν max. (cm^–1^): 3244 (N–H), 2954 (Arom C–H), 2908 (Aliph. C–H), 2225 (C≡N), 1643 (C=O), 1604 (C=N), 1539, 1519, 1469, 1411 (arom. C=C, N–H, C–N), 1273 (C–O); ^1^H NMR (400 MHz. DMSO-*d*_6_) δ 0.91 (t, *J* = 16 Hz, 3H, −CH_3_), 1.39 (m, 2H, −CH_2_−), 1.54 (m, 2H, −CH_2_−), 3.06 (t, *J* = 4 Hz, 2H, −CH_2_−), 6.38 (s, 1H. −NH−), 6.59–7.91 (m, 8H, Ar–H), 8.44 (s, 1H, −N=CH−), 11.72 (s, 1H, −CO–NH−); ^13^C NMR (100 MHz. DMSO-*d*_6_) δ 163.73 (C=O), 152.66 (C1), 144.23 (C=N), 139.67 (C9), 139.67 (C11), 133.17 (C4), 130.00 (C7), 127.84 (C5), 127.84 (C3), 119.22 (C8), 119.22 (C12), 118.98 (C≡N), 111.87 (C10), 111.16 (C2), 111.16 (C6), 42.46 (CH_2_), 31.07 (CH_2_), 20.20 (CH_2_), 14.22 (CH_3_); HR-MS (ESI): *m/z* calcd for C_19_H_20_N_4_O [M + H]^+^, 321.1709; found, 321.1713.

##### 3.1.2.13 4-(Butylamino)-*N*′-{[2-(trifluoromethyl)phenyl]methylidene}benzohydrazide (**2m**)

Beige solid, yield 81%, mp 162 °C, *R*_*f*_ 0.75; FT-IR ν max. (cm^–1^): 3240 (N–H), 2954 (Arom C–H), 2931 (Aliph. C–H), 1624 (C=O), 1593 (C=N), 1543, 1519, 1473, 1450 (arom. C=C, N–H, C–N), 1273 (C–O), 1060 (C–F); ^1^H NMR (400 MHz. DMSO-*d*_6_) δ 0.89 (t, *J* = 16 Hz, 3H, −CH_3_), 1.36 (m, 2H, −CH_2_−), 1.52 (m, 2H, −CH_2_−), 3.06 (t, *J* = 16 Hz, 2H, −CH_2_−), 6.39 (s, 1H, −NH−), 6.60–8.22 (m, 8H, Ar–H), 8.78 (s, 1H, −N=CH−), 11.85 (s, 1H, −CO–NH−); ^13^C NMR (100 MHz. DMSO-*d*_6_) δ 164.04 (C=O), 152.70 (C1), 141.45 (C=N), 118.82 (C9), 127.14 (C11), 133.18 (C4), 129.99 (C7), 127.21 (C5), 127.21 (C3), 130.15 (C8), 126.84 (C12), 126.23 (CF_3_), 132.98 (C10), 111.16 (C2), 111.16 (C6), 42.45 (CH_2_), 31.04 (CH_2_), 20.18 (CH_2_), 14.18 (CH_3_); HR-MS (ESI): *m/z* calcd for C_19_H_20_F_3_N_3_O [M + H]^+^, 364.1631; found, 364.1632.

##### 3.1.2.14 4-(Butylamino)-*N*′-{[3-(trifluoromethyl)phenyl]methylidene}benzohydrazide (**2n**)

Beige solid, yield 82%, mp 127 °C, *R*_*f*_ 0.75; FT-IR ν max. (cm^–1^): 3228 (N–H), 2962 (Arom C–H), 2927 (Aliph. C–H), 1627 (C=O), 1612 (C=N), 1543, 1519, 1477, 1431 (arom. C=C, N–H, C–N), 1265 (C–O), 1095 (C–F); ^1^H NMR (400 MHz. DMSO-*d*_6_) δ 0.90 (t, *J* = 16 Hz, 3H, −CH_3_), 1.38 (m, 2H, −CH_2_−), 1.53 (m, 2H, −CH_2_−), 3.06 (t, *J* = 16 Hz, 2H, −CH_2_−), 6.36 (s, 1H, −NH−), 6.59–8.03 (m, 8H, Ar–H), 8.47 (s, 1H, −N=CH−), 11.69 (s, 1H, −CO–NH−); ^13^C NMR (100 MHz. DMSO-*d*_6_) δ 164.04 (C=O), 152.64 (C1), 144.75 (C=N), 130.43 (C9), 126.41 (C11), 131.32 (C4), 136.19 (C7), 129.90 (C5), 129.90 (C3), 125.84 (C8), 130.21 (C12), 123.27 (CF_3_), 118.88 (C10), 111.19 (C2), 111.19 (C6), 42.43 (CH_2_), 31.01 (CH_2_), 20.17 (CH_2_), 14.18 (CH_3_); HR-MS (ESI): *m/z* calcd for C_19_H_20_F_3_N_3_O [M + H]^+^, 364.1631; found, 364.1637.

##### 3.1.2.15 4-(Butylamino)-*N*′-{[4-(trifluoromethyl)phenyl]methylidene}benzohydrazide (**2o**)

Beige solid, yield 86%, mp 192 °C, *R*_*f*_ 0.68; FT-IR ν max. (cm^–1^): 3276 (N–H), 2958 (Arom C–H), 2931 (Aliph. C–H), 1631 (C=O), 1593 (C=N), 1570, 1523, 1508, 1477 (arom. C=C, N–H, C–N), 1269 (C–O), 1064 (C–F); ^1^H NMR (400 MHz. DMSO-*d*_6_) δ 0.90 (t, *J* = 16 Hz, 3H, −CH_3_), 1.37 (m, 2H, −CH_2_−), 1.52 (m, 2H, −CH_2_−), 3.06 (t, *J* = 4 Hz, 2H, −CH_2_−), 6.36 (s, 1H, −NH−), 6.60–7.91 (m, 8H, Ar–H), 8.46 (s, 1H, −N=CH−), 11.71 (s, 1H, −CO–NH−); ^13^C NMR (100 MHz. DMSO-*d*_6_) δ 164.06 (C=O), 152.66 (C1), 144.78 (C=N), 126.00 (C9), 126.00 (C11), 138.97 (C4), 130.00 (C7), 129.69 (C5), 129.69 (C3), 127.88 (C8), 127.88 (C12), 123.17 (CF_3_), 118.83 (C10), 111.20 (C2), 111.20 (C6), 42.43 (CH_2_), 30.99 (CH_2_), 20.16 (CH_2_), 14.13 (CH_3_); HR-MS (ESI): *m/z* calcd for C_19_H_20_F_3_N_3_O [M + H]^+^, 364.1631; found, 364.1636.

##### 3.1.2.16 4-(Butylamino)-*N*′-{[2-(trifluoromethoxy)phenyl]methylidene}benzohydrazide (**2p**)

White solid, yield 84%, mp 153 °C, *R*_*f*_ 0.67; FT-IR ν max. (cm^–1^): 3236 (N–H), 2951 (Arom C–H), 2931 (Aliph. C–H), 1627 (C=O), 1593 (C=N), 1543, 1519, 1477, 1454 (arom. C=C, N–H, C–N), 1246 (C–O), 1072 (C–F); ^1^H NMR (400 MHz. DMSO-*d*_6_) δ 0.90 (t, *J* = 16 Hz, 3H. −CH_3_), 1.36 (m, 2H, −CH_2_−), 1.53 (m, 2H, −CH_2_−), 3.06 (t, *J* = 8 Hz, 2H, −CH_2_−), 6.37 (s, 1H, −NH−), 6.59–8.09 (m, 8H, Ar–H), 8.67 (s, 1H, −N=CH−), 11.74 (s, 1H, −CO–NH−); ^13^C NMR (100 MHz. DMSO-*d*_6_) δ 163.88 (C=O), 156.58 (C8), 152.66 (C1), 147.02 (C=N), 139.50 (C4), 131.73 (C12), 129.91 (C10), 128.45 (C5), 128.45 (C3), 128.17 (C7), 126.97 (C9), 122.21 (OCF_3_), 118.85 (C11), 111.15 (C2), 111.15 (C6), 42.44 (CH_2_), 31.02 (CH_2_), 20.18 (CH_2_), 14.18 (CH_3_); HR-MS (ESI): *m/z* calcd for C_19_H_20_F_3_N_3_O_2_ [M + H]^+^. 380.1580; found, 380.1586.

##### 3.1.2.17 4-(Butylamino)-*N*′-{[3-(trifluoromethoxy)phenyl]methylidene}benzohydrazide (**2q**)

Beige solid, yield 90%, mp 115 °C, *R*_*f*_ 0.73; FT-IR ν max. (cm^–1^): 3228 (N–H), 2927 (Arom C–H), 2873 (Aliph. C–H), 1627 (C=O), 1612 (C=N), 1573, 1546, 1523, 1481 (arom. C=C, N–H, C–N), 1288 (C–O), 1064 (C–F); ^1^H NMR (400 MHz. DMSO-*d*_6_) δ 0.91 (t, *J* = 8 Hz, 3H. −CH_3_), 1.39 (m, 2H, −CH_2_−), 1.54 (m, 2H, −CH_2_−), 3.07 (t, *J* = 4 Hz, 2H, −CH_2_−), 6.36 (s, 1H, −NH−), 6.59–7.75 (m, 8H, Ar–H), 8.44 (s, 1H, −N=CH−), 11.66 (s, 1H, −CO–NH−); ^13^C NMR (100 MHz. DMSO-*d*_6_) δ 163.94 (C=O), 152.64 (C1), 149.24 (C=N), 144.77 (C9), 137.55 (C4), 131.34 (C7), 129.93 (C5), 129.93 (C3), 126.74 (C11), 122.42 (OCF_3_), 119.25 (C12), 118.94 (C10), 118.73 (C8), 111.19 (C2), 111.19 (C6), 42.46 (CH_2_), 31.03 (CH_2_), 20.19 (CH_2_), 14.17 (CH_3_); HR-MS (ESI): *m/z* calcd for C_19_H_20_F_3_N_3_O_2_ [M + H]^+^, 380.1580; found, 380.1584.

##### 3.1.2.18 4-(Butylamino)-*N*′-{[4-(trifluoromethoxy)phenyl]methylidene}benzohydrazide (**2r**)

Beige solid, yield 83%, mp 174 °C, *R*_*f*_ 0.68; FT-IR ν max. (cm^–1^): 3221 (N–H), 2954 (Arom C–H), 2927 (Aliph. C–H), 1627 (C=O), 1612 (C=N), 1543, 1527, 1477, 1415 (arom. C=C, N–H, C–N), 1246 (C–O), 1060 (C–F); ^1^H NMR (400 MHz. DMSO-*d*_6_) δ 0.88 (t, *J* = 16 Hz, 3H, −CH_3_), 1.36 (m, 2H, −CH_2_−), 1.52 (m, 2H, −CH_2_−), 3.04 (t, *J* = 16 Hz, 2H, −CH_2_−), 6.34 (s, 1H, −NH−), 6.58–7.83 (m, 8H, Ar–H), 8.41 (s, 1H, −N=CH−), 11.63 (s, 1H, −CO–NH−); ^13^C NMR (100 MHz. DMSO-*d*_6_) δ 163.76 (C=O), 152.55 (C1), 149.43 (C=N), 144.83 (C10), 134.40 (C4), 132.64 (C7), 129.86 (C5), 129.86 (C3), 129.11 (C8), 129.11 (C12), 121.69 (OCF_3_), 119.16 (C11), 119.16 (C9), 111.15 (C2), 111.15 (C6), 42.47 (CH_2_), 31.06 (CH_2_), 20.20 (CH_2_), 14.17 (CH_3_); HR-MS (ESI): *m/z* calcd for C_19_H_20_F_3_N_3_O_2_ [M + H]^+^, 380.1580; found, 380.1586.

##### 3.1.2.19 4-(Butylamino)-*N*′-{[3,5-bis(trifluoromethyl)phenyl]methylidene}-benzohydrazide (**2s**)

White solid, yield 87%, mp 196 °C, *R*_*f*_ 0.89; FT-IR ν max. (cm^–1^): 3221 (N–H), 2931 (Arom C–H), 2831 (Aliph. C–H), 1639 (C=O), 1608 (C=N), 1550, 1523, 1465 (arom. C=C, N–H, C–N), 1273 (C–O), 1068 (C–F); ^1^H NMR (400 MHz. DMSO-*d*_6_) δ 0.88 (t, *J* = 12 Hz, 3H, −CH_3_), 1.36 (m, 2H, −CH_2_−), 1.52 (m, 2H, −CH_2_−), 3.05 (t, *J* = 4 Hz, 2H, −CH_2_−), 6.40 (s, 1H, −NH−), 6.58–8.35 (m, 8H, Ar–H), 8.50 (s, 1H, −N=CH−), 11.91 (s, 1H, −CO–NH−); ^13^C NMR (100 MHz. DMSO-*d*_6_) δ 164.06 (C=O), 152.78 (C1), 143.12 (C=N), 137.83 (C4), 131.38 (C11), 131.38 (C9), 131.05 (C7), 130.03 (C5), 130.03 (C3), 127.42 (C8), 127.42 (C12), 124.93 (C10), 122.88 (CF_3_), 122.22 (CF_3_), 111.21 (C2), 111.21 (C6), 42.38 (CH_2_), 30.94 (CH_2_), 20.13 (CH_2_), 14.14 (CH_3_); HR-MS (ESI): *m/z* calcd for C_20_H_19_F_6_N_3_O [M + H]^+^, 432.1505; found, 432.1507.

##### 3.1.2.20 4-(Butylamino)-*N*′-[(4-phenylthiophen-2-yl)methylidene]benzohydrazide (**2t**)

Yellow solid, yield 68%, mp 181 °C, *R*_*f*_ 0.57; FT-IR ν max. (cm^–1^): 3251 (N–H), 2951 (Arom C–H), 2927 (Aliph. C–H), 1639 (C=O), 1600 (C=N), 1546, 1512, 1496, 1465 (arom. C=C, N–H, C–N), 1265 (C–O), 1184 (C–S); ^1^H NMR (400 MHz. DMSO-*d*_6_) δ 0.91 (t, *J* = 16 Hz, 3H, −CH_3_), 1.37 (m, 2H, −CH_2_−), 1.54 (m, 2H, −CH_2_−), 3.07 (t, *J* = 20 Hz, 2H, −CH_2_−), 6.33 (s, 1H, −NH−), 6.59–7.95 (m, 8H, Ar–H), 8.66 (s, 1H, −N=CH−), 11.52 (s, 1H, −CO–NH−); ^13^C NMR (100 MHz. DMSO-*d*_6_) δ 164.79 (C=O), 152.47 (C1), 142.06 (C=N), 140.93 (C7), 140.86 (C9), 134.99 (C4), 129.38 (C5), 129.38 (C3), 129.09 (C11), 127.87 (C10), 126.38 (C14), 126.38 (C15), 123.49 (C13), 123.49 (C12), 123.49 (C16), 119.40 (C8), 111.12 (C2), 111.12 (C6), 42.50 (CH_2_), 31.11 (CH_2_), 20.24 (CH_2_), 14.26 (CH_3_); HR-MS (ESI): *m/z* calcd for C_20_H_19_F_6_N_3_O [M + H]^+^, 378.1634; found, 378.1635.

### 3.2 Biological Analyses

#### 3.2.1 Cell Culture and MTT Cytotoxicity Assay

HepG2 and Colo-205 cell lines were obtained from the American Type Culture Collection (VA, USA). The cells were grown in DMEM supplemented with 10% FBS and 100 IU/mL penicillin–streptomycin. The cultures were maintained at 37 °C in a humidified atmosphere of 5% CO^2^. Doxorubicin was utilized as a positive control reference standard for cell lines. The effect of synthesized molecules (**2a–t**) on HepG2 and Colo-205 cell viability was detected by MTT assay. HepG2 cells were seeded into a 96-well culture dish at a density of 15,000 cells/well in 0.1 mL of DMEM medium. Cells were incubated in a sterile carbon dioxide incubator for 24 h. After 24 h, the medium was replaced with fresh medium. Test substances that are thought to affect cell viability were added to the culture medium at various concentrations of compounds (**2a–t**) (12.5, 25, 50, 100, 200, and 400 μM), and the cells were incubated for 24 and 48 h. At the end of the incubation periods, the medium containing the substances was discarded and 10 μL MTT reagent in 100 μL medium was added to each well, and plates were allowed to incubate for 2 h. Formazan crystals formed as a result of incubation in viable cells were dissolved in 100 μL DMSO and absorbance was detected in a microplate reader at a wavelength of 570 nm.^22^

#### 3.2.2 Quantitative Real-Time PCR

Expression levels of the Bax and caspase-3 genes which were thought to play an important role in apoptosis of the compounds showing anticancer activity in the HepG2 and Colo-205 cell line were analyzed by quantitative real-time polymerase chain reaction (qRT-PCR). For this purpose, the cells were seeded in 100 × 20 mm cell culture Petri dishes and incubated at 37 °C. When the cell growth reaches 70–80% fullness in the bottom of the Petri dish, the medium was replaced with a new medium and the test substances were incubated at the appropriate concentration for 24 h and determined by the MTT test. Total RNA isolation was performed by the manufacturer’s protocol, and total RNA was purified from cultured cells using a trizole RNeasy mini kit (Qiagen Inc., CA, USA). The RNA concentration was measured using a Thermo Scientific NanoDropTM Spectrophotometer (Thermo Fisher Scientific, DA USA). cDNA was synthesized using the Evoscript Universal reverse transcriptase kit (Roche, Switzerland). For qRT-PCR analysis, reference and target genes were selected from RealTime Ready Catalog Assays (Roche, Switzerland). qPCR was performed with the LightCycler 480 System (Roche, Switzerland) according to the manufacturer’s protocol to measure the mRNA expression levels of the targeted genes. Pre-incubation at 95 °C for 10 min, 45 repetitions at 95 °C for 10 s, 60 °C for 30 s, 72 °C for a 1 s annealing cycle, and 40 °C for 30 s of cooling were recorded on the real-time PCR instrument. The Ct cycle was used to determine the expression level and control and 3i- and 3j-treated cells.^22^

#### 3.2.3 Western Blot Assay

In HepG2 and Colo-205 cells, substances that are as efficient as the reference substance (doxorubicin) in cell viability were chosen and the mechanism of how these substances affect cell viability was researched. It was also included as part of a positive control study to test compound effects that were selected and did not affect cell viability. HepG2 and Colo-205 cells were cultured in a 100 mm culture dish. After 24 h, the growth medium was replaced, and cells were treated with compounds **2k, 2p, 2s, 2f**, and **2m** or doxorubicin at their IC_50_ values for 24 h. At the end of 24 h incubation, samples were scrapped and collected for Western blot analysis. The compounds were then applied to the cells at varying concentrations and for varying durations. At the end of the period, the medium was discarded and the cells were homogenized with a lysis solution. Total protein was determined in the homogenates. Protein samples were analyzed by the classical Western blot method.^20,21,25^

## 4 Conclusions

In search of compounds with anticancer properties, we have designed and synthesized a series of new Tetracaine hydrazide–hydrazones. These compounds were evaluated *in vitro* against human colon cancer cell line Colo-205 and HCC cancer cell line HepG2. Five derivatives (**2f**, **2m**, **2k**, **2p**, and **2s**) possessed anticancer activity. Against the Colo-205 cell line, Compounds **2f** and **2m** showed the best activity profile with 50.0 and 20.5 μM IC_50_ values, respectively. Compounds **2k**, **2p**, and **2s** showed the best activity profile with 30.5, 35.9, and 20.8 μM IC_50_ values, respectively. The mechanisms of action of compounds **2f** and **2m** on the Colo-205 cell line and compounds **2k**, **2p**, and **2s** on the HepG2 cell line were detected using qRT-PCR and Western blot analyses. qRT-PCR analysis showed that there was a time-dependent increase in the expression levels of Bax and Caspase 3 on apoptosis. Inhibition of apoptotic proteins PI3K, Akt, PTEN, pPTEN, FoXO1, FoXO3a, TXNIP, and p27 was investigated in Colo-205 and HepG2 cells treated with compounds **2f**, **2m**, **2k**, **2p**, and **2s** by using Western blotting. These data demonstrated that 24 h treatment with compounds **2p** and **2s** directly up-regulated Bax expression and induced caspase-3-dependent apoptosis in the HepG2 cell line. It was determined that between **2k**, **2p**, and **2s** substances, which antitumoral activities were investigated that **2s** exert their antiproliferative effect on HepG2 cells by inhibiting PI3K and Akt levels and by inhibiting the PI3K/Akt signaling pathway by reducing the phosphorylation of PTEN. It has been recorded that compounds **2k** and **2p** exert their antiproliferative effects on HepG2 cells through the activation of the FoxO pathway by increasing FoXO1, TXNIP, and p27 protein levels.

## Supporting Information Available

Details of the spectroscopic data including ESI-HRMS, FT-IR, ^1^H NMR, and ^13^C NMR (PDF)
