# Supplementary material for: Design, Synthesis, and Anticancer Evaluation of Novel Tetracaine Hydrazide-Hydrazones
Source: ACS Omega. 2023 Feb 28;8(10):9198–211. doi: 10.1021/acsomega.2c07192 (PMC10018687; doi:10.1021/acsomega.2c07192)
Supplement: Supplementary file 1 — ao2c07192_si_001.pdf [file ao2c07192_si_001.pdf]

Design, synthesis, and anticancer evaluation of novel Tetracaine hydrazide-hydrazones

M.İhsan Han[a*], Nalan İmamoğlu[b]

[a] Department of Pharmaceutical Chemistry, Faculty of Pharmacy, Erciyes University, 38039, Kayseri, Turkey

[b] Department of Basic Sciences, Faculty of Pharmacy, Erciyes University, 38039, Kayseri, Turkey

[*]corresponding author: Department of Pharmaceutical Chemistry, Faculty of Pharmacy, Erciyes University, 38039, Kayseri, Turkey

e-mail: hanihsan@gmail.com

Contents:

1- FT-IR, ^{1}H-NMR, ^{13}C-NMR and HR-MS spectra of novel compounds (**2a-t**)

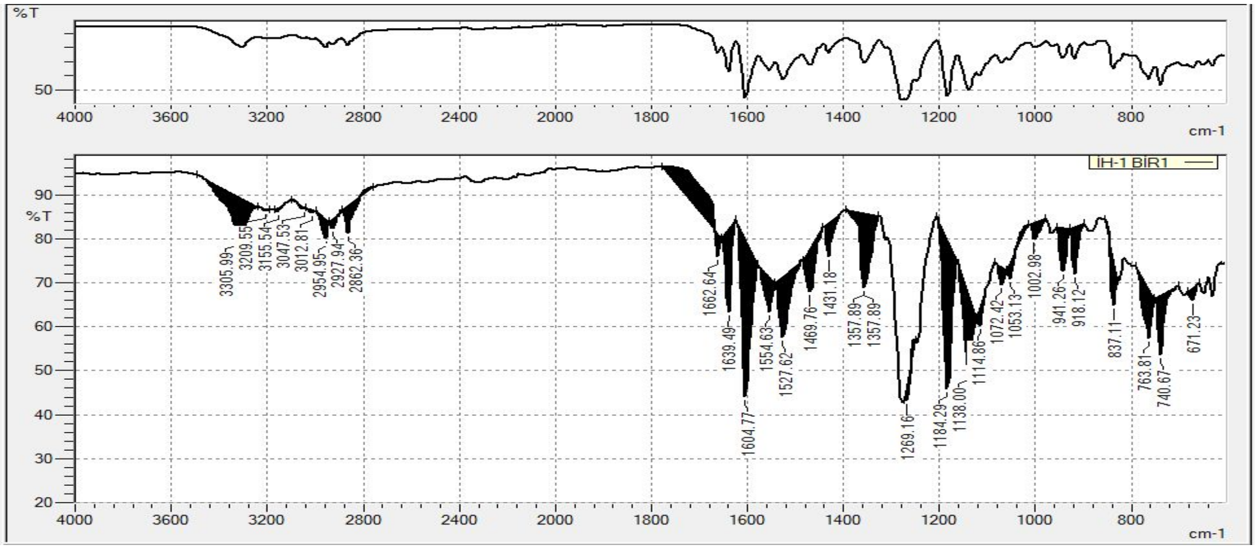

Figure S1. FT-IR spectrum of compound **2a**

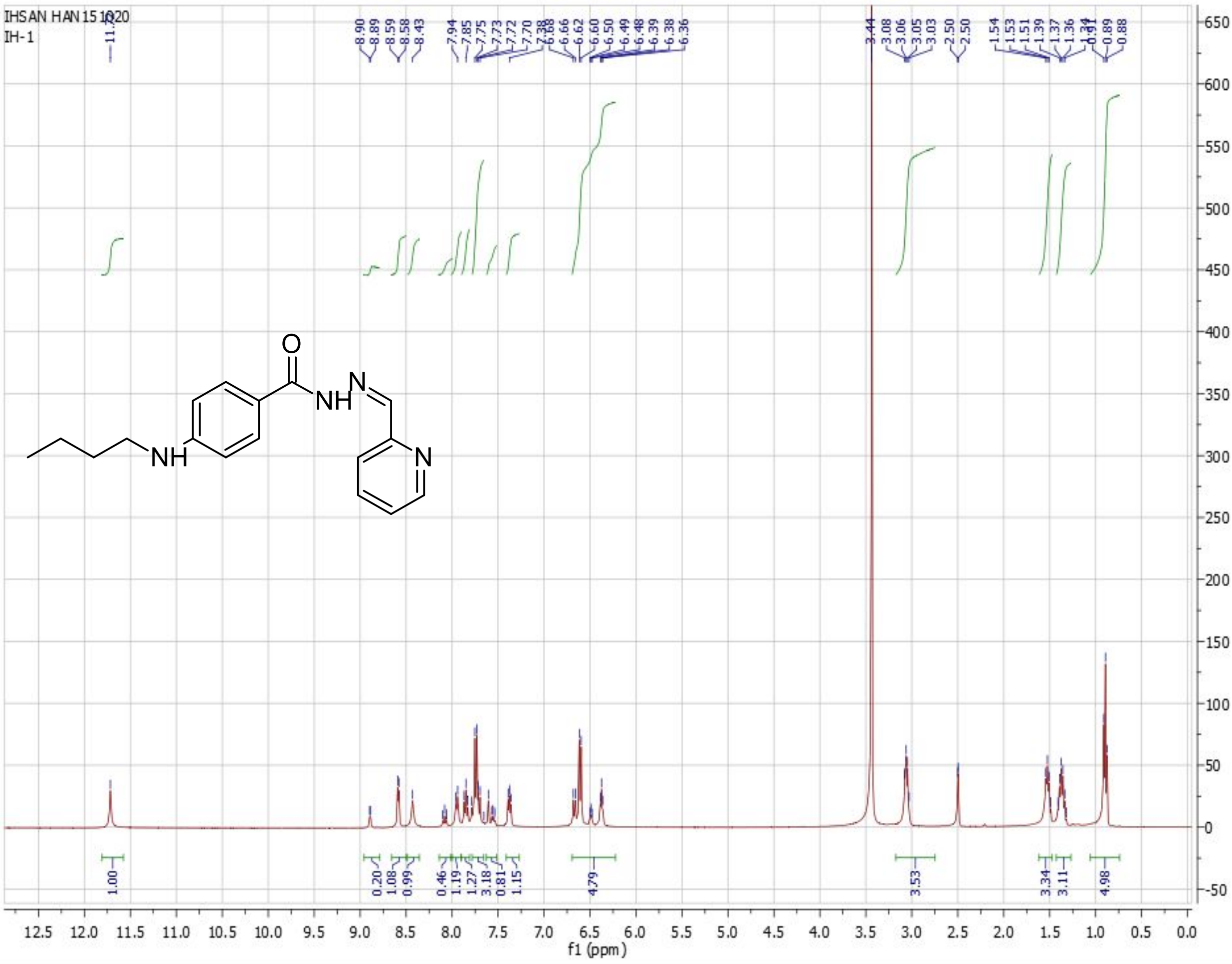

Figure S2. ^{1}H-NMR Spectra of compound **2a**

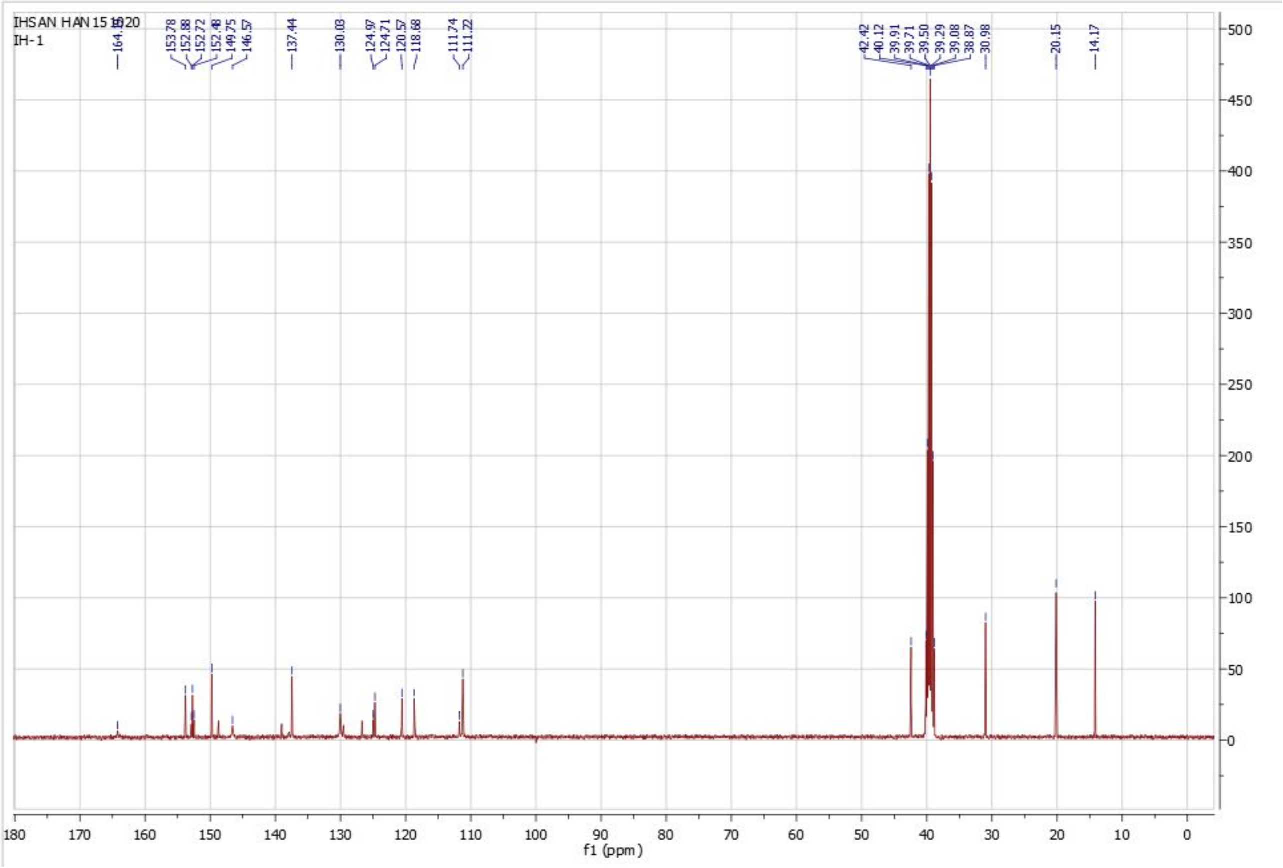

Figure S3. ^{13}C-NMR Spectra of compound **2a**

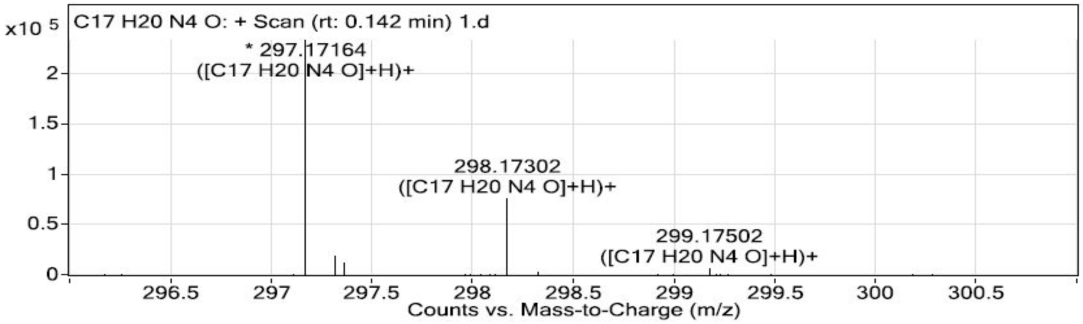

Figure S4. HR-Mass spectra of compound **2a**

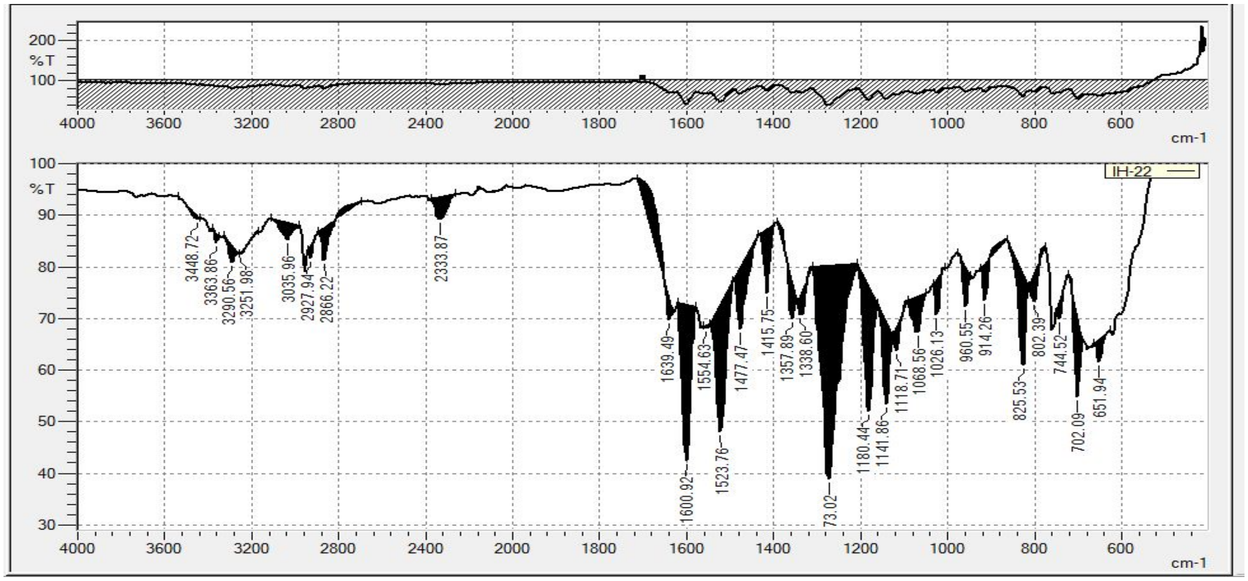

Figure S5. FT-IR spectrum of compound **2b**

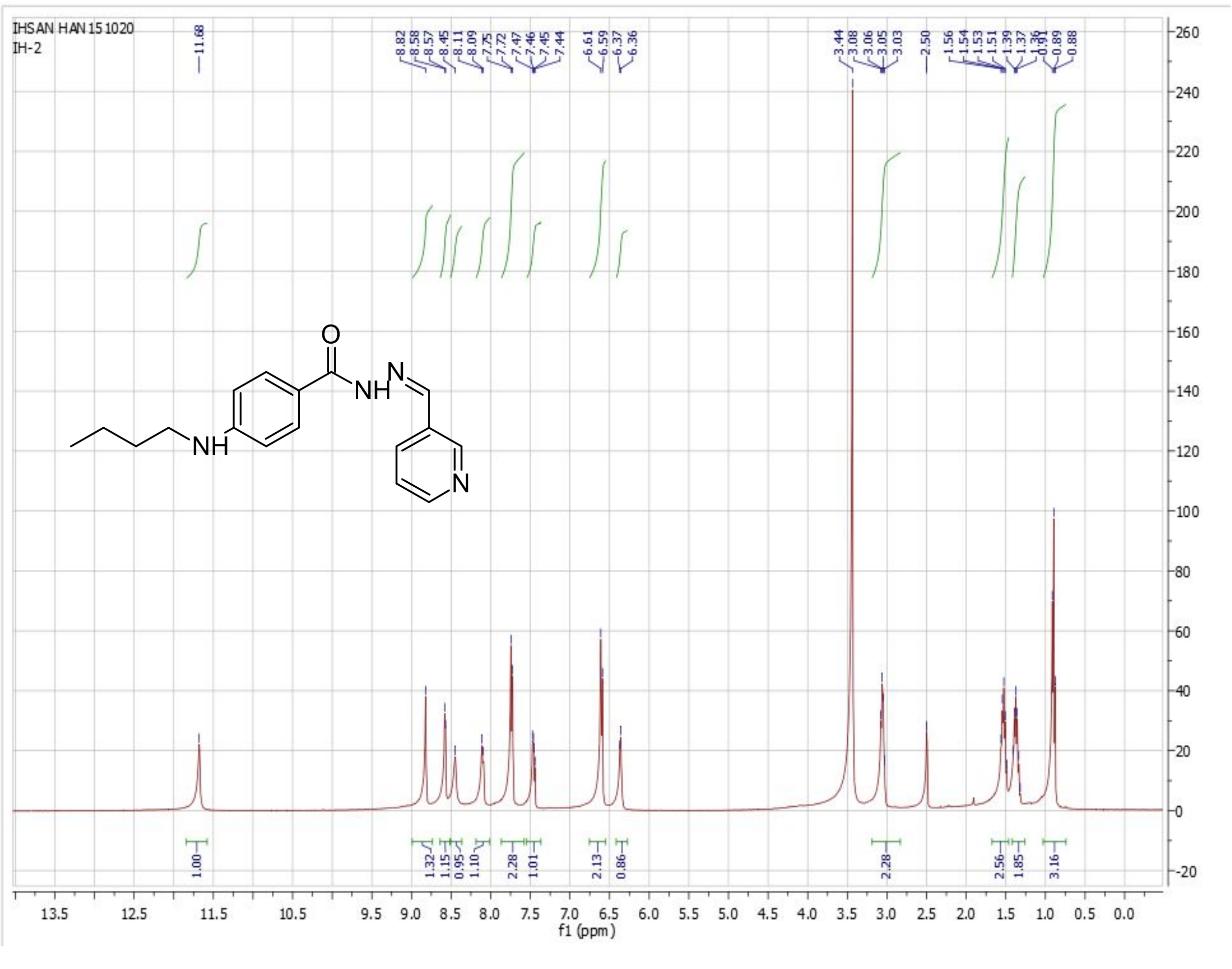

Figure S6. ^{1}H-NMR Spectra of compound **2b**

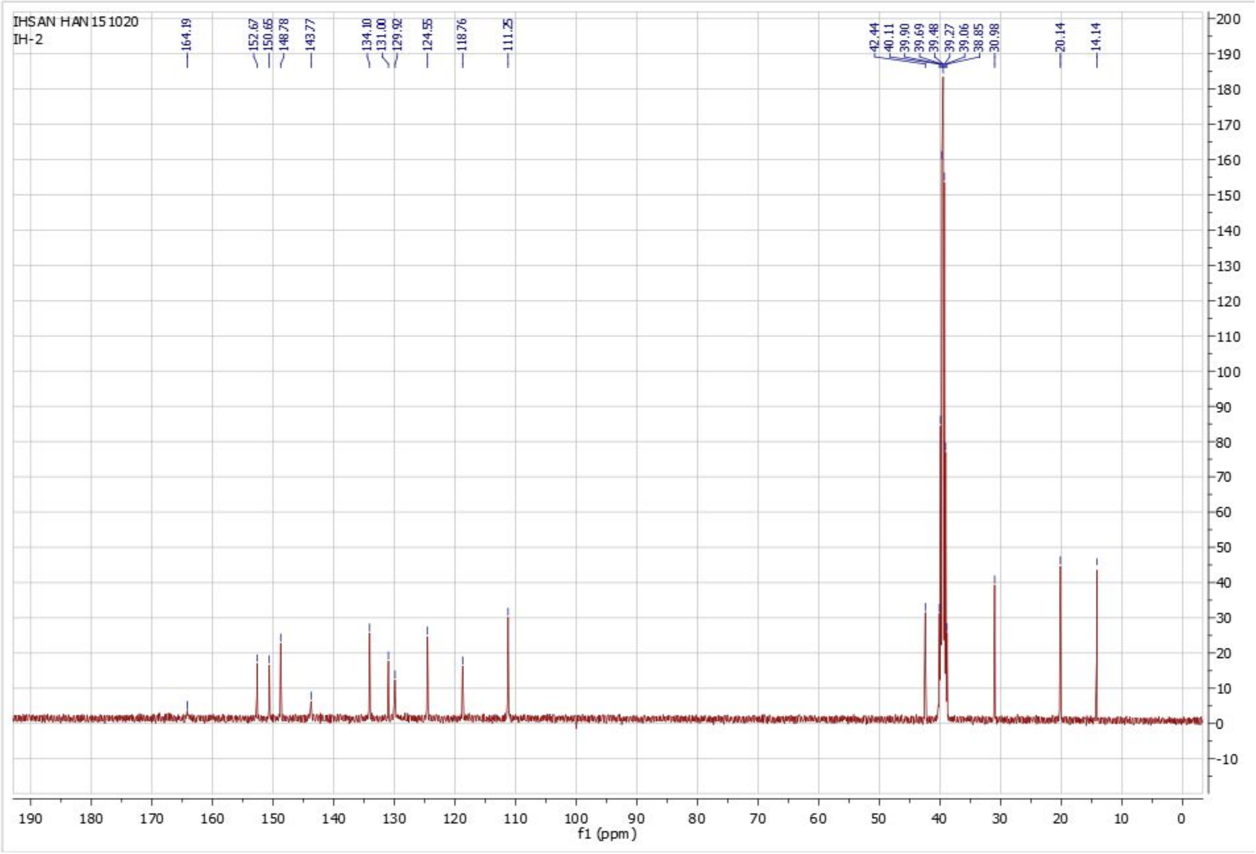

Figure S7. ^{13}C-NMR Spectra of compound **2b**

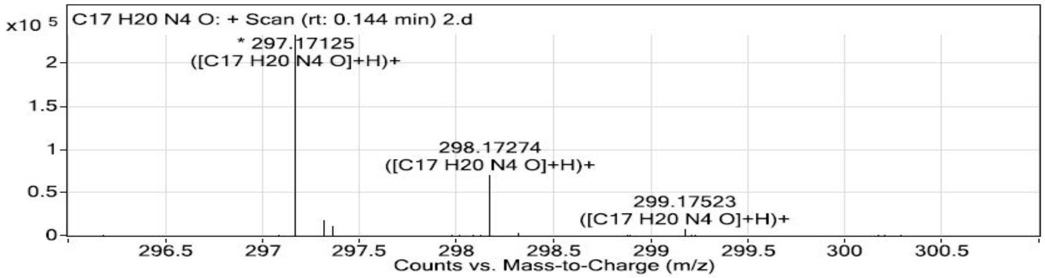

Figure S8. HR-Mass spectra of compound **2b**

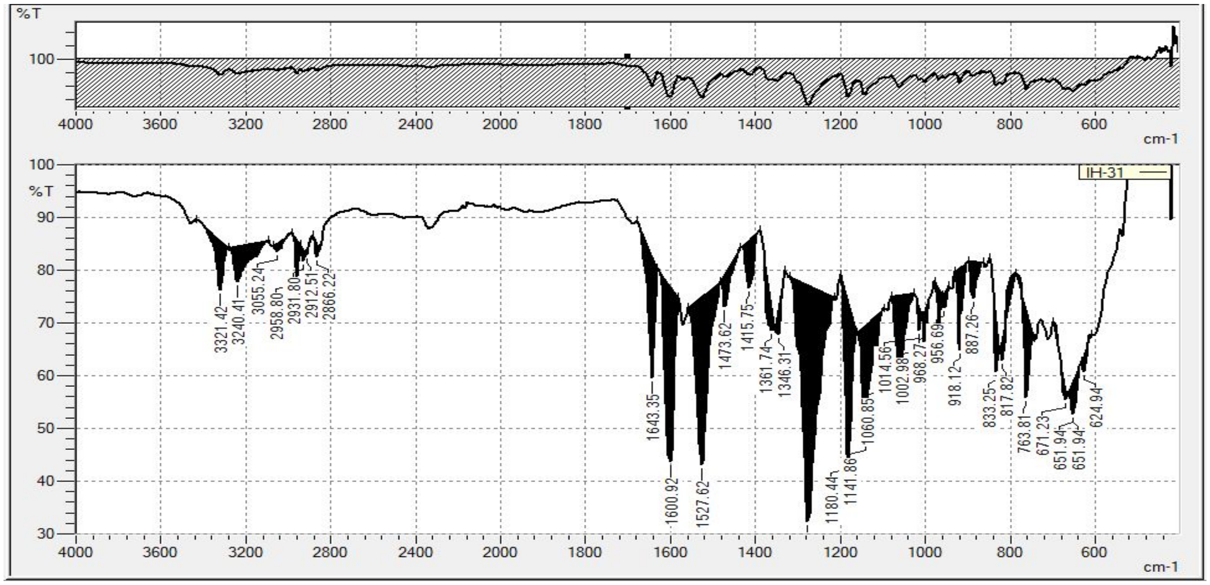

Figure S9. FT-IR spectrum of compound **2c**

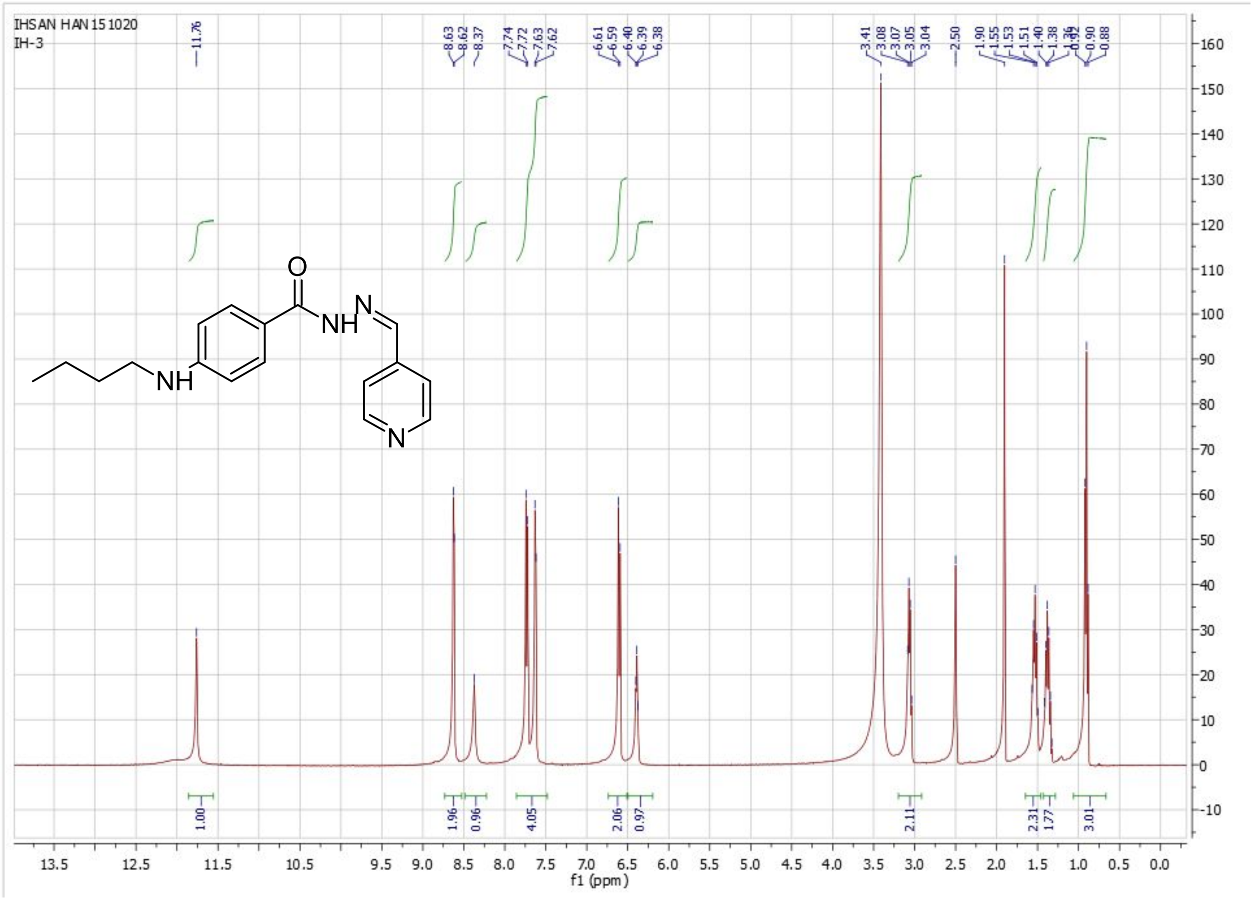

Figure S10. ^{1}H-NMR Spectra of compound **2c**

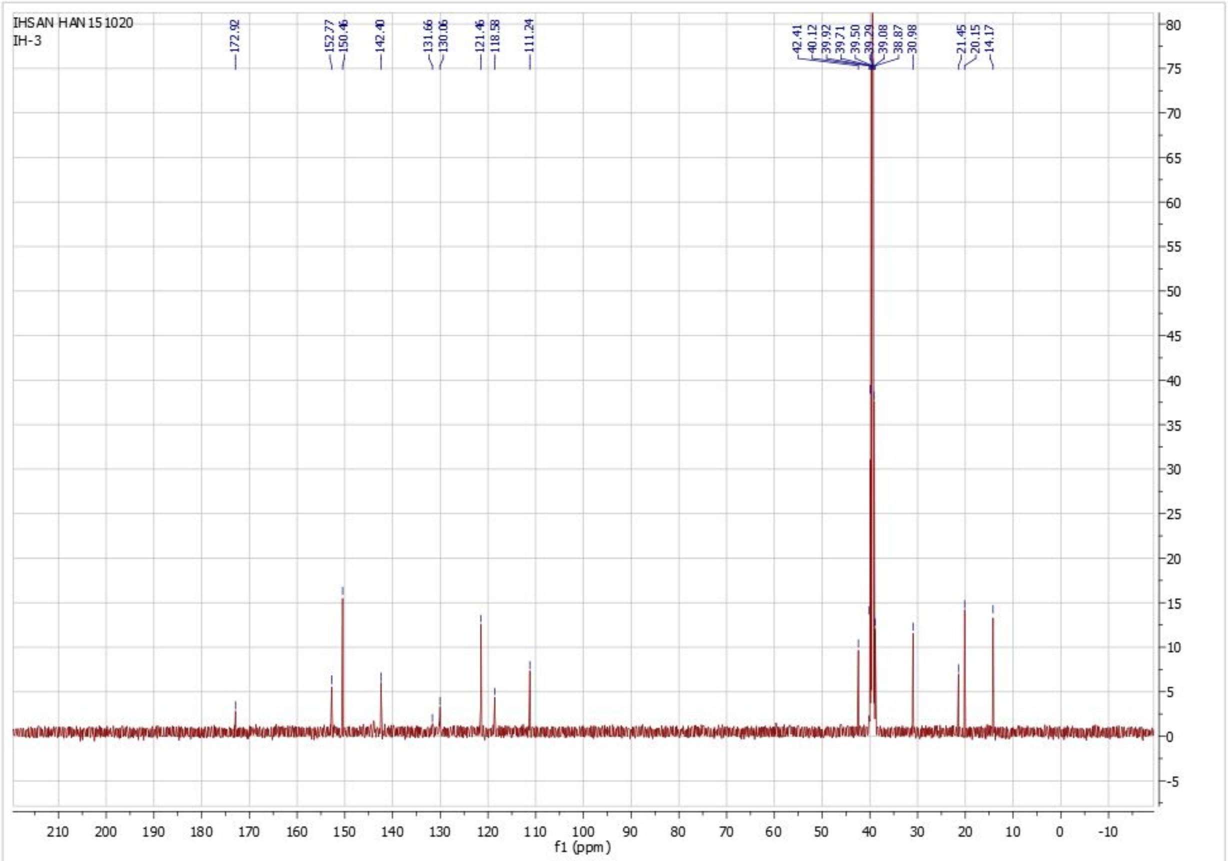

Figure S11. ^{13}C-NMR Spectra of compound **2c**

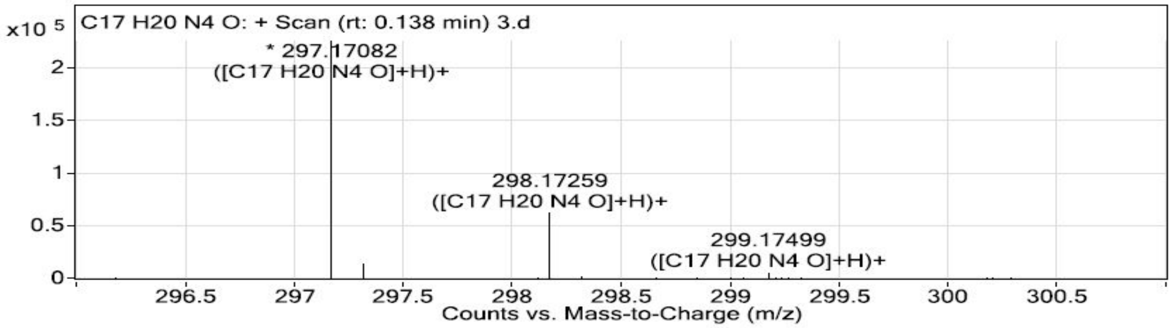

Figure S12. HR-Mass spectra of compound **2c**

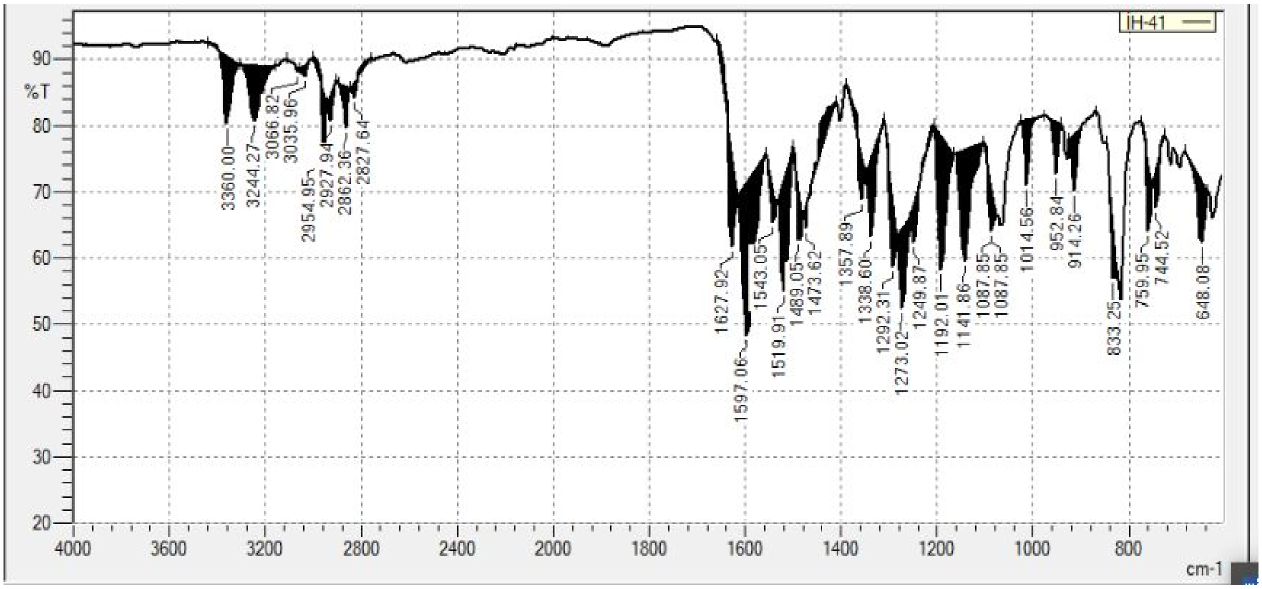

Figure S13. FT-IR spectrum of compound **2d**

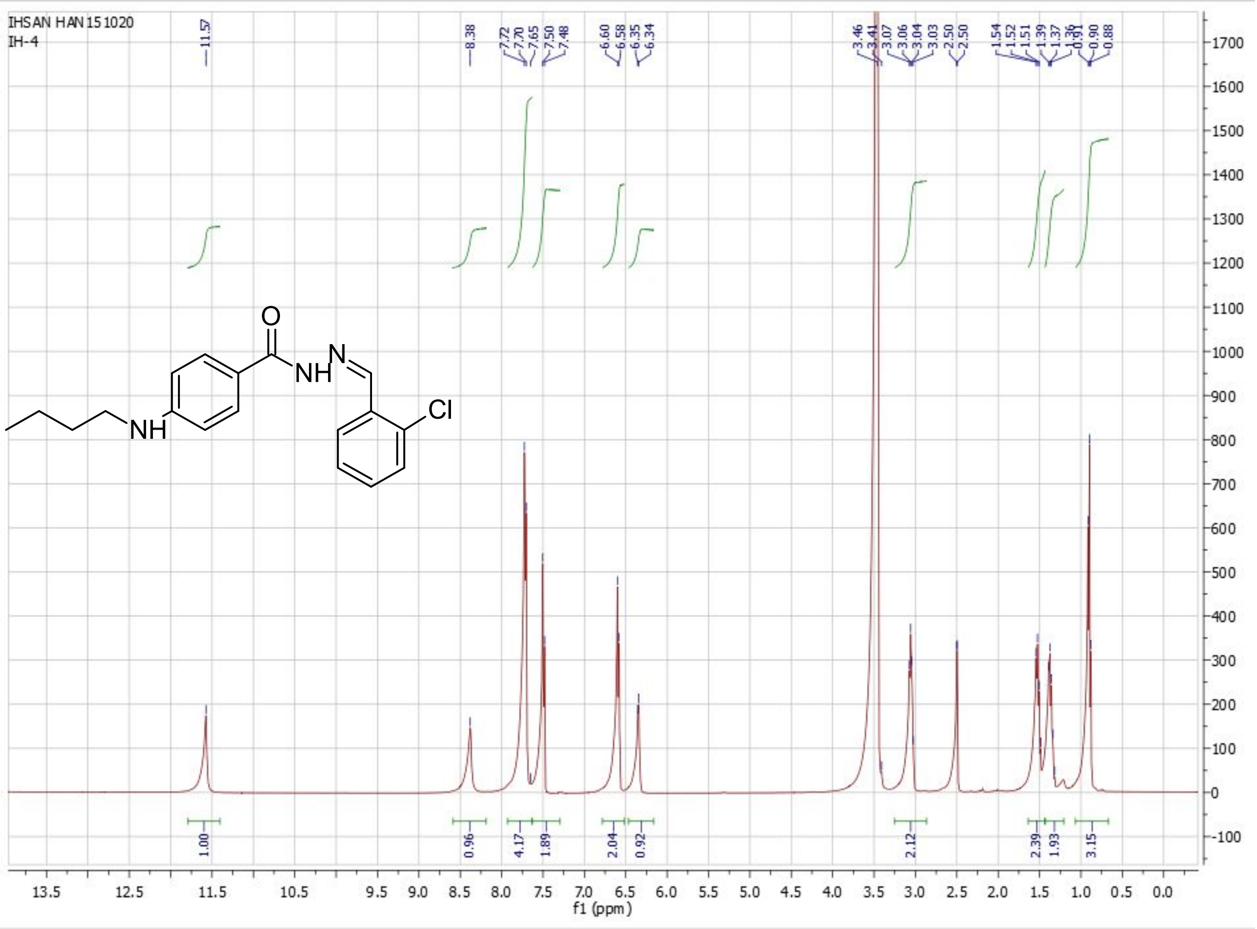

Figure S14. ^{1}H-NMR Spectra of compound **2d**

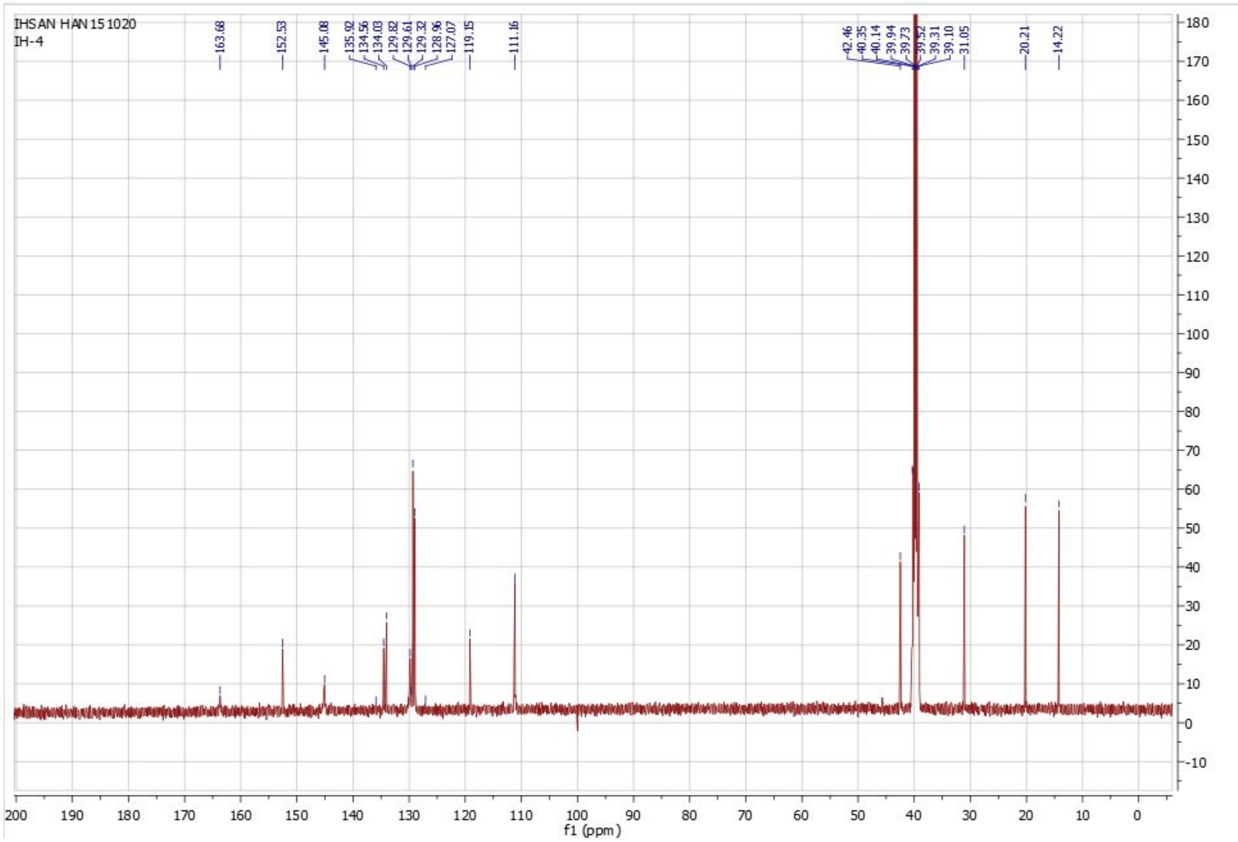

Figure S15. ^{13}C-NMR Spectra of compound **2d**

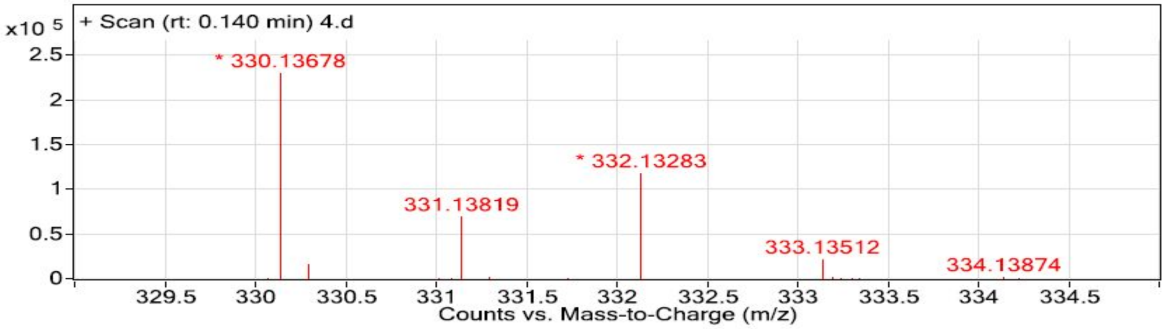

Figure S16. HR-Mass spectra of compound **2d**

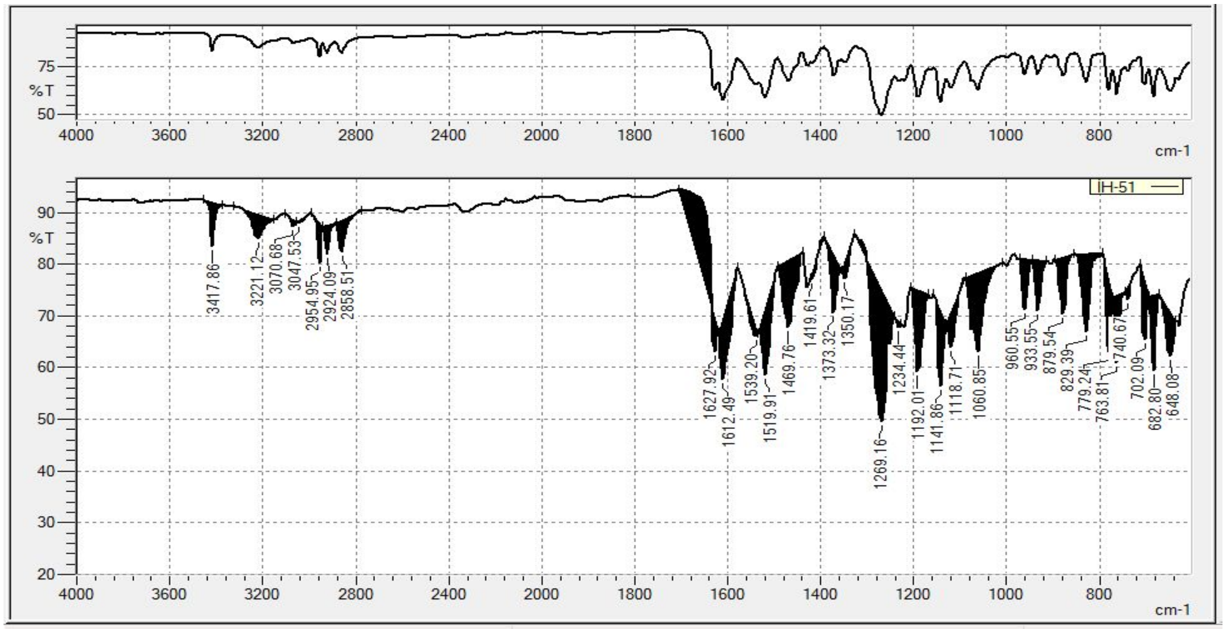

Figure S17. FT-IR spectrum of compound **2e**

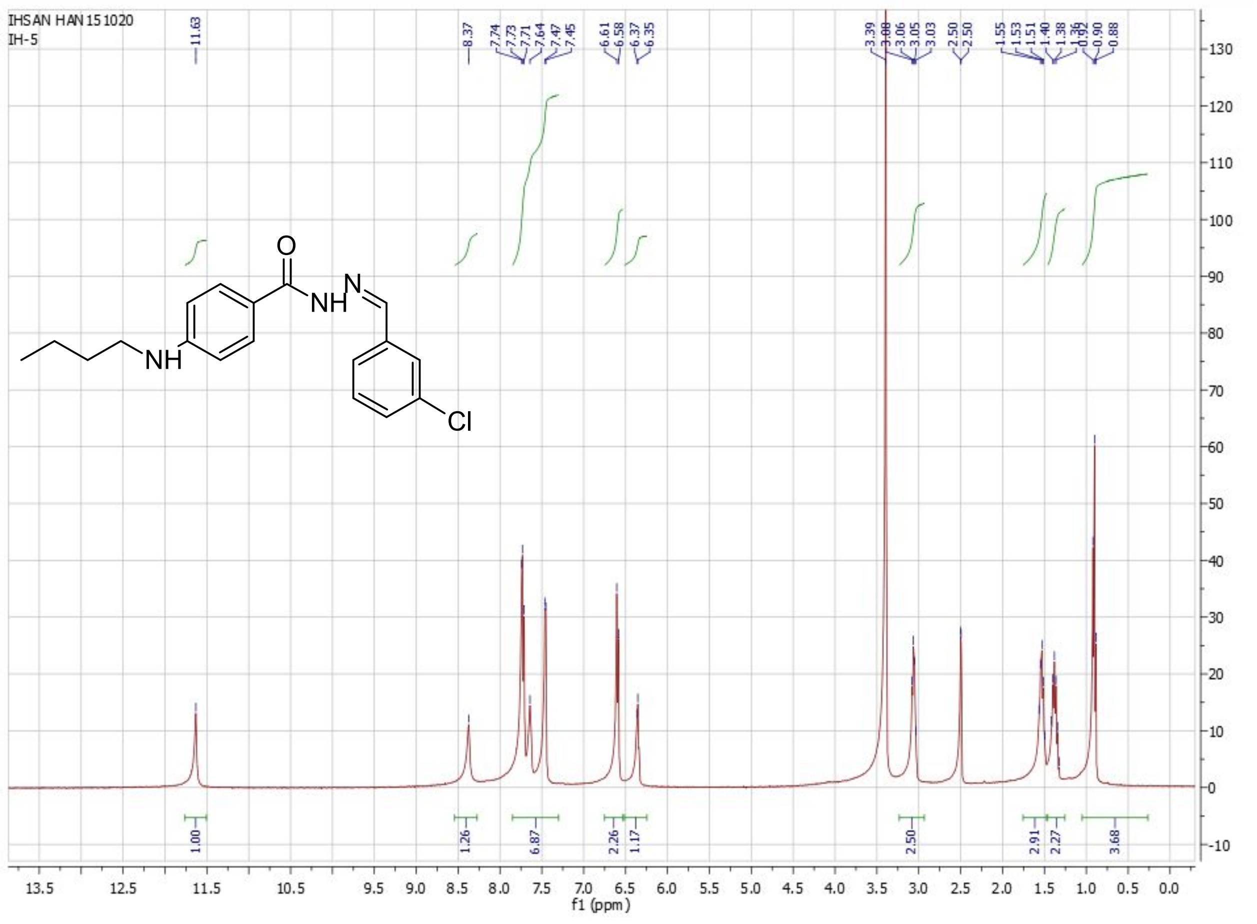

Figure S18. ^{1}H-NMR Spectra of compound **2e**

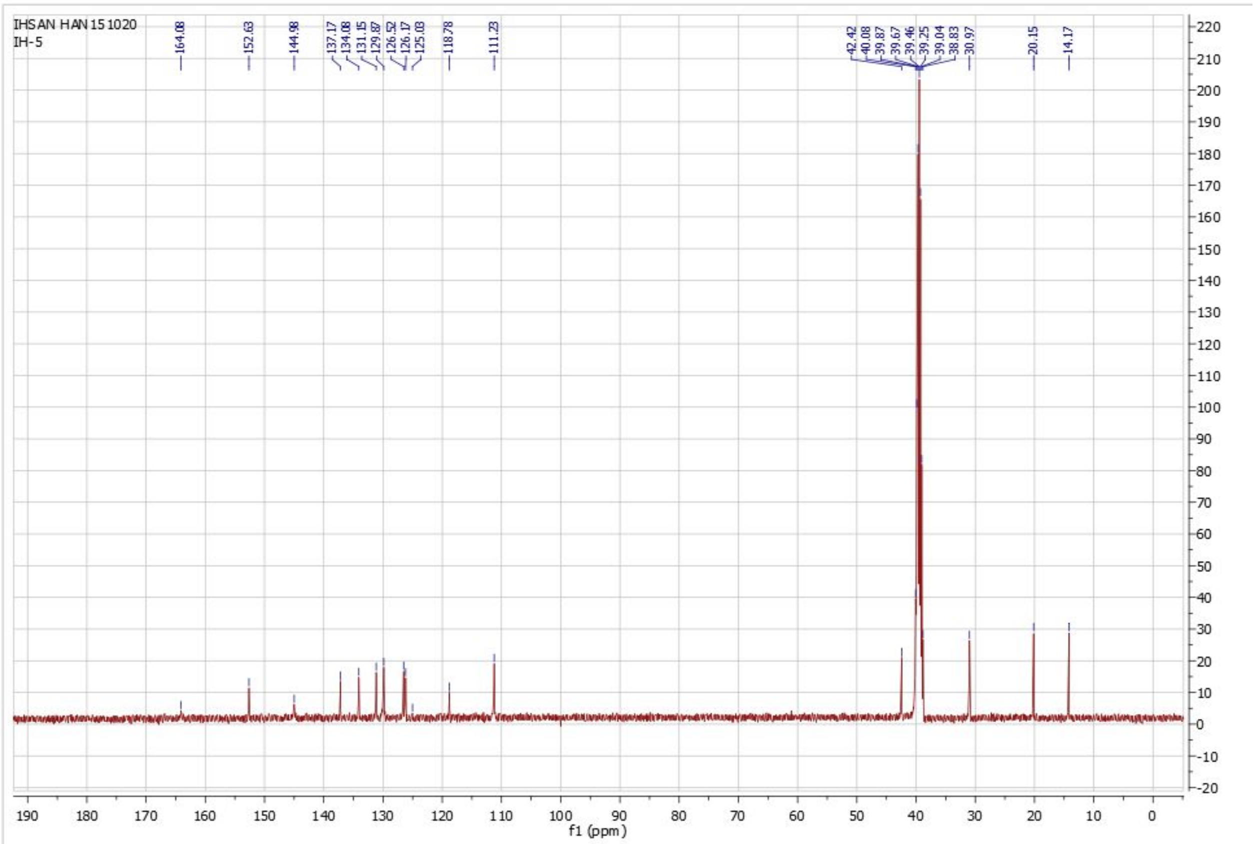

Figure S19. ^{13}C-NMR Spectra of compound **2e**

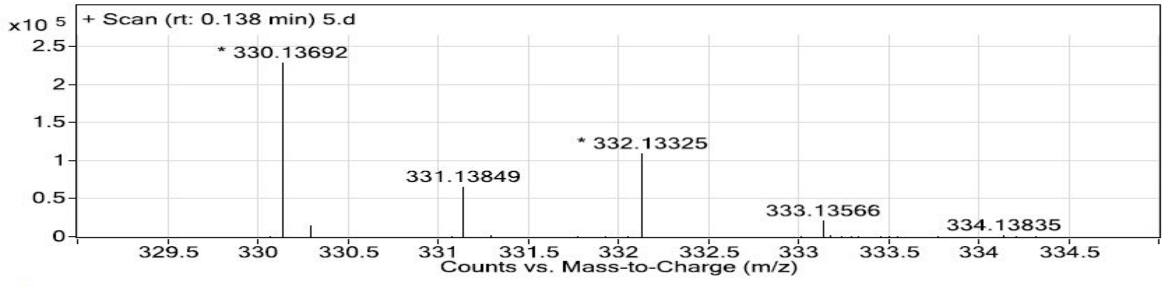

Figure S20. HR-Mass spectra of compound **2e**

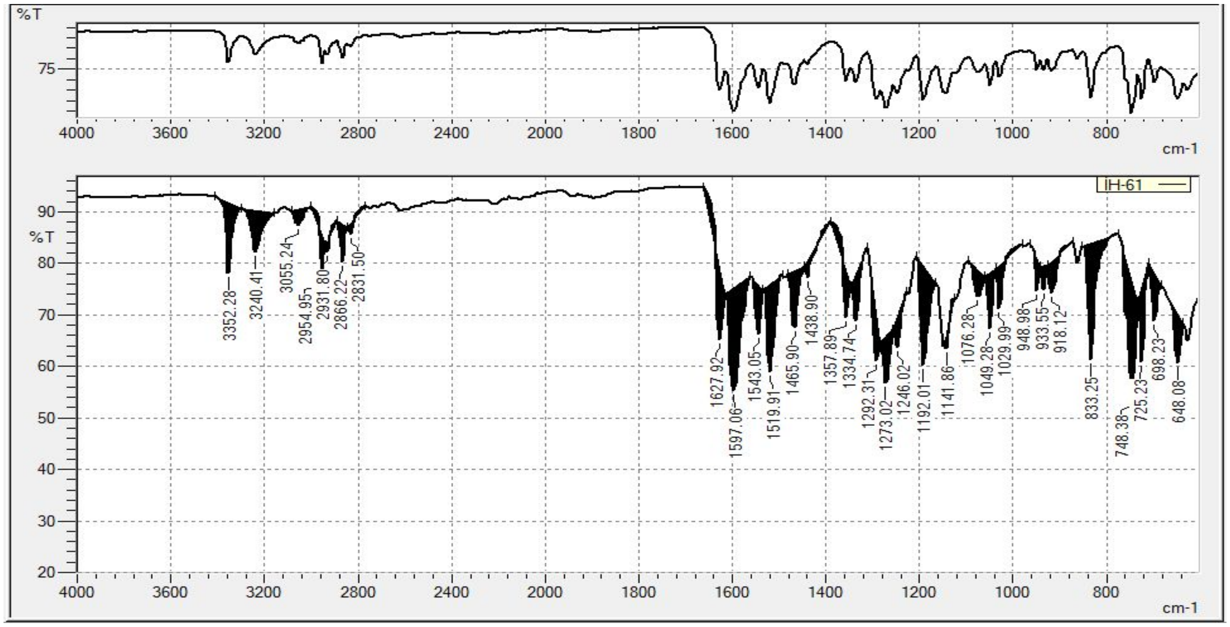

Figure S21. FT-IR spectrum of compound **2f**

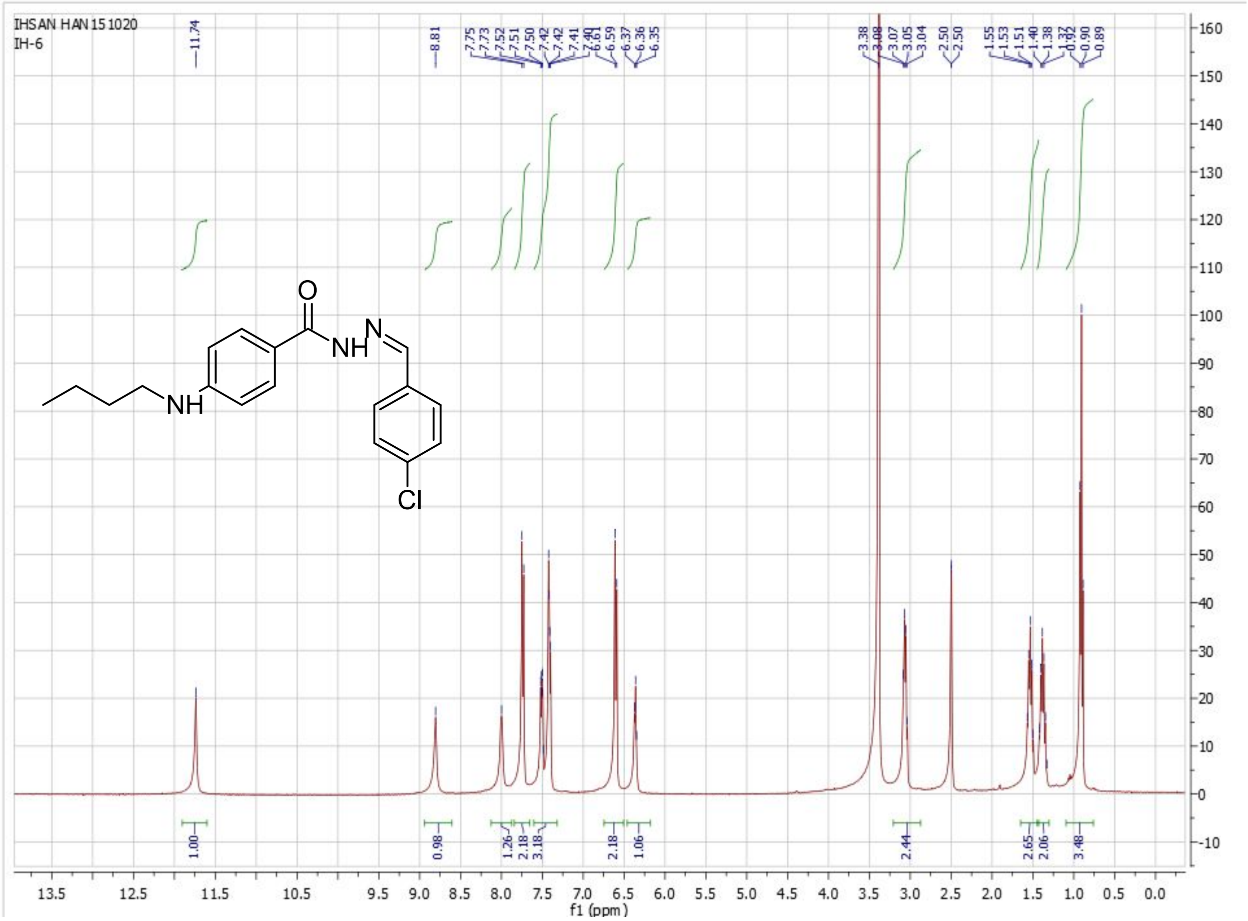

Figure S22. ^{1}H-NMR Spectra of compound **2f**

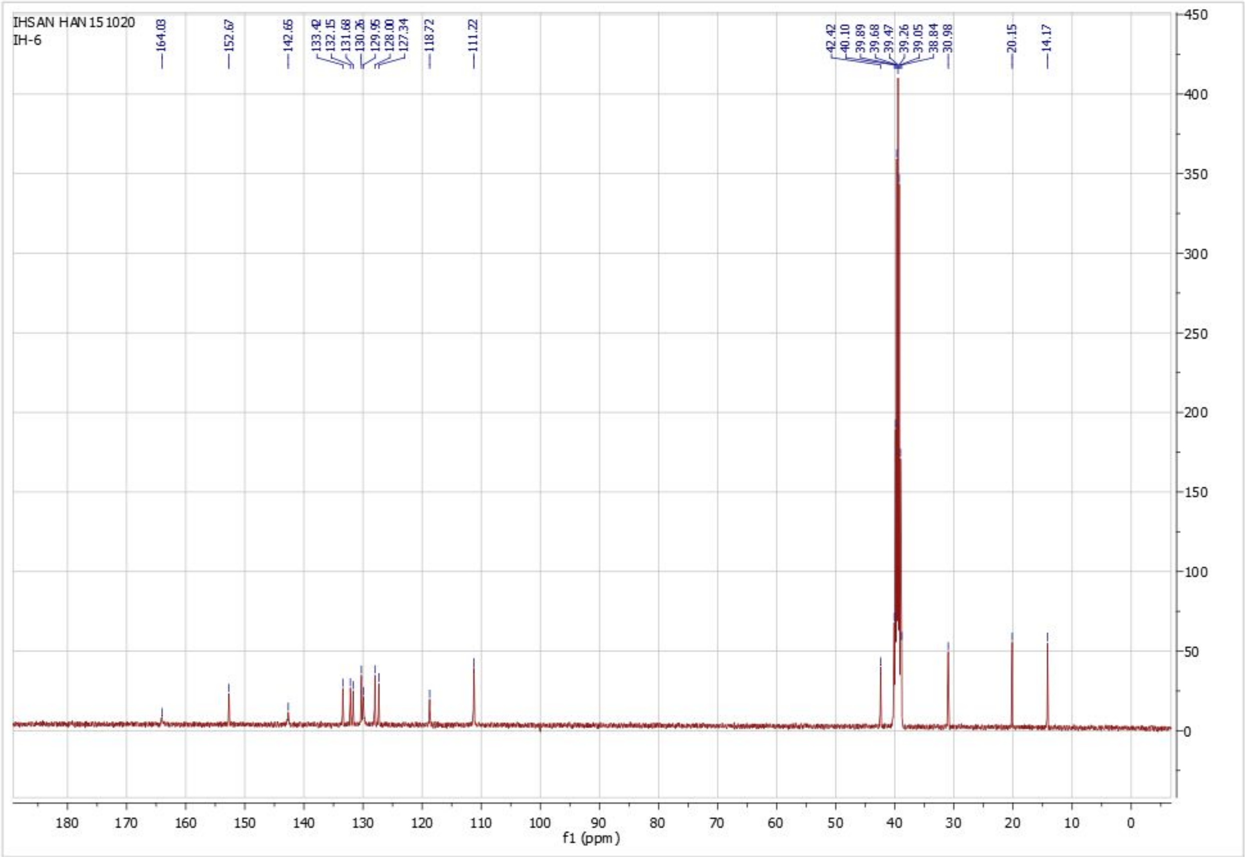

Figure S23. ^{13}C-NMR Spectra of compound **2f**

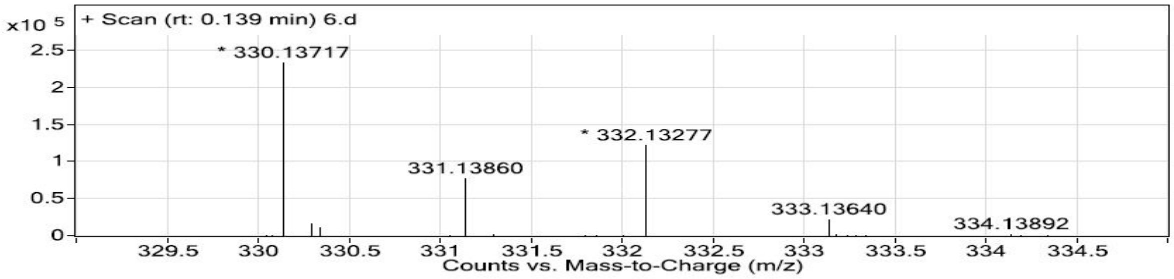

Figure S24. HR-Mass spectra of compound **2f**

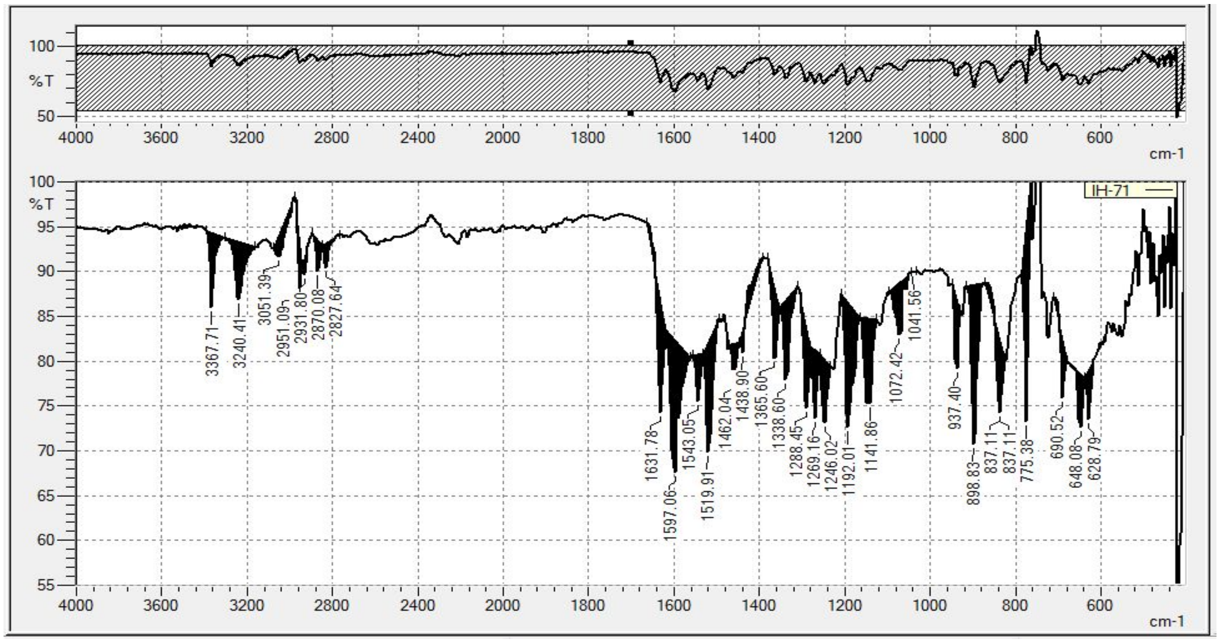

Figure S25. FT-IR spectrum of compound **2g**

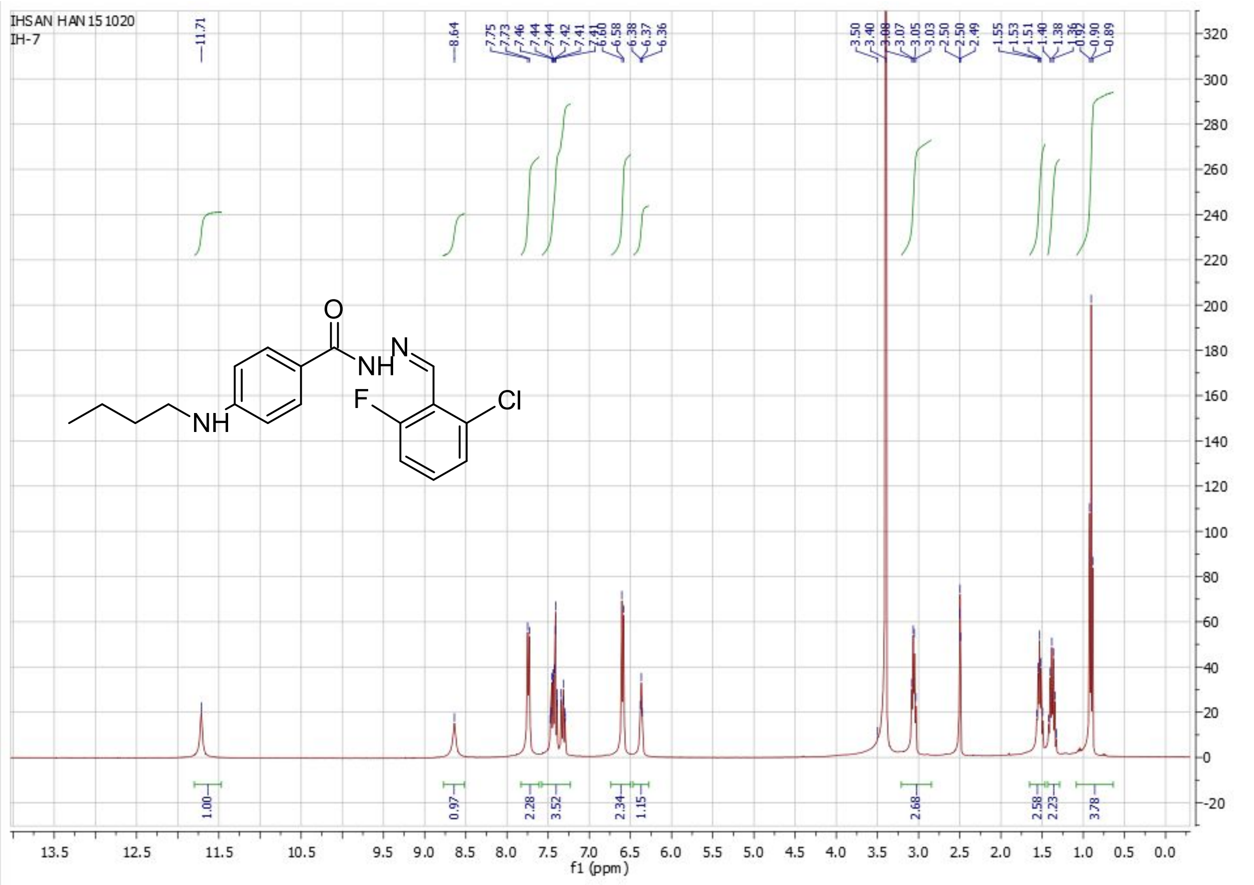

Figure S26. ^{1}H-NMR Spectra of compound **2g**

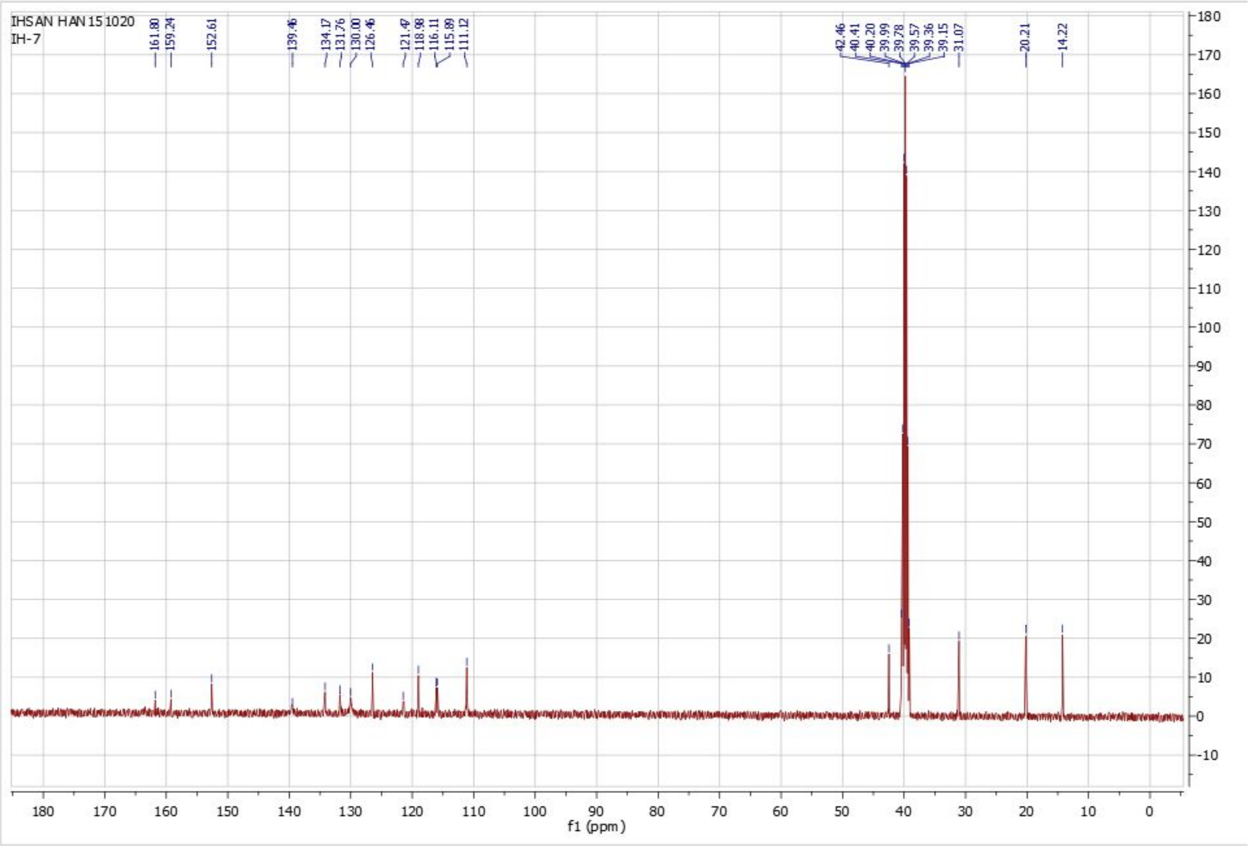

Figure S27. ^{13}C-NMR Spectra of compound **2g**

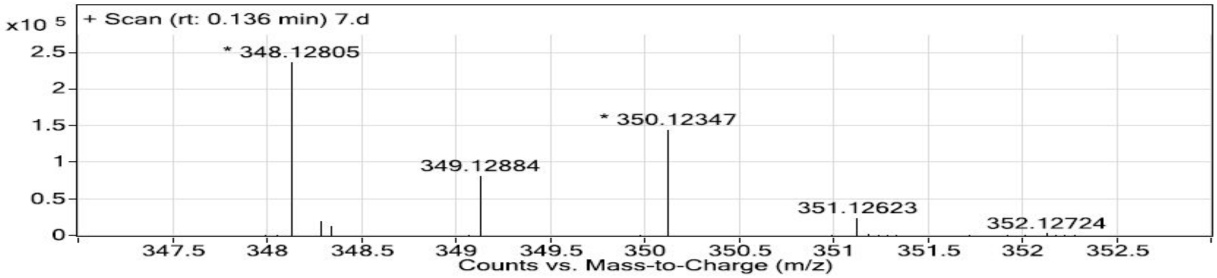

Figure S28. HR-Mass spectra of compound **2g**

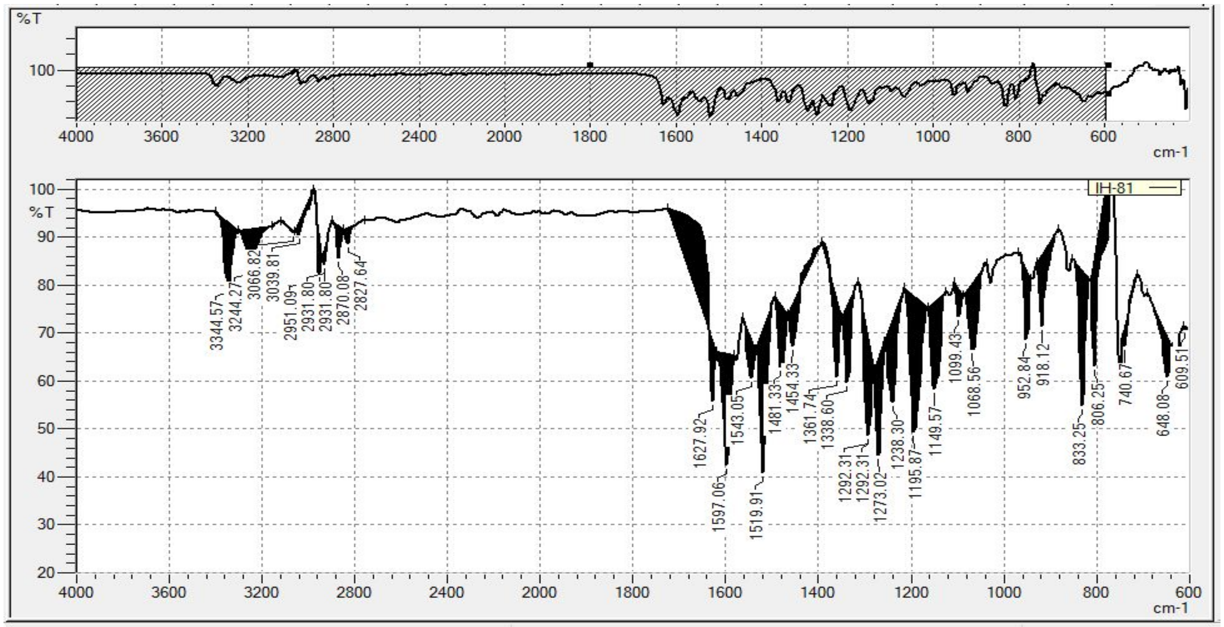

Figure S29. FT-IR spectrum of compound **2h**

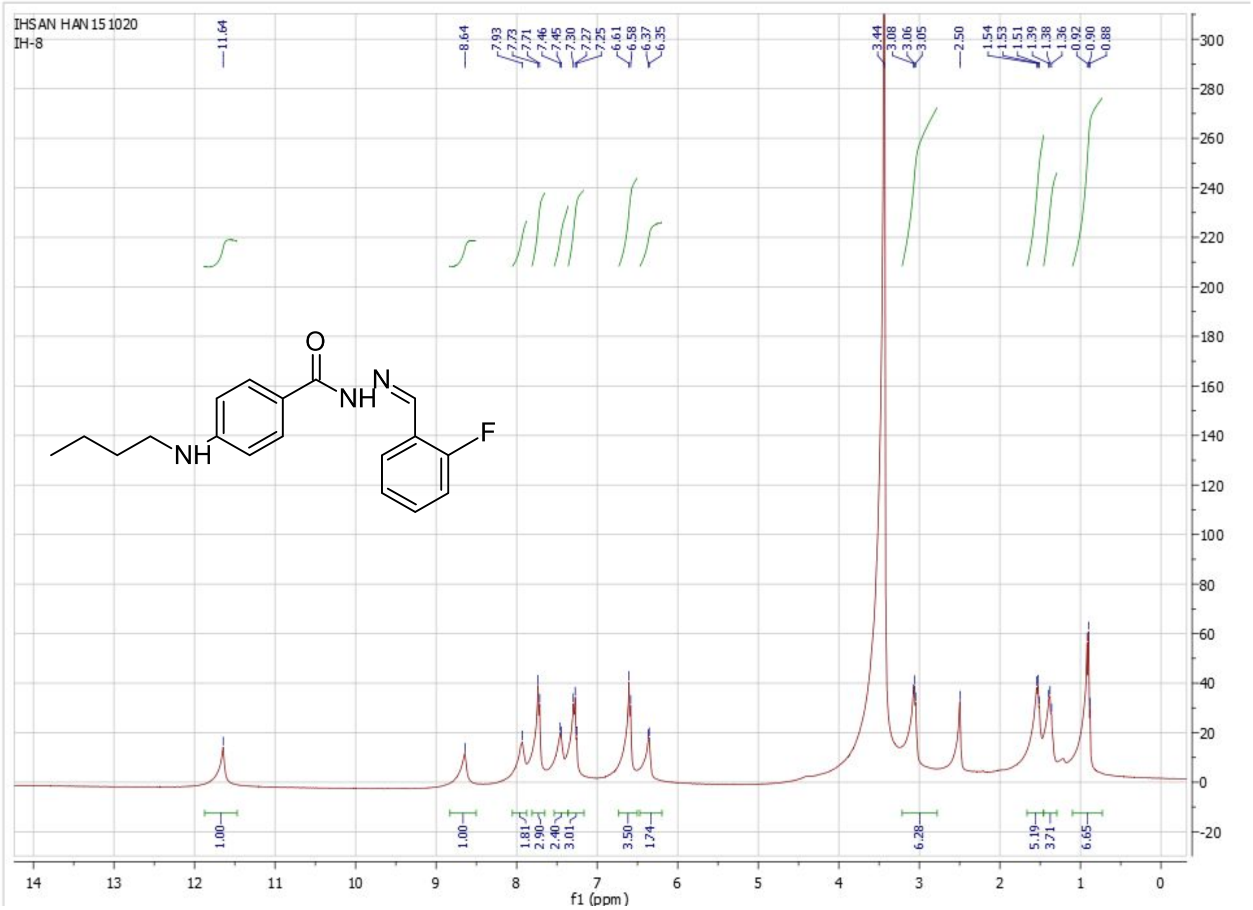

Figure S30. ^{1}H-NMR Spectra of compound **2h**

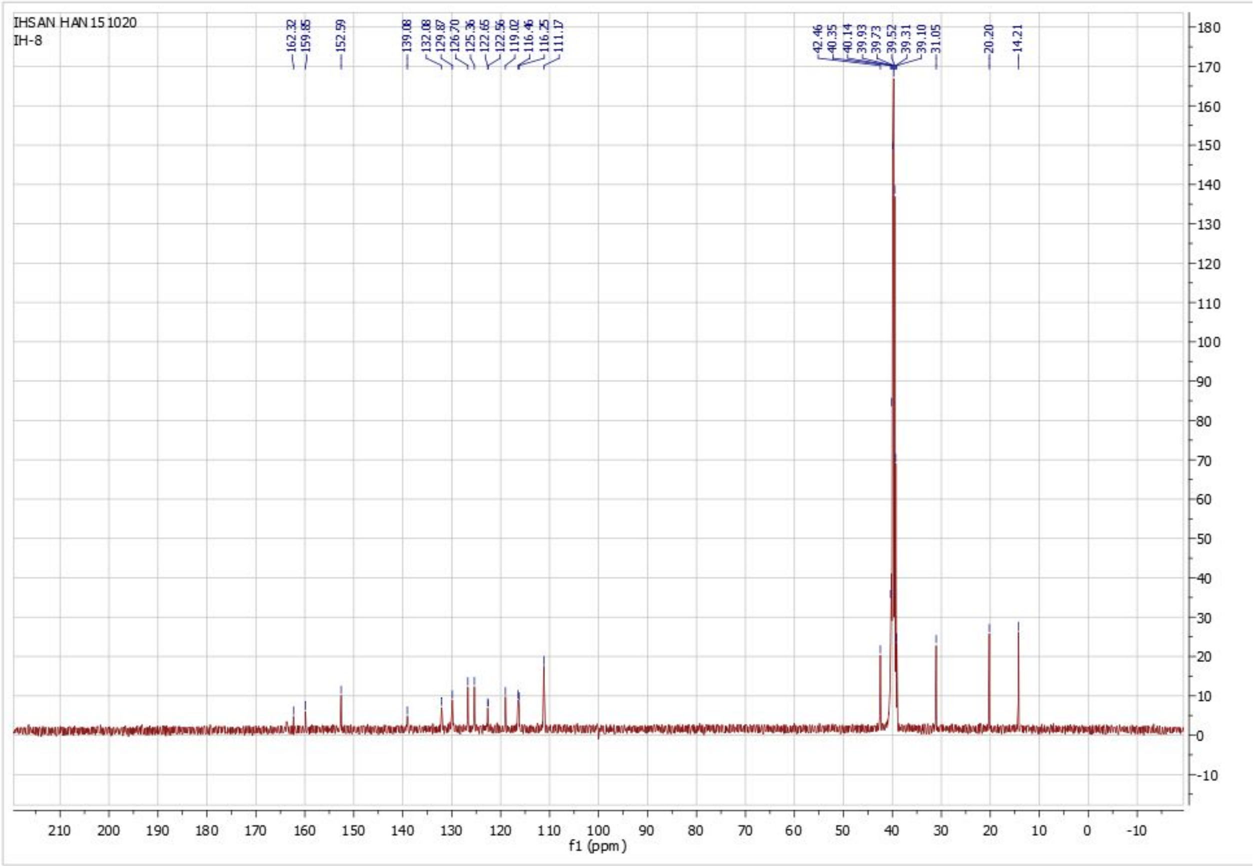

Figure S31. ^{13}C-NMR Spectra of compound **2h**

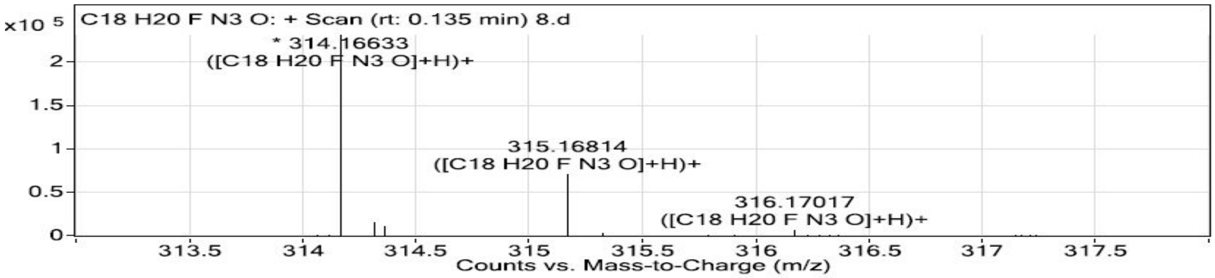

Figure S32. HR-Mass spectra of compound **2h**

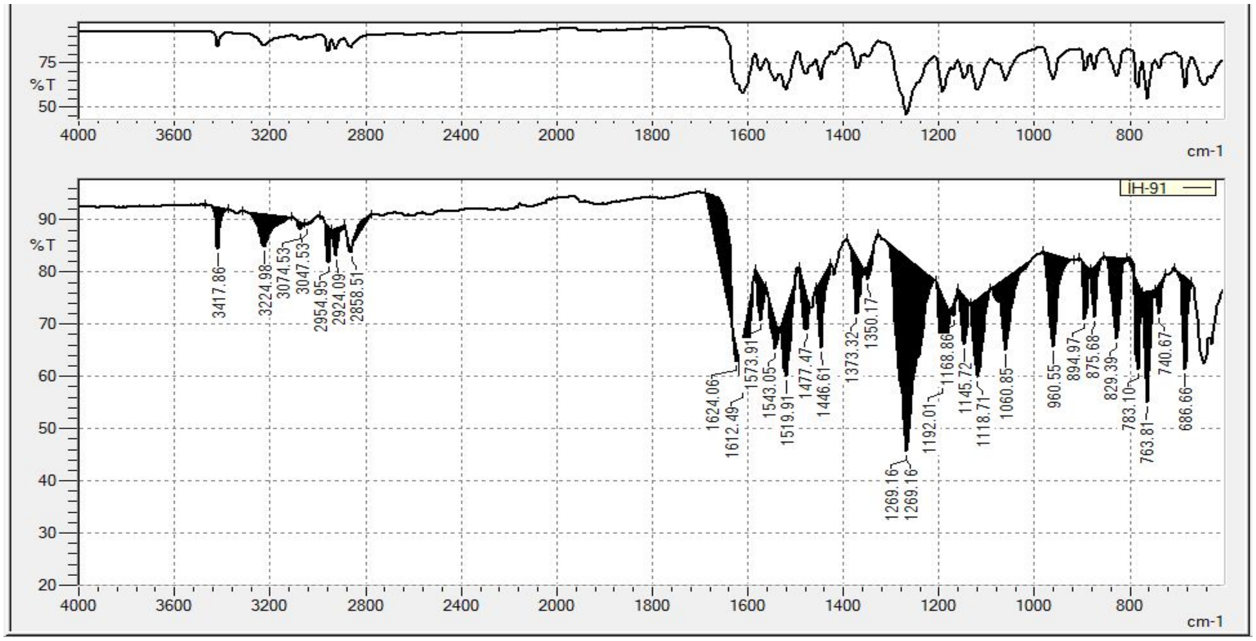

Figure S33. FT-IR spectrum of compound **2i**

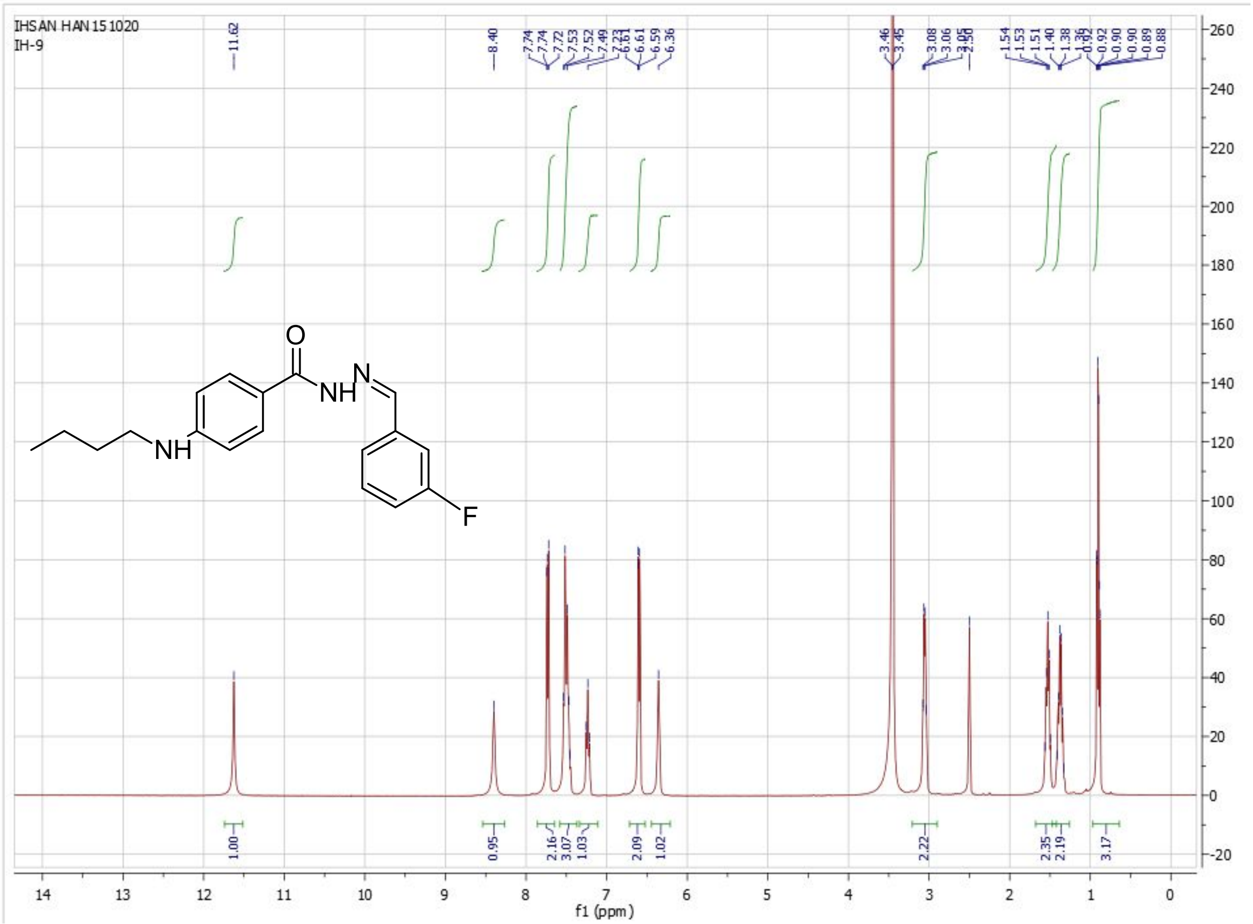

Figure S34. ^{1}H-NMR Spectra of compound **2i**

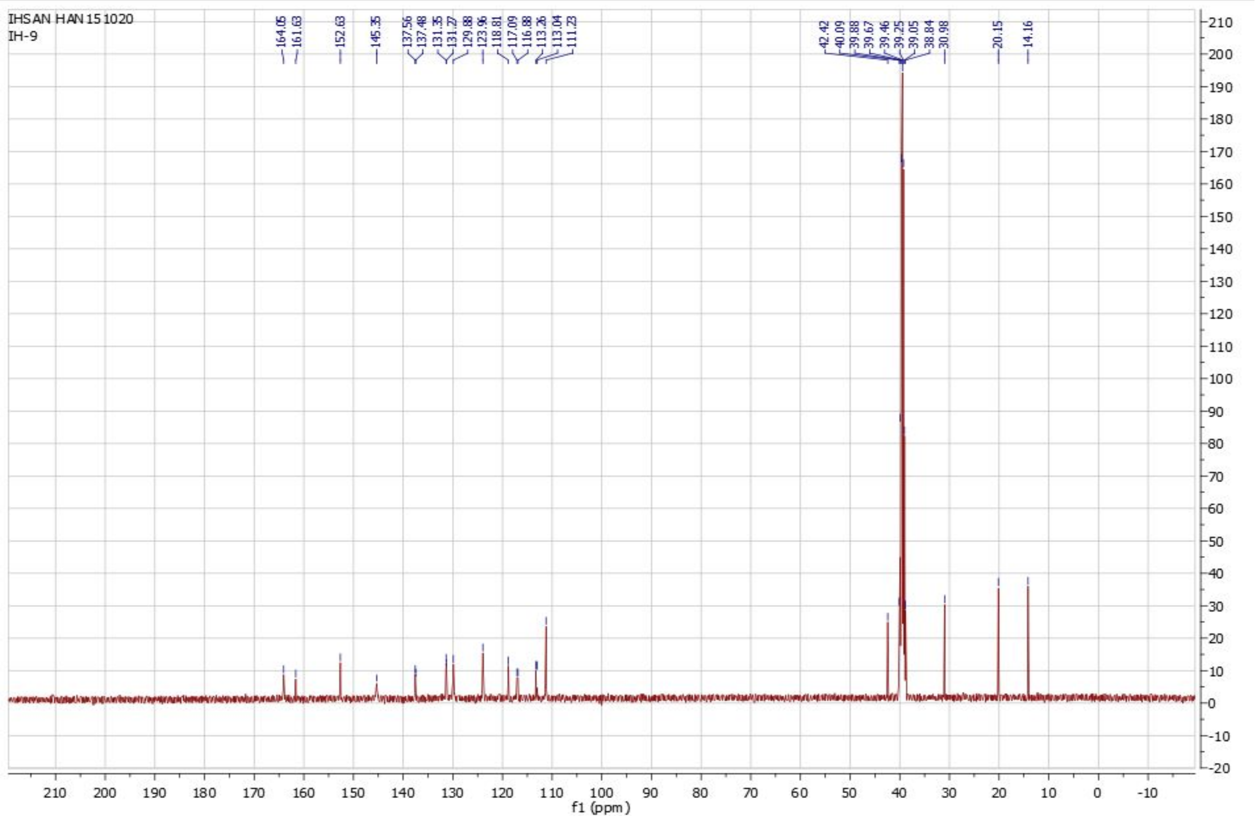

Figure S35. ^{13}C-NMR Spectra of compound **2i**

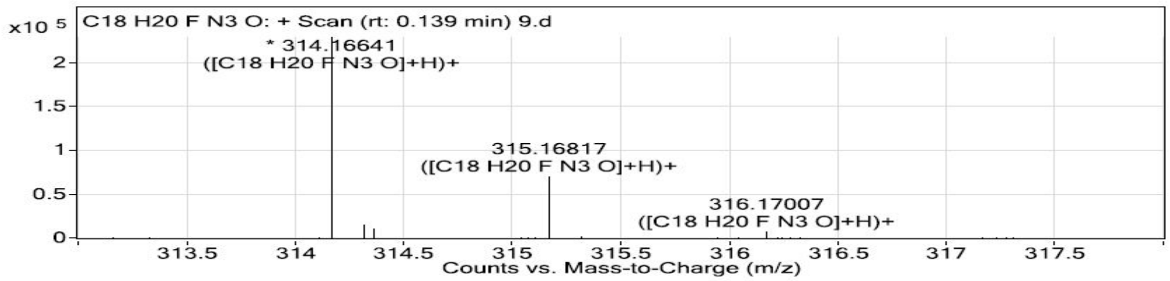

Figure S36. HR-Mass spectra of compound **2i**

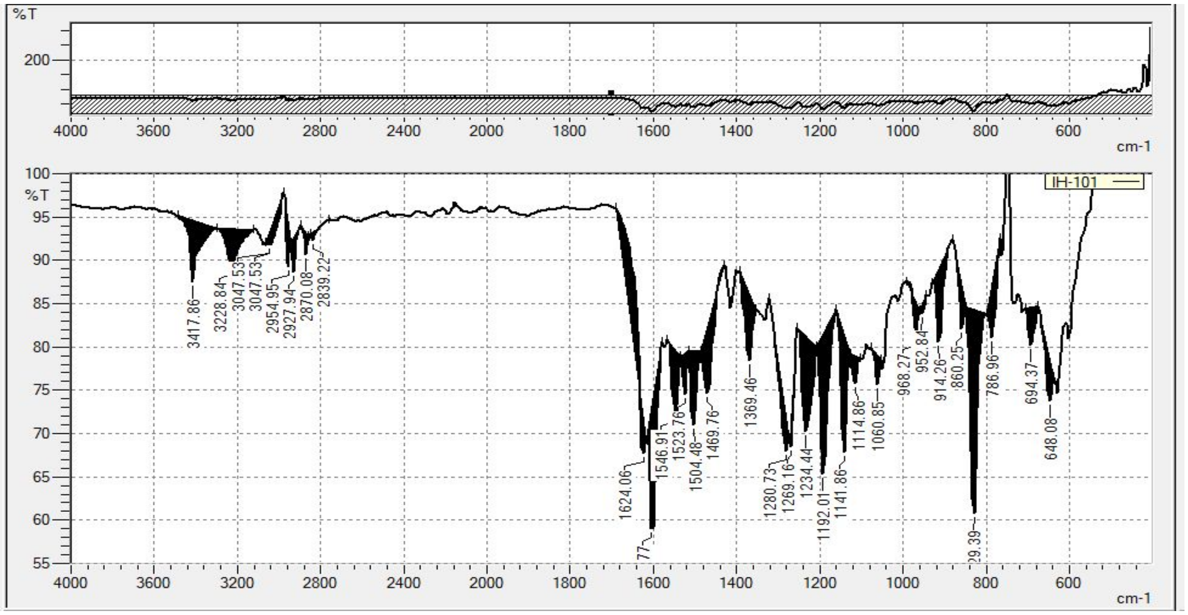

Figure S37. FT-IR spectrum of compound **2j**

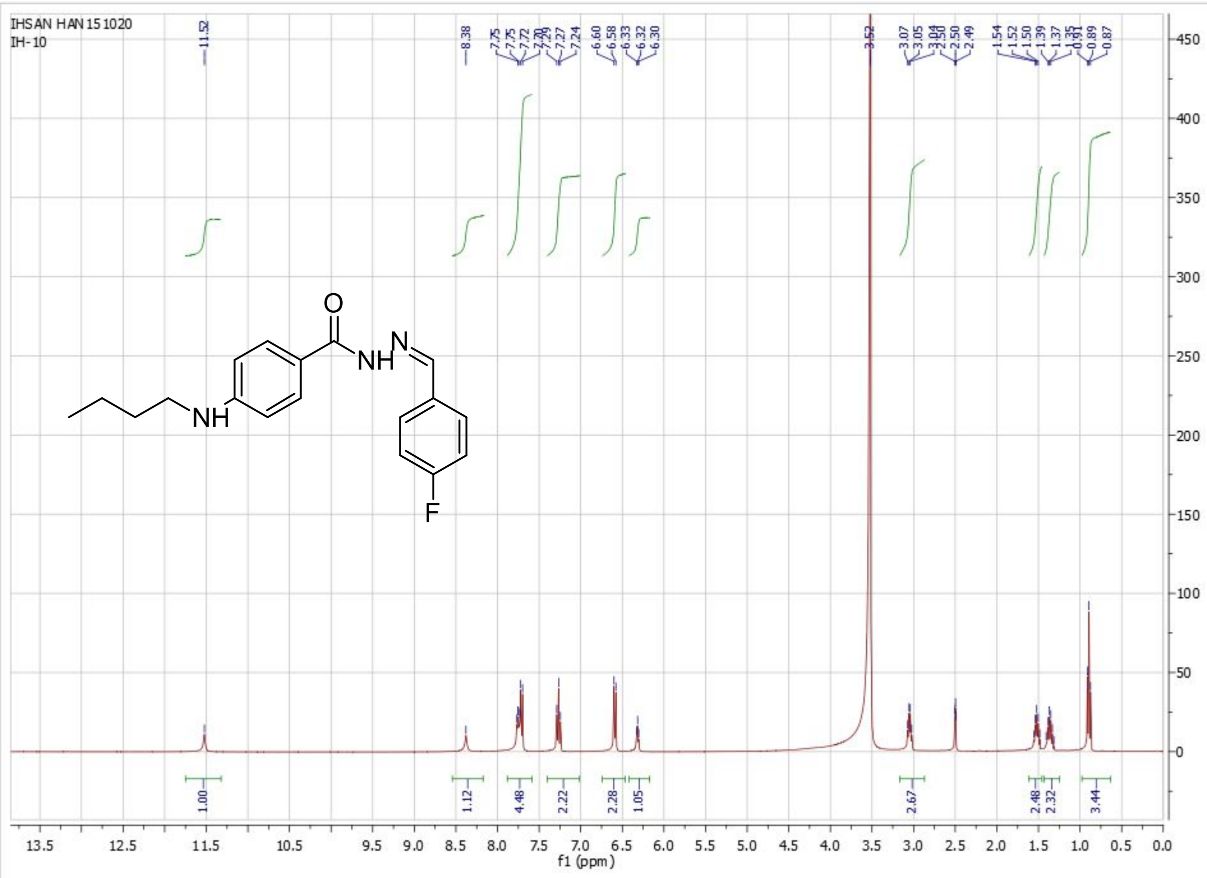

Figure S38. ^{1}H-NMR Spectra of compound **2j**

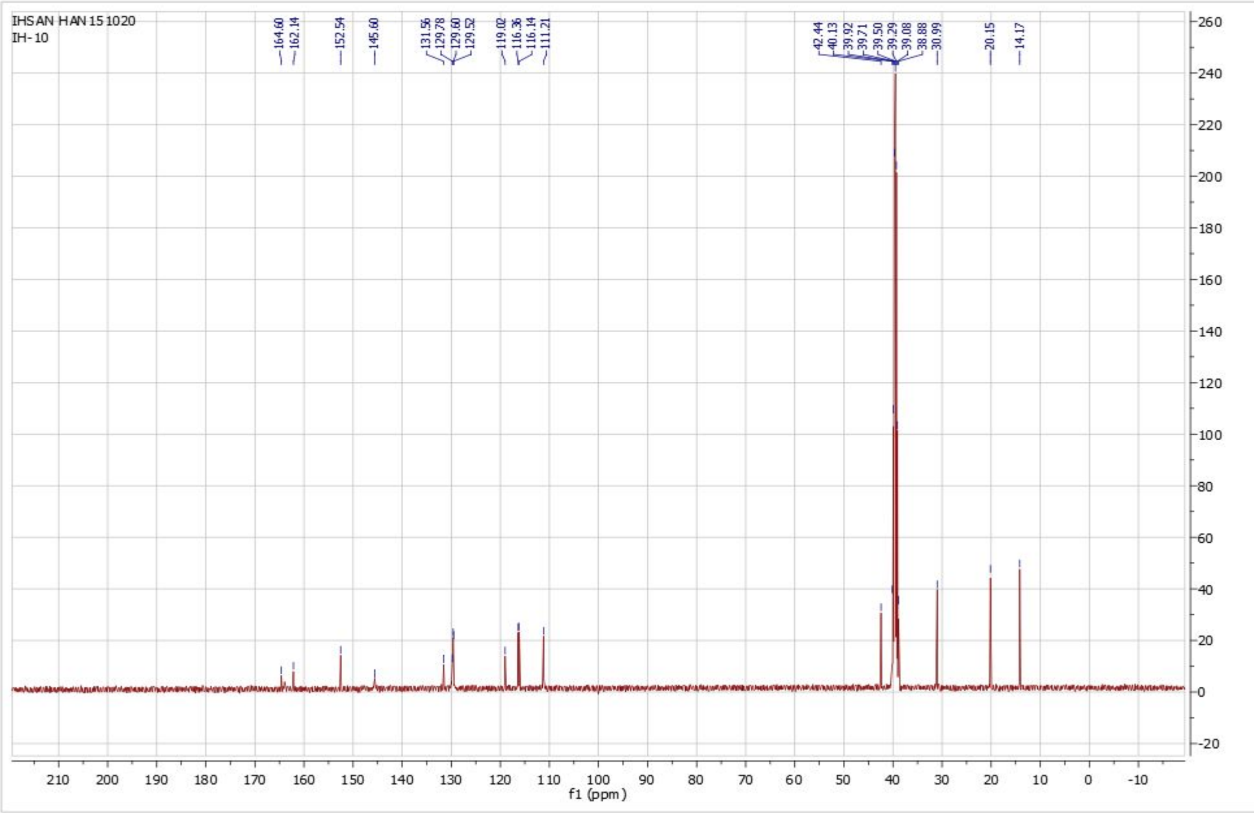

Figure S39. ^{13}C-NMR Spectra of compound **2j**

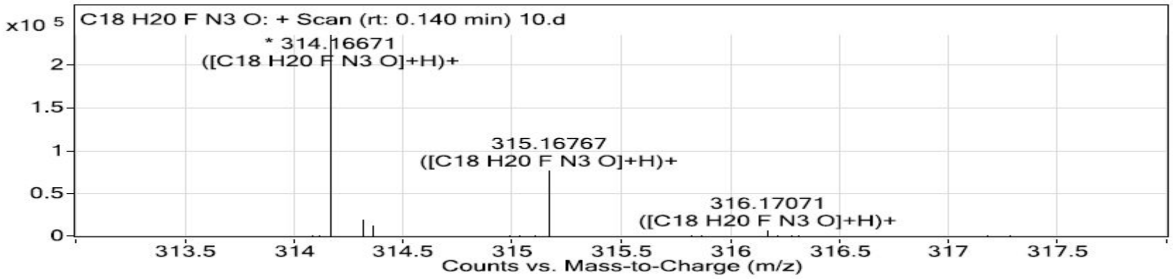

Figure S40. HR-Mass spectra of compound **2j**

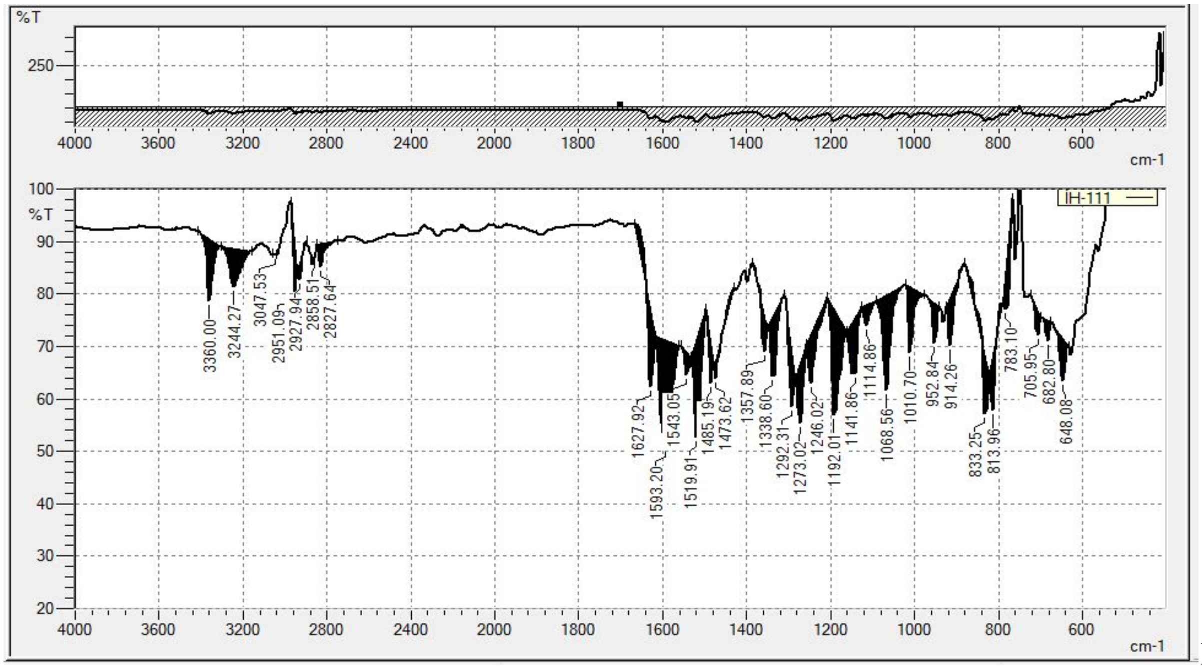
FT-

Figure S41. FT-IR spectrum of compound **2k**

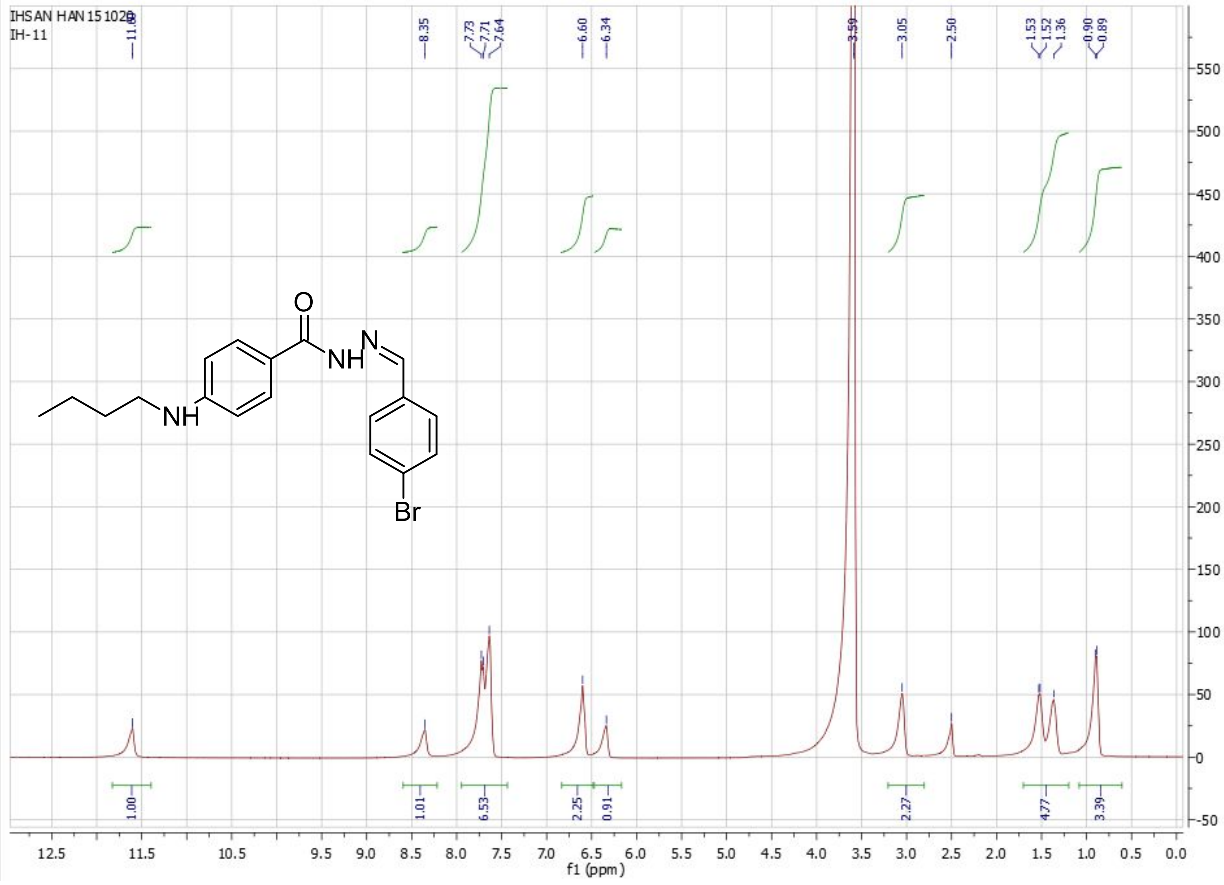

Figure S42. ^{1}H-NMR Spectra of compound **2k**

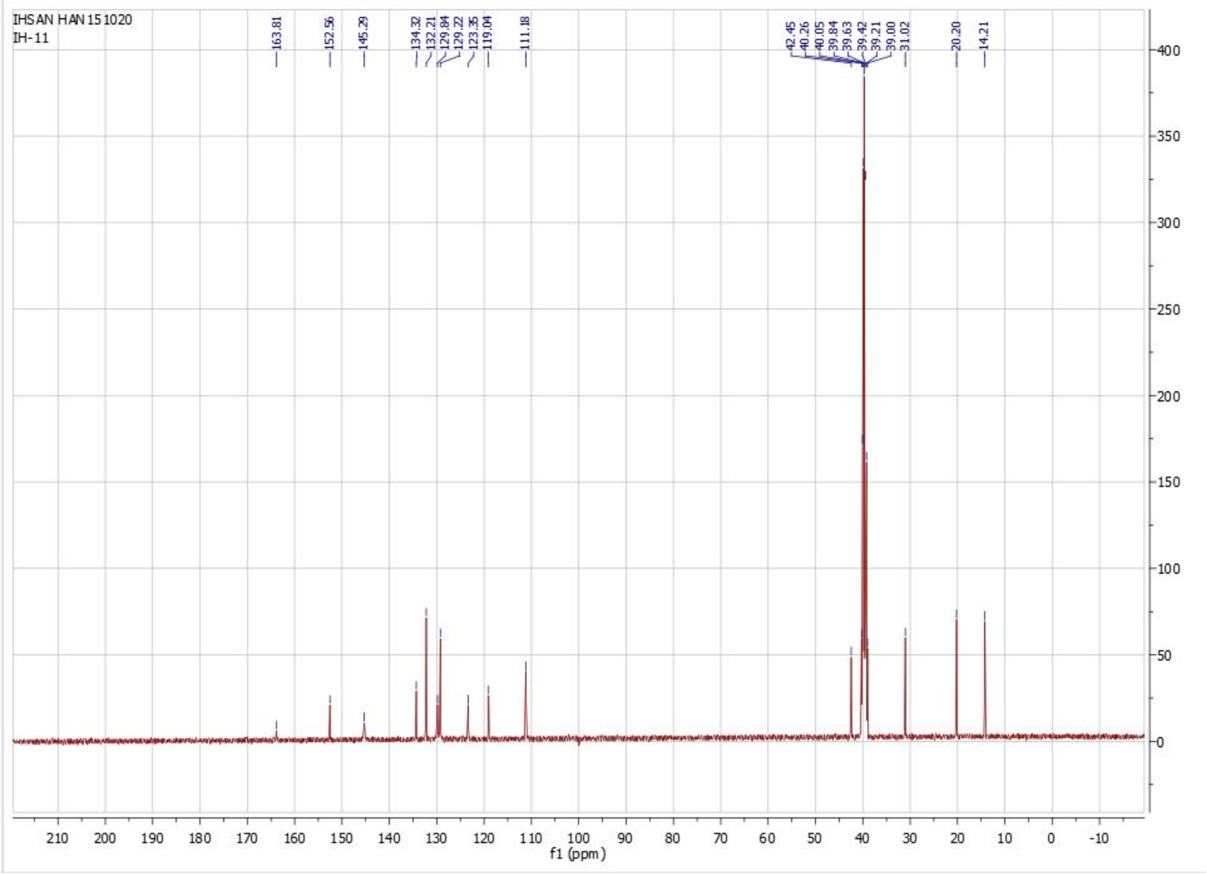

Figure S43. ^{13}C-NMR Spectra of compound **2k**

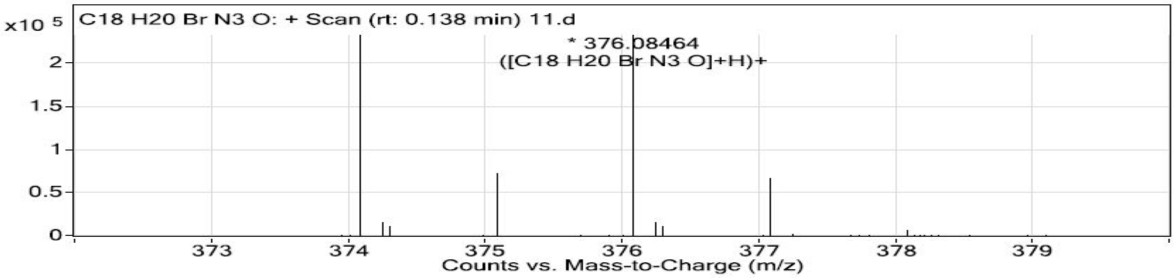

Figure S44. HR-Mass spectra of compound **2k**

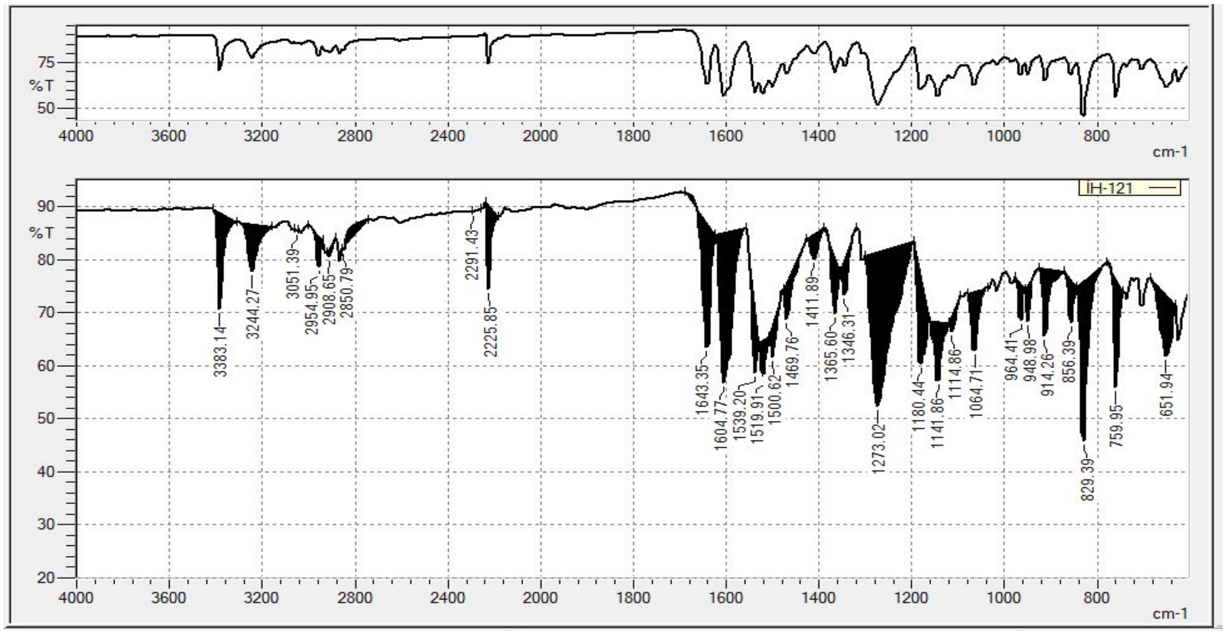

Figure S45. FT-IR spectrum of compound **2l**

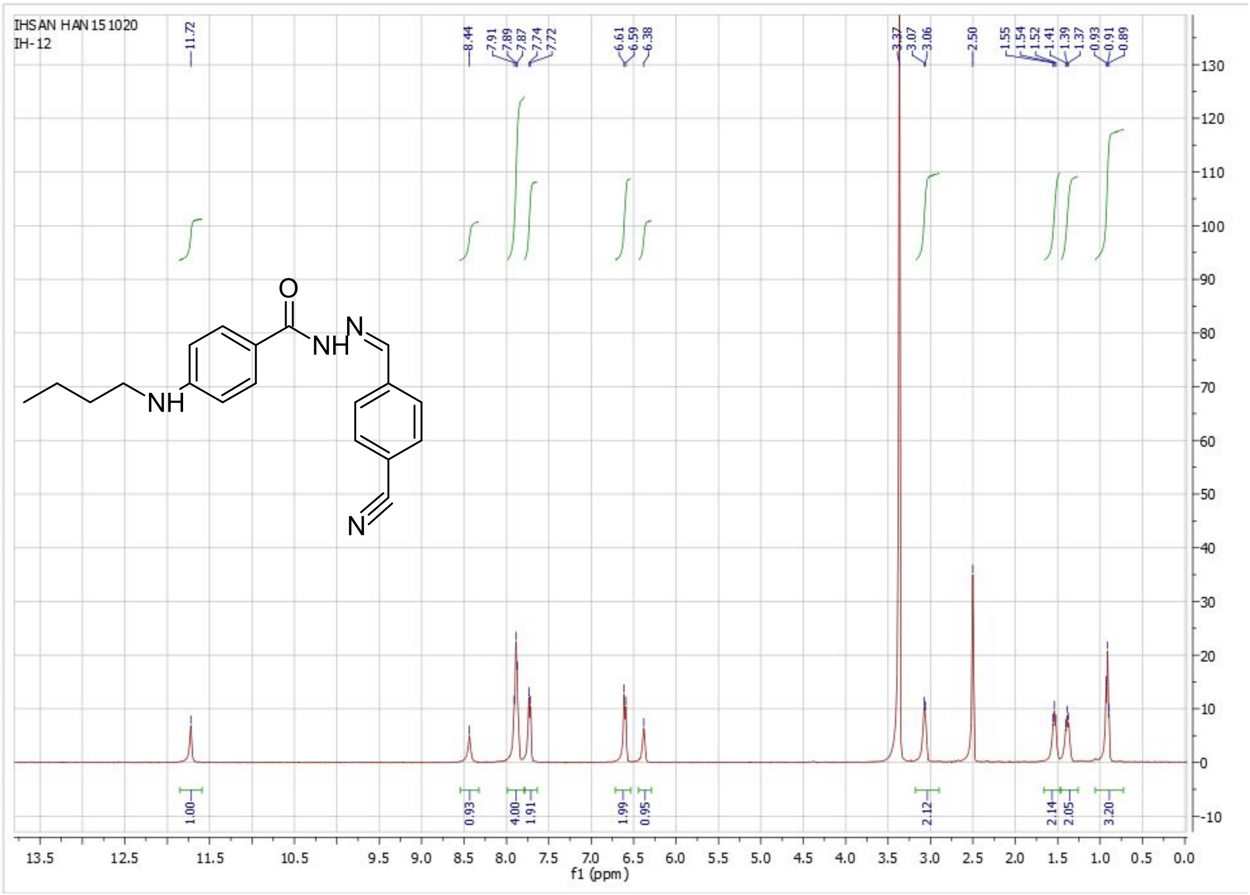

Figure S46. ^{1}H-NMR Spectra of compound **2l**

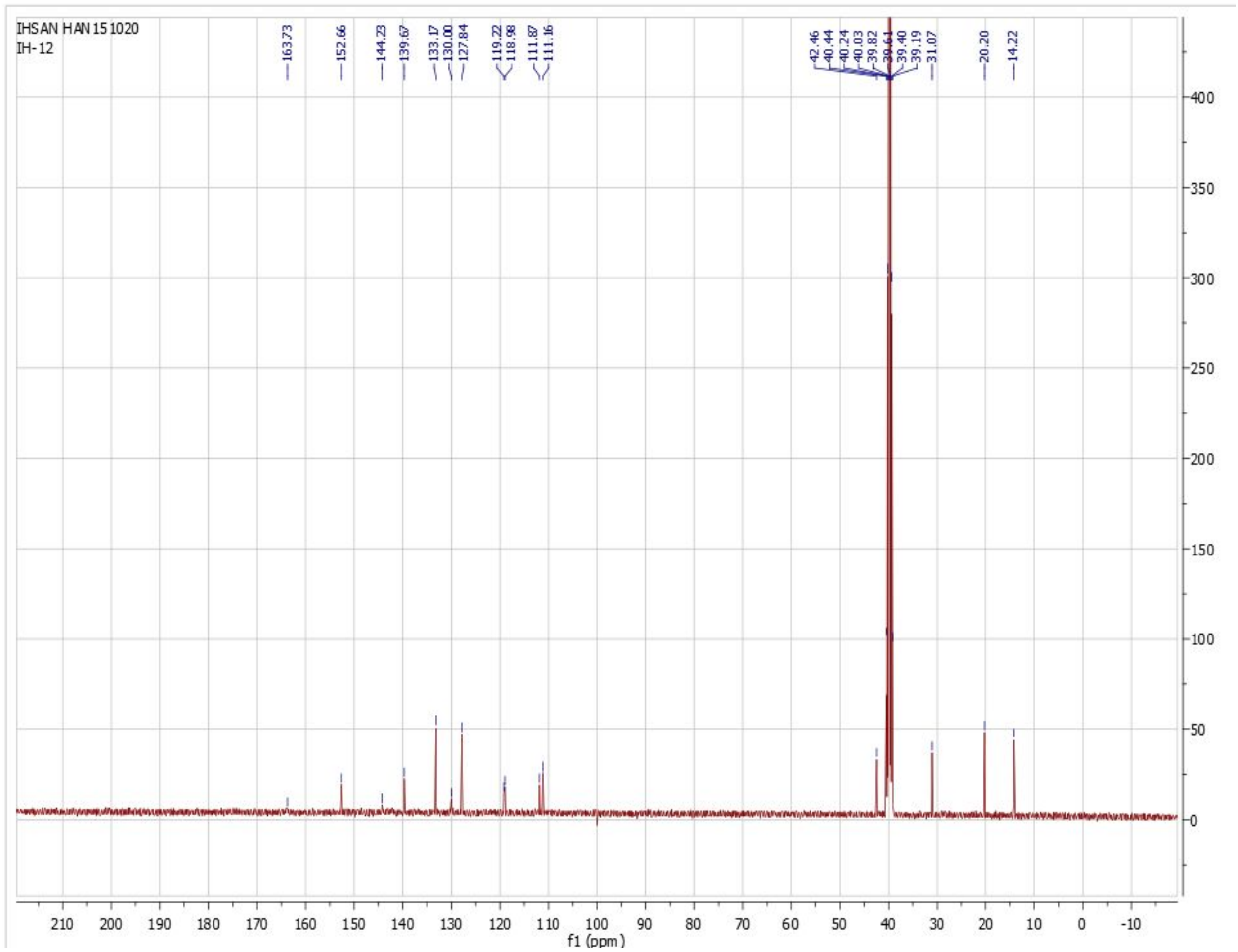

Figure S47. ^{13}C-NMR Spectra of compound **2l**

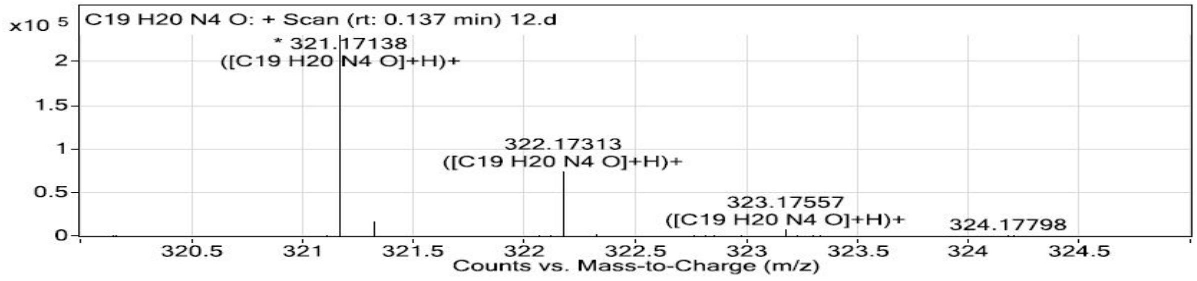

Figure S48. HR-Mass spectra of compound **2l**

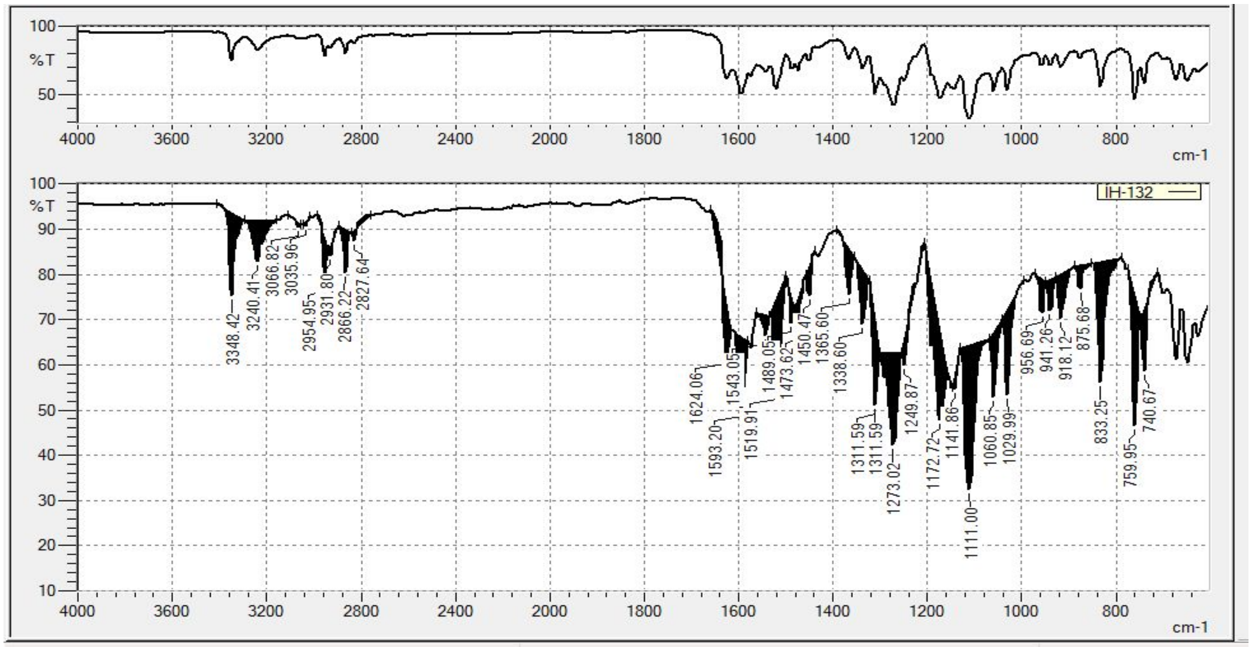

Figure S49. FT-IR spectrum of compound **2m**

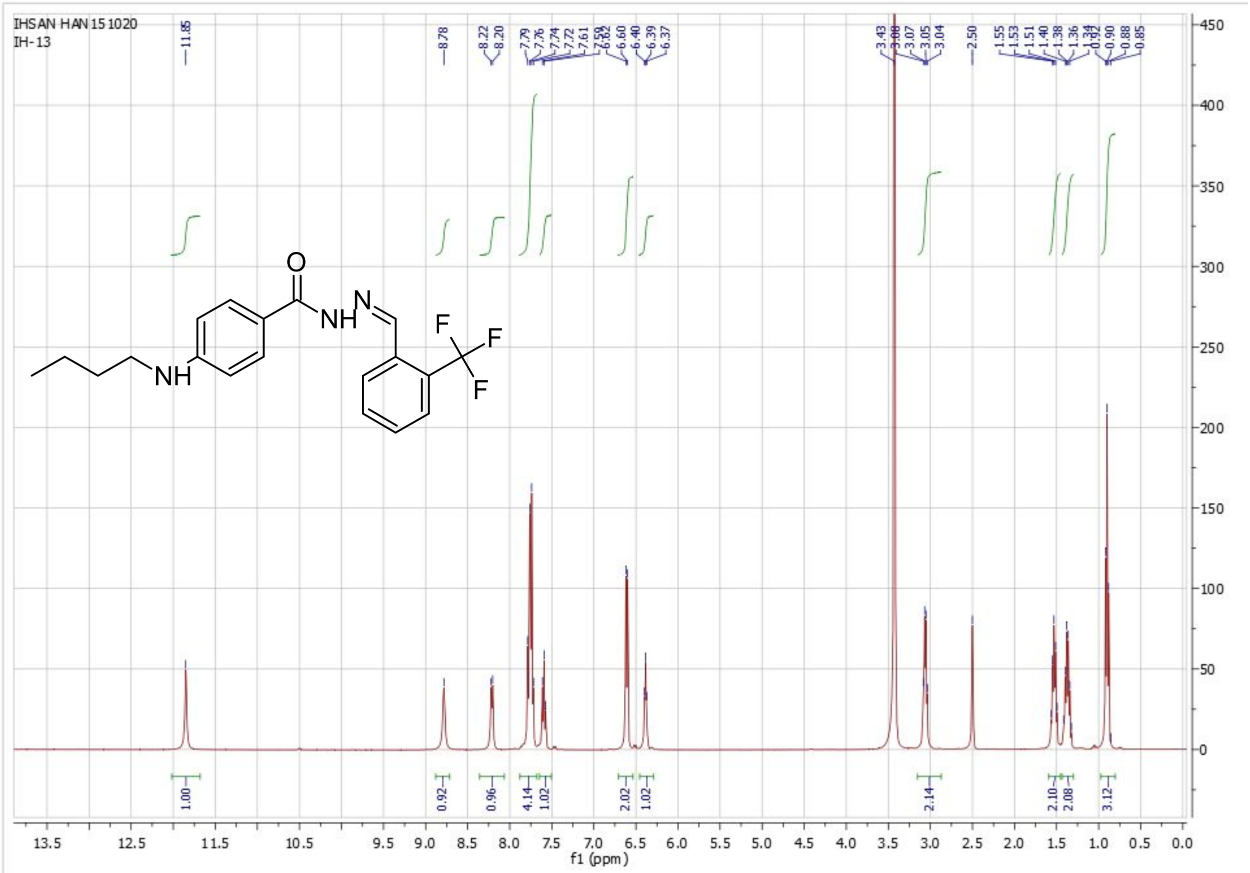

Figure S50. ^{1}H-NMR Spectra of compound **2m**

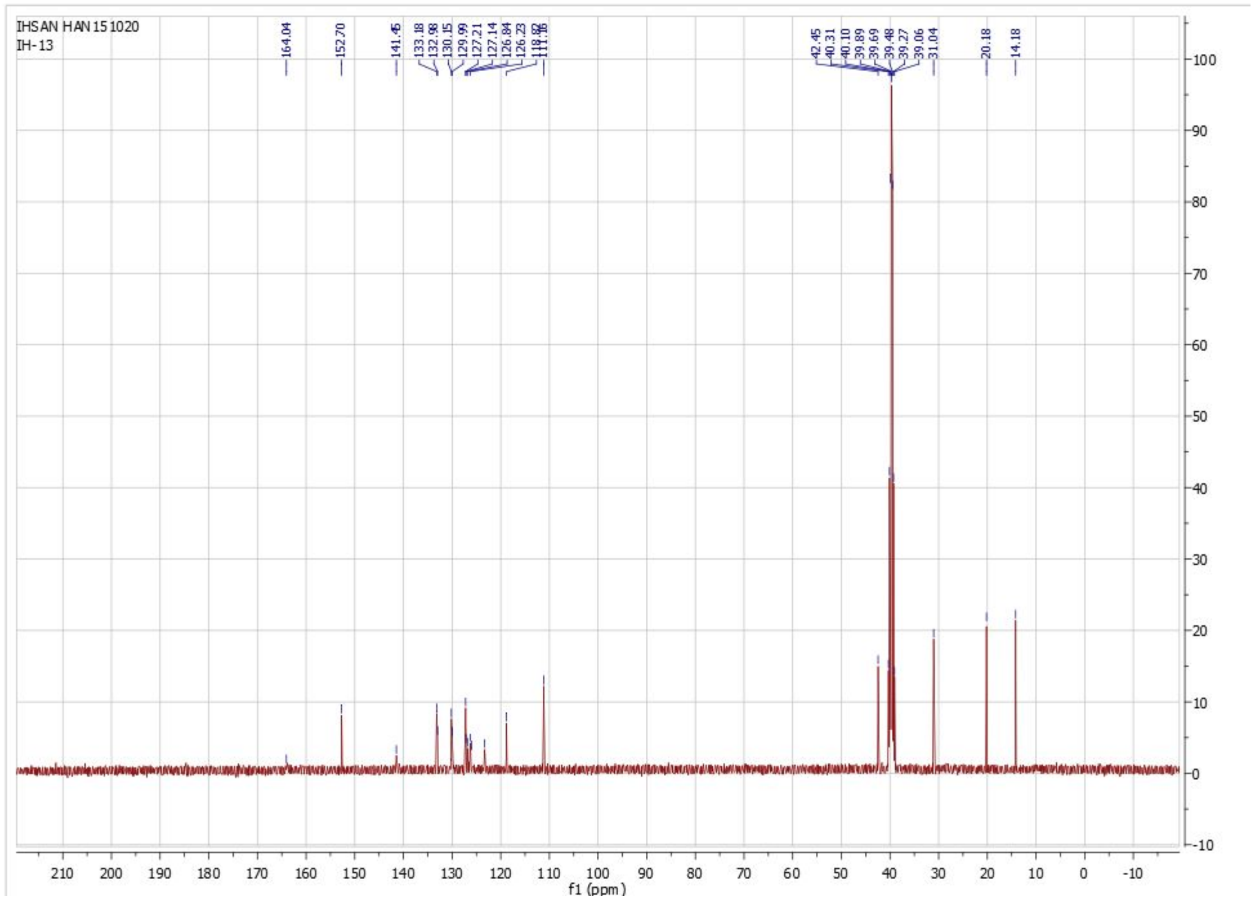

Figure S51. ^{13}C-NMR Spectra of compound **2m**

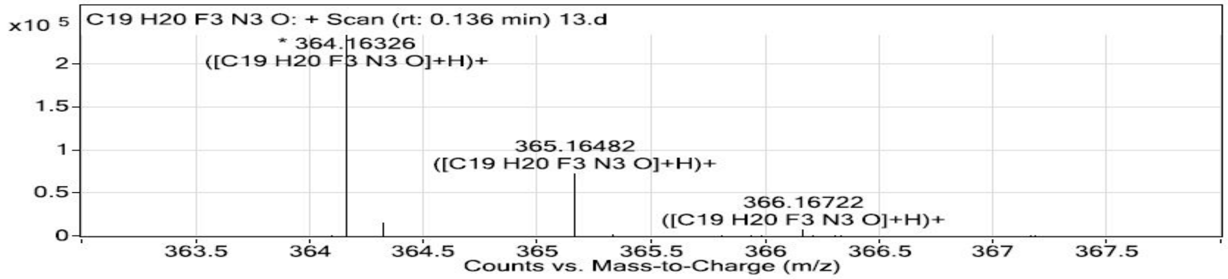

Figure S52. HR-Mass spectra of compound **2m**

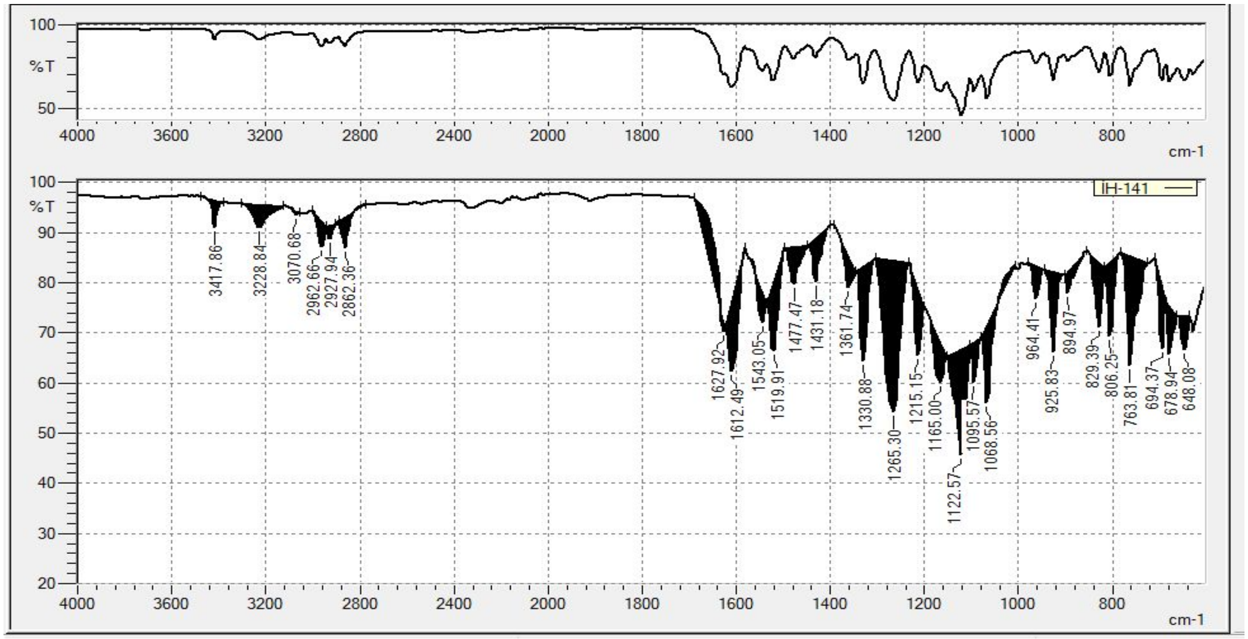

Figure S53. FT-IR spectrum of compound **2n**

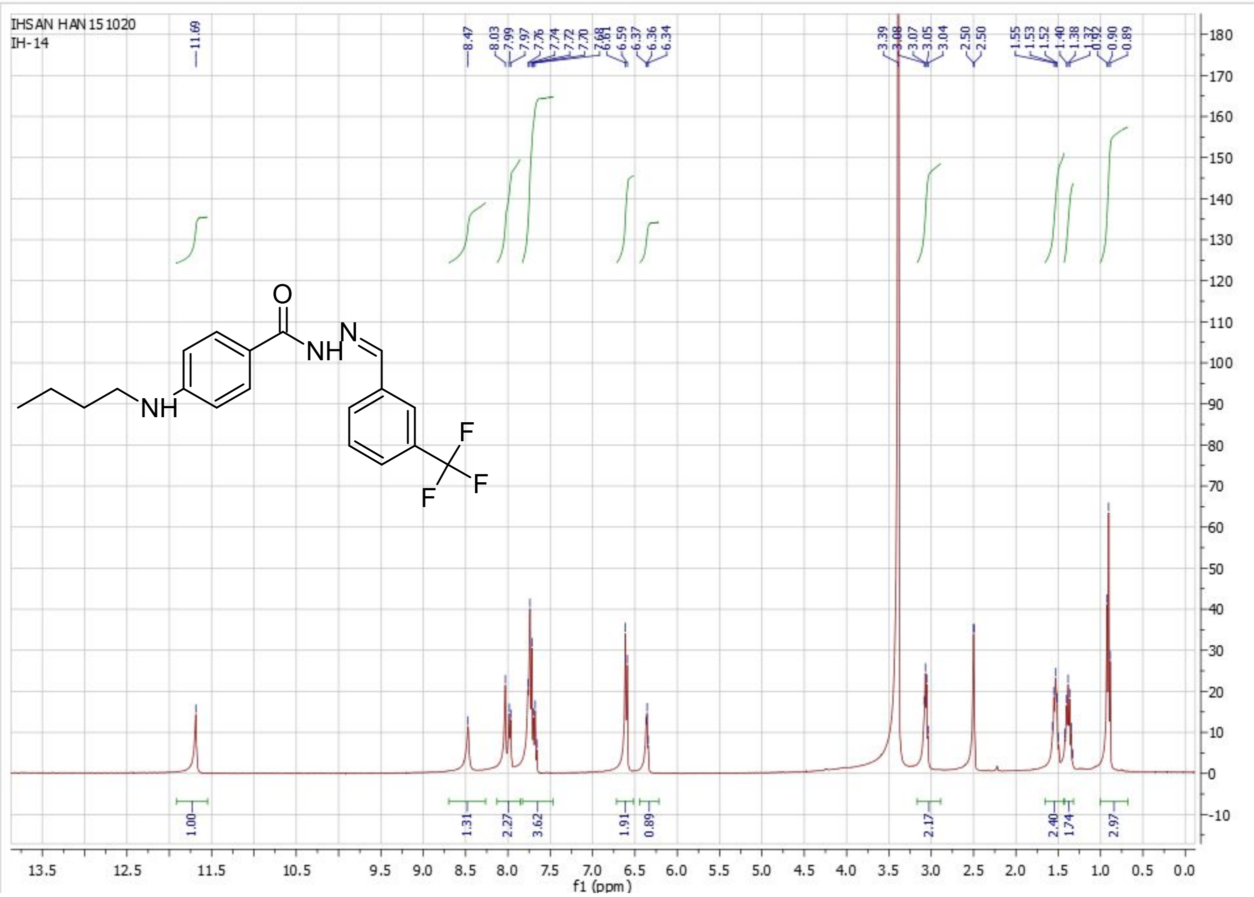

Figure S54. ^{1}H-NMR Spectra of compound **2n**

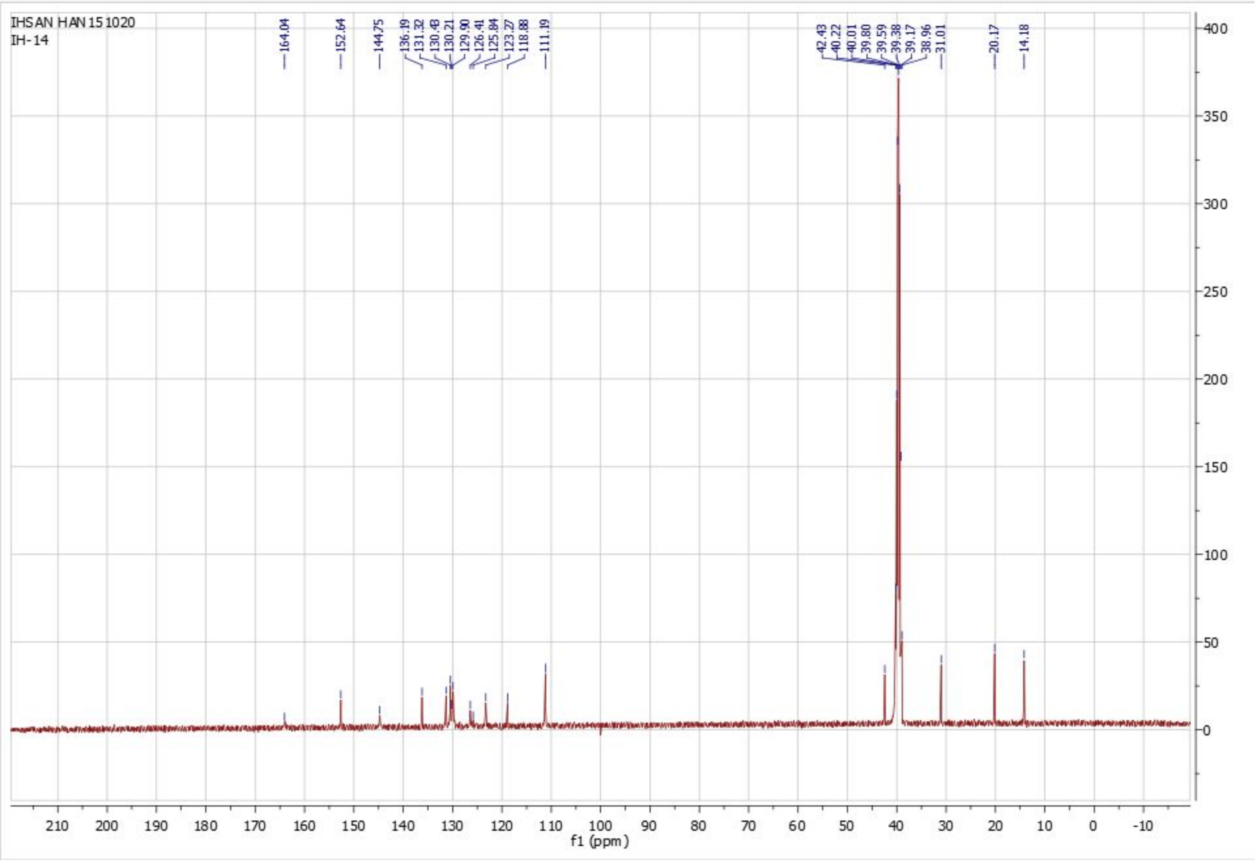

Figure S55. ^{13}C-NMR Spectra of compound **2n**

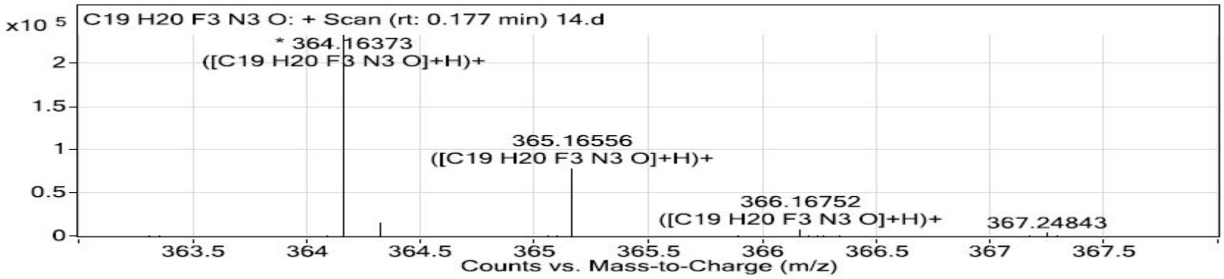

Figure S56. HR-Mass spectra of compound **2n**

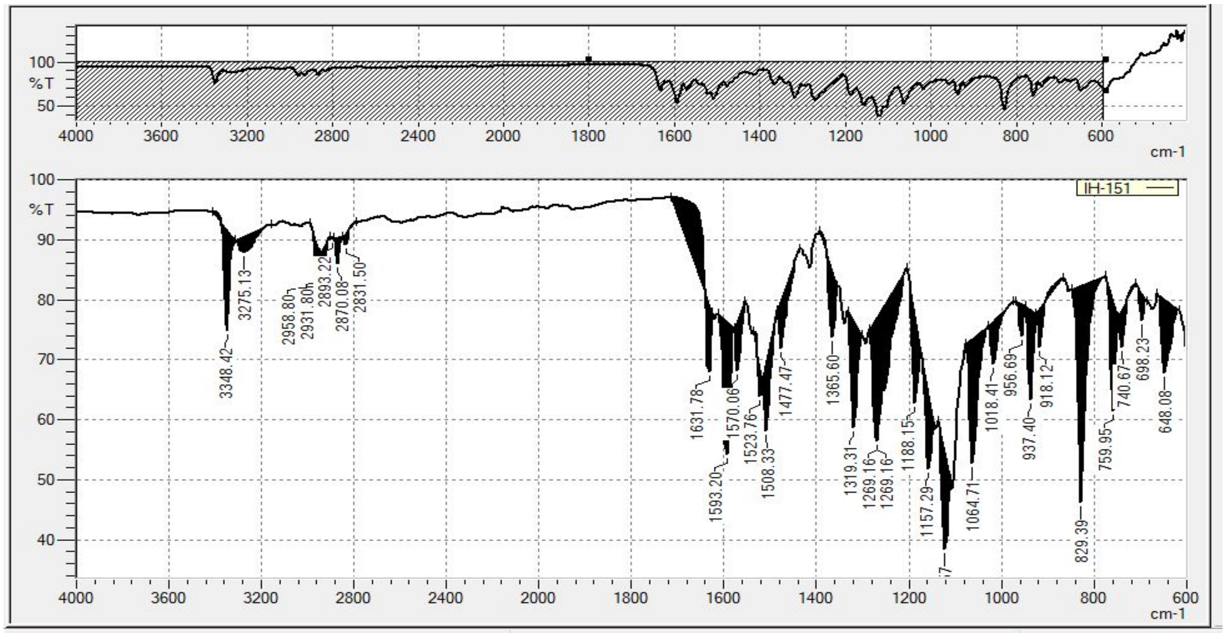

Figure S57. FT-IR spectrum of compound **2o**

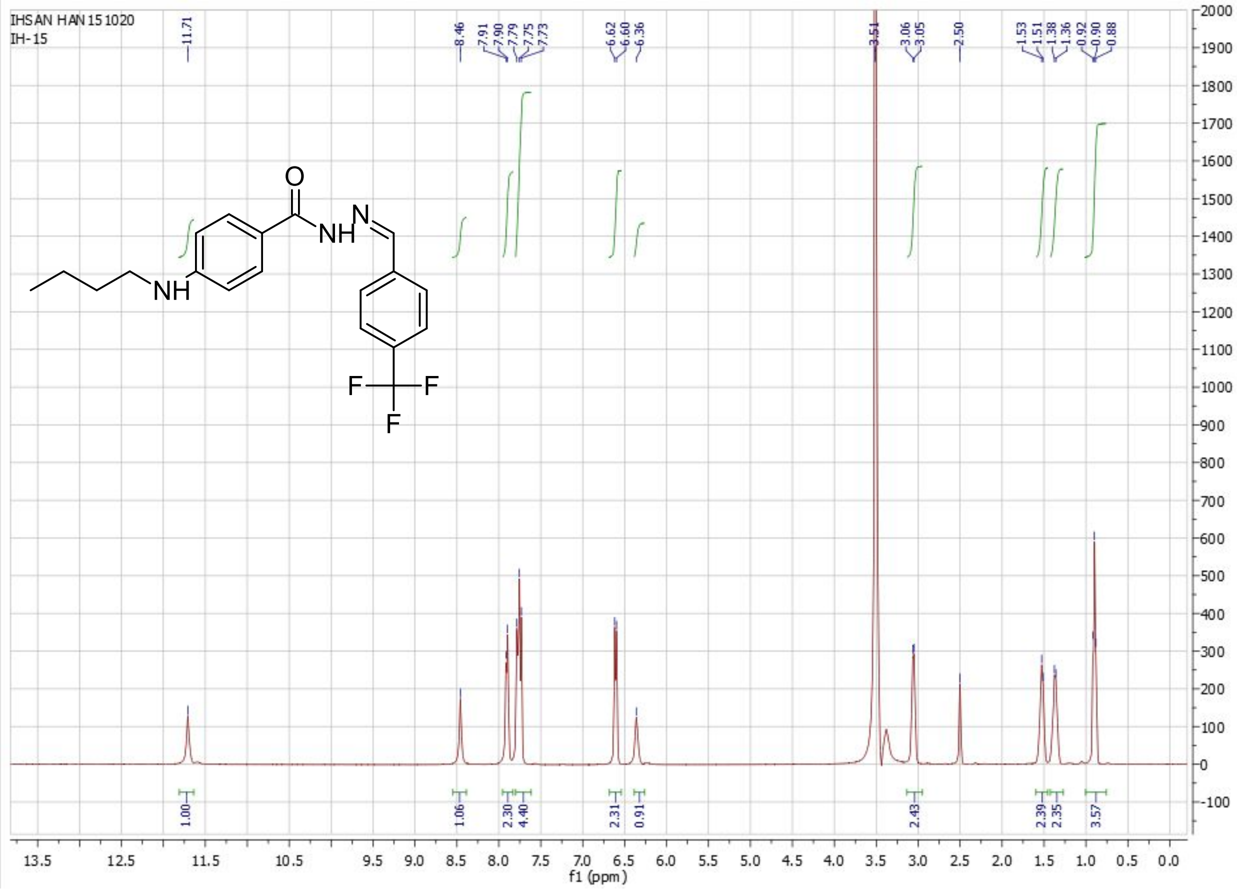

Figure S58. ^{1}H-NMR Spectra of compound **2o**

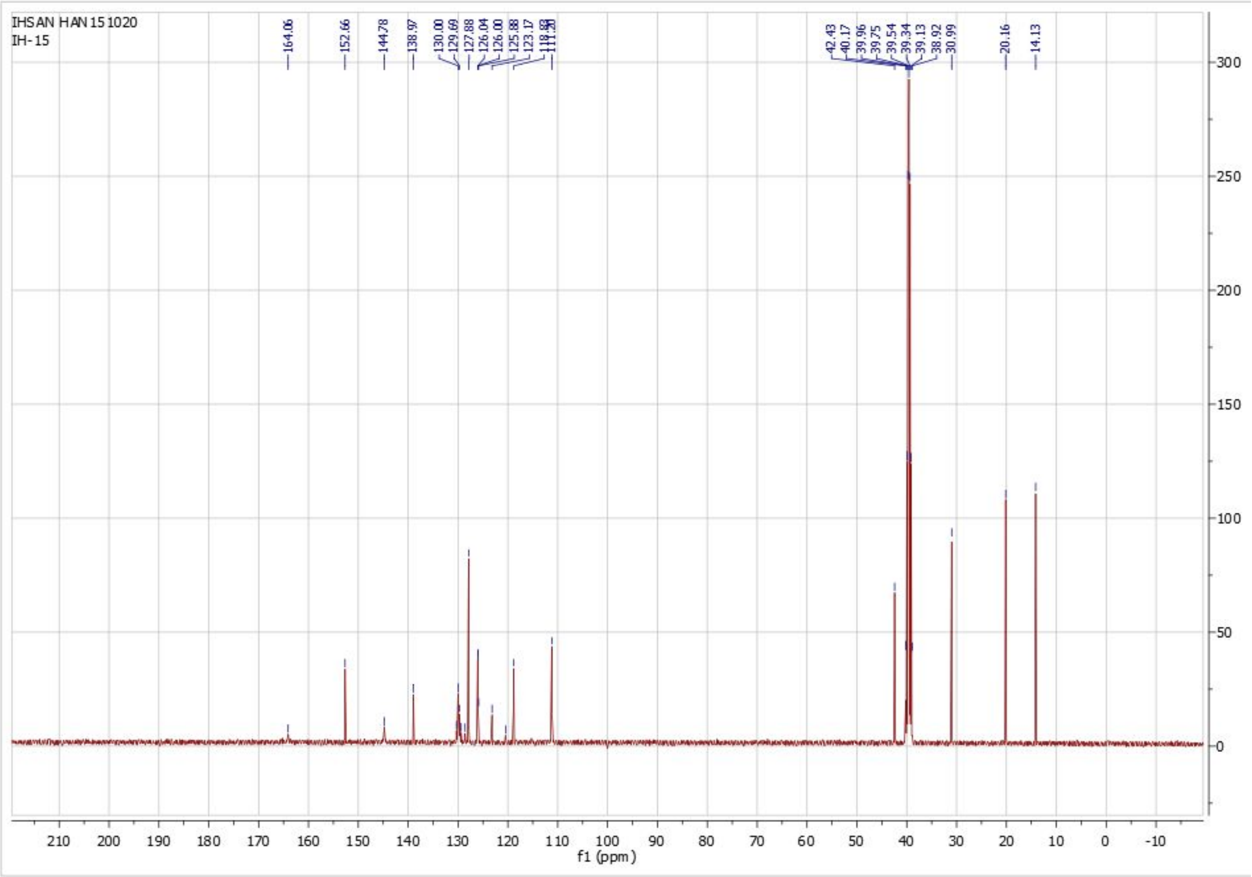

Figure S59. ^{13}C-NMR Spectra of compound **2o**

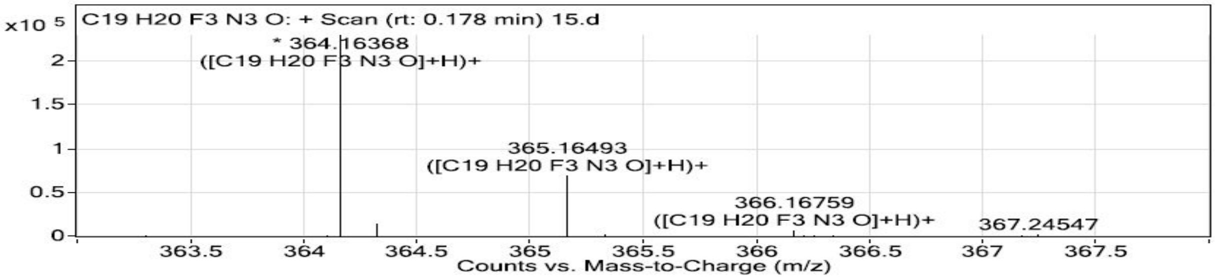

Figure S60. HR-Mass spectra of compound **2o**

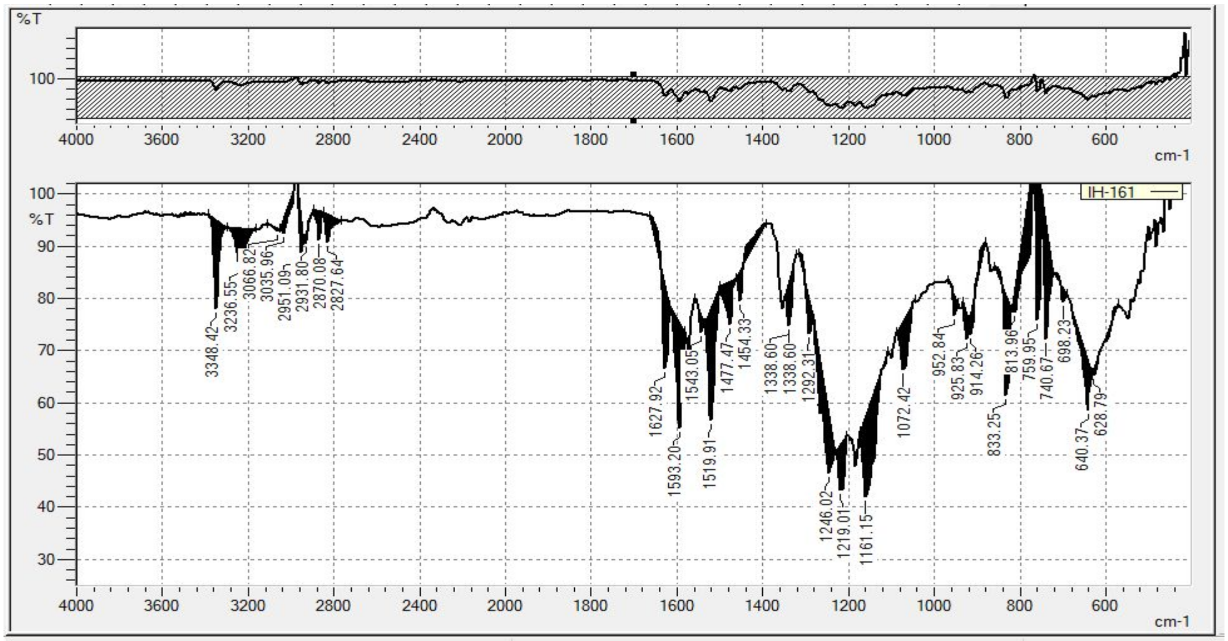

Figure S61. FT-IR spectrum of compound **2p**

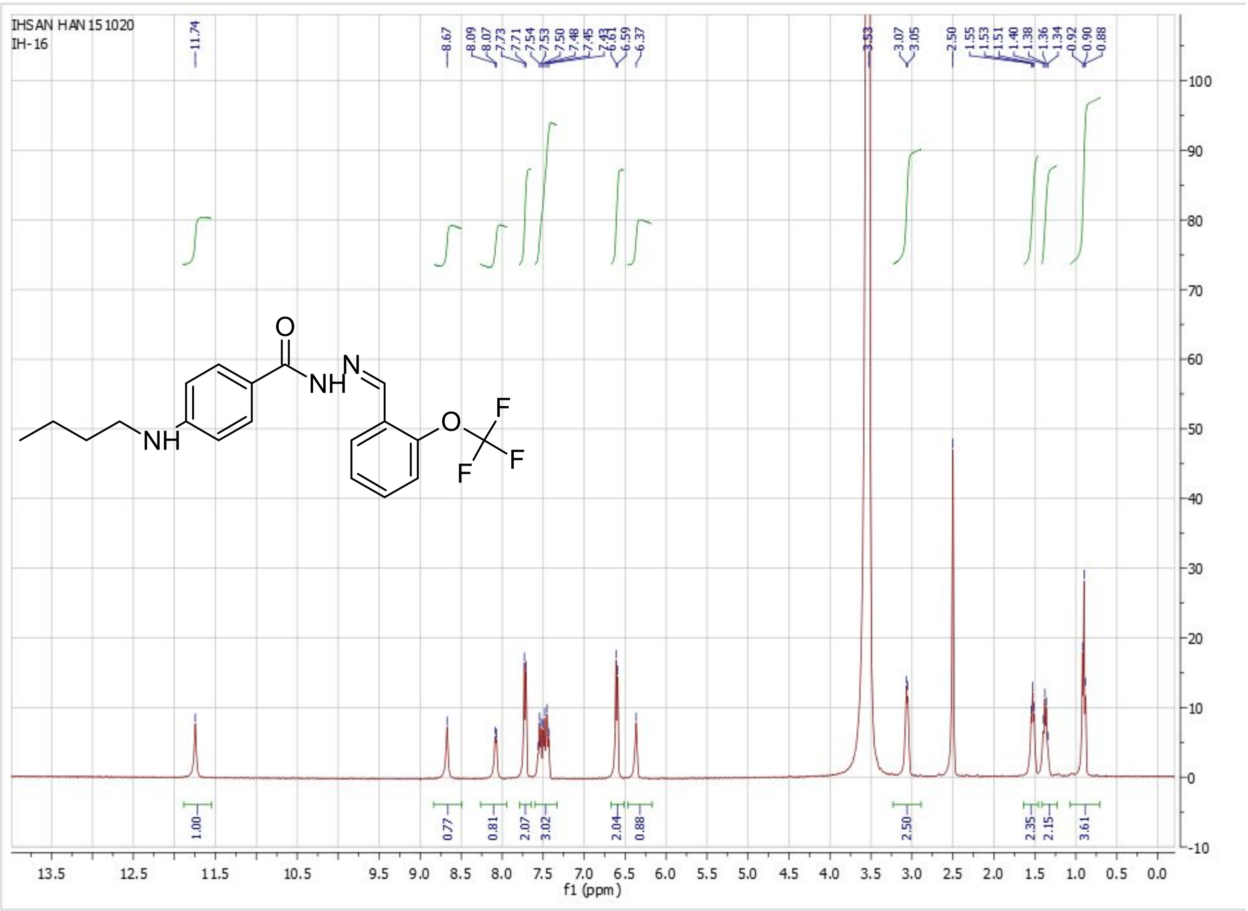

Figure S62. ^{1}H-NMR Spectra of compound **2p**

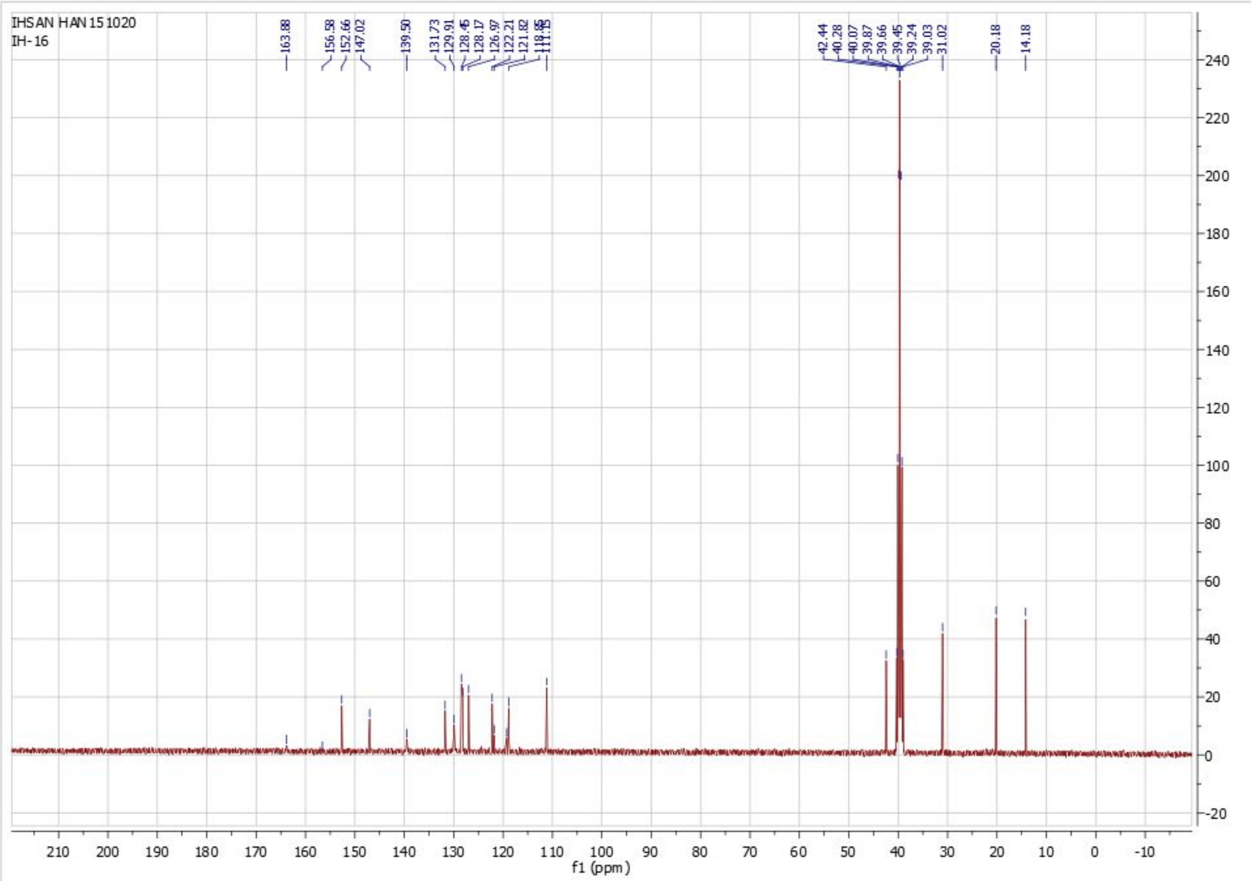

Figure S63. ^{13}C-NMR Spectra of compound **2p**

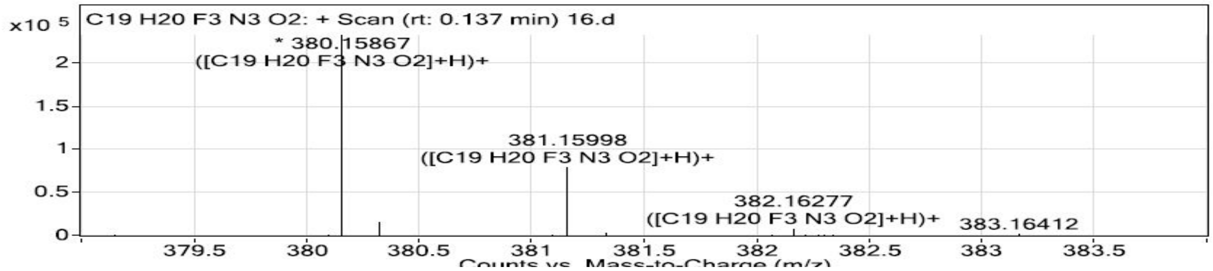

Figure S64. HR-Mass spectra of compound **2p**

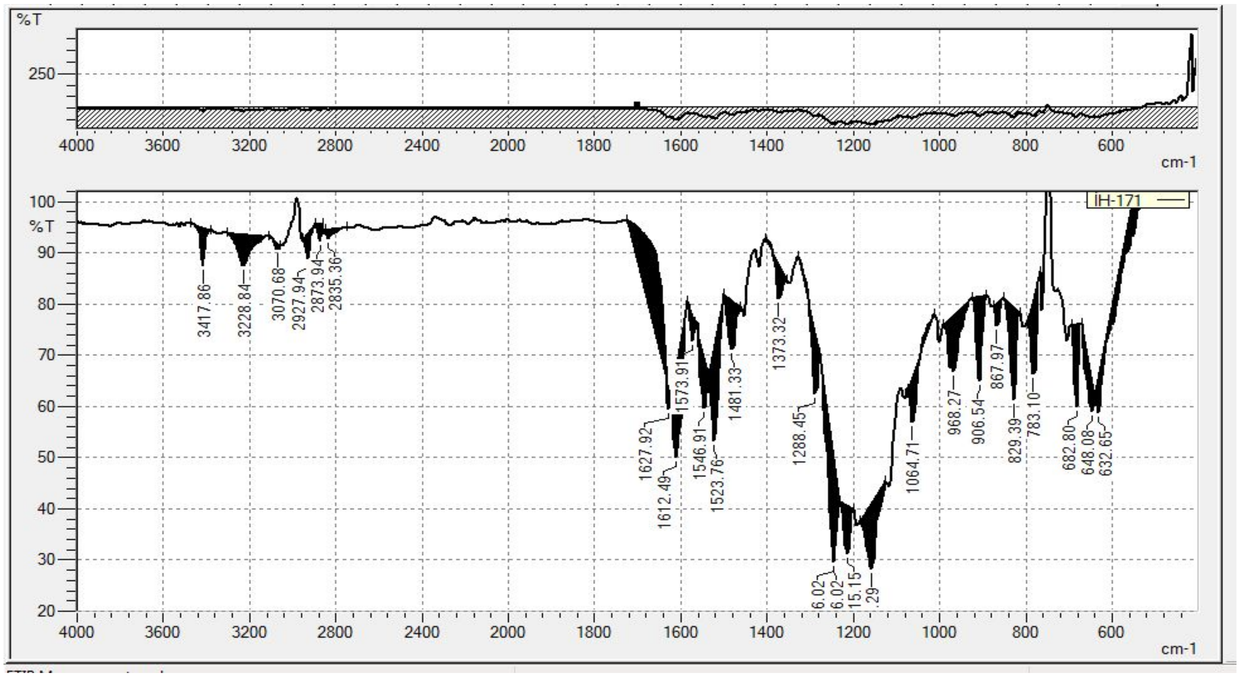

Figure S65. FT-IR spectrum of compound **2q**

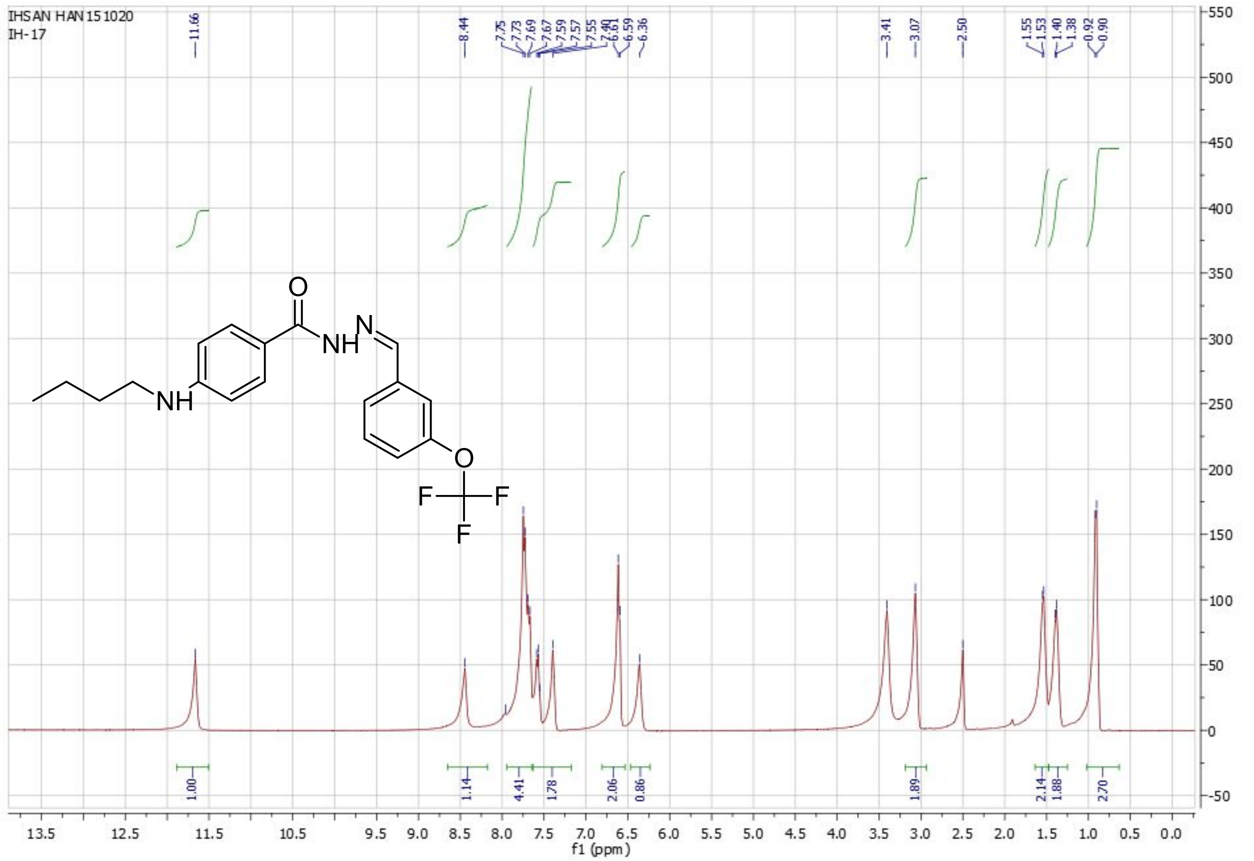

Figure S66. ^{1}H-NMR Spectra of compound **2q**

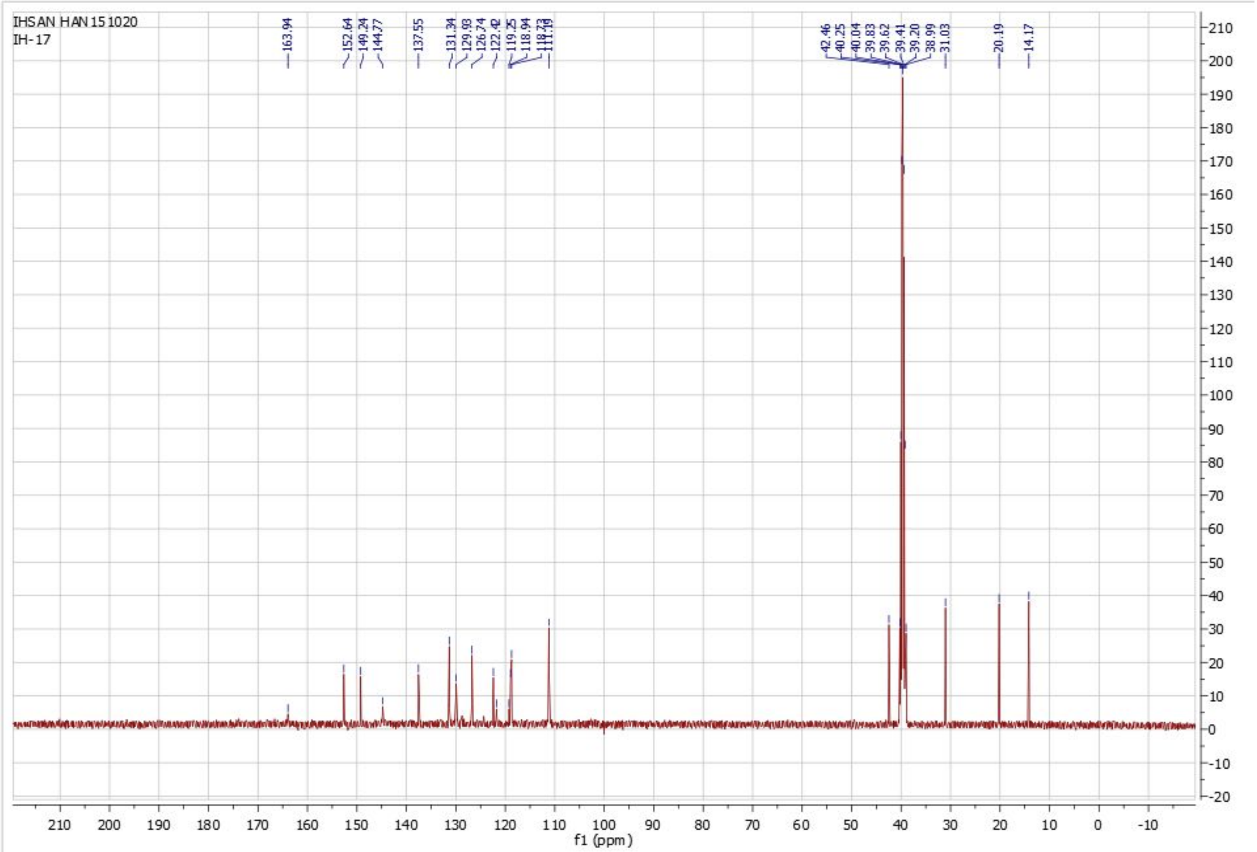

Figure S67. ^{13}C-NMR Spectra of compound **2q**

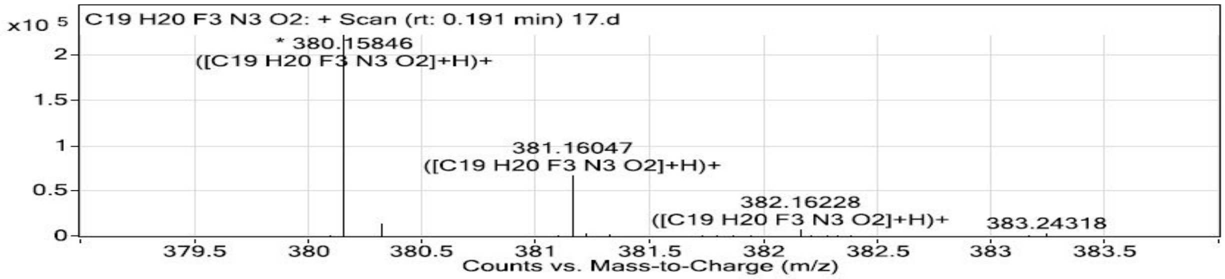

Figure S68. HR-Mass spectra of compound **2q**

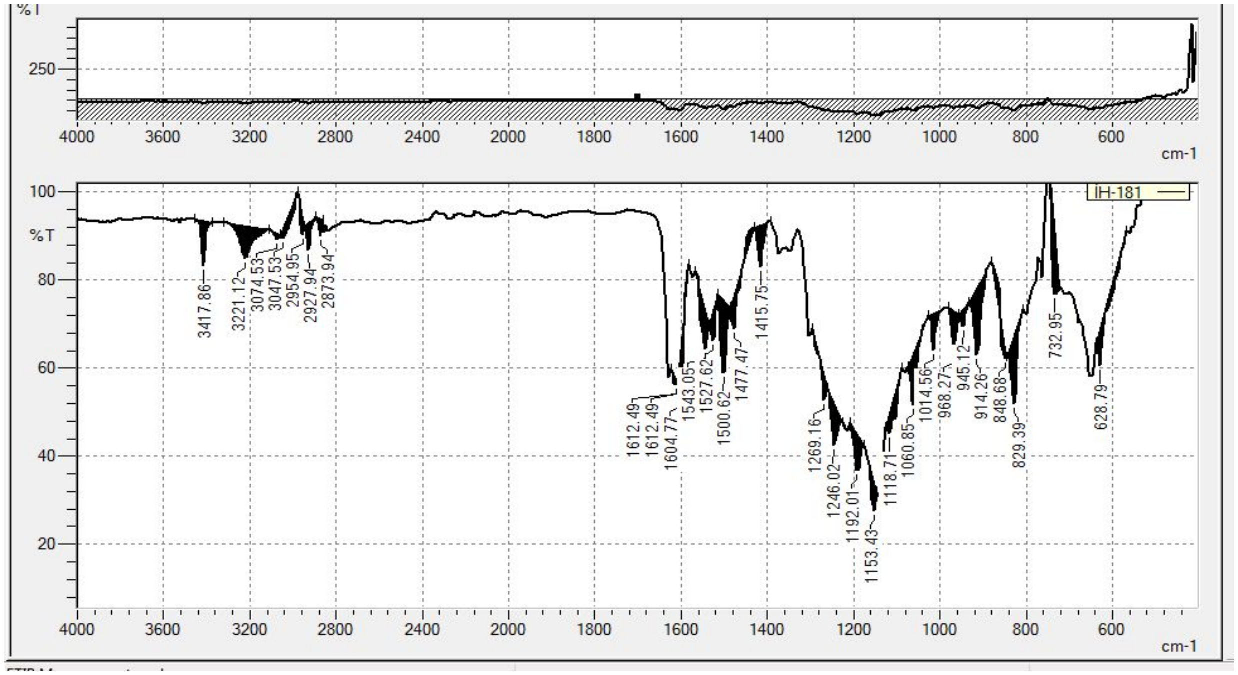

Figure S69. FT-IR spectrum of compound **2r**

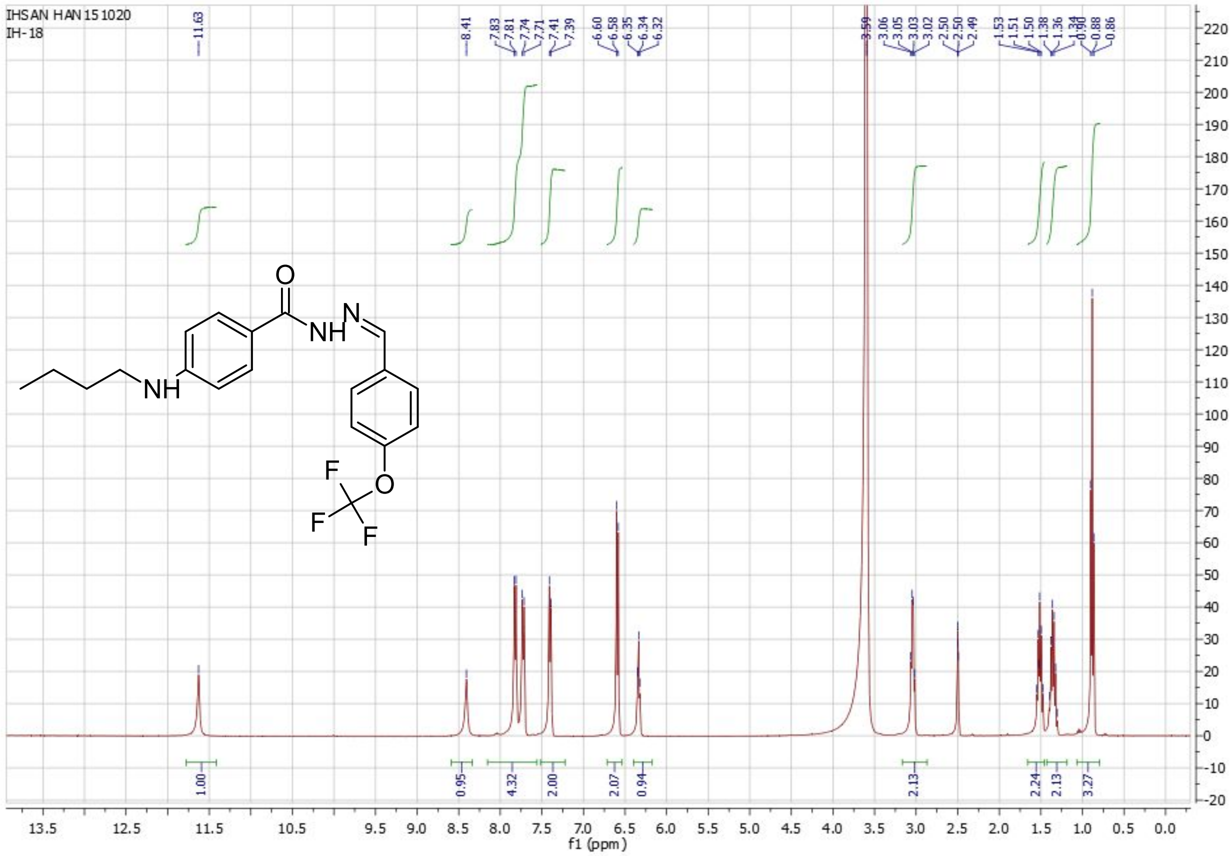

Figure S70. ^{1}H-NMR Spectra of compound **2r**

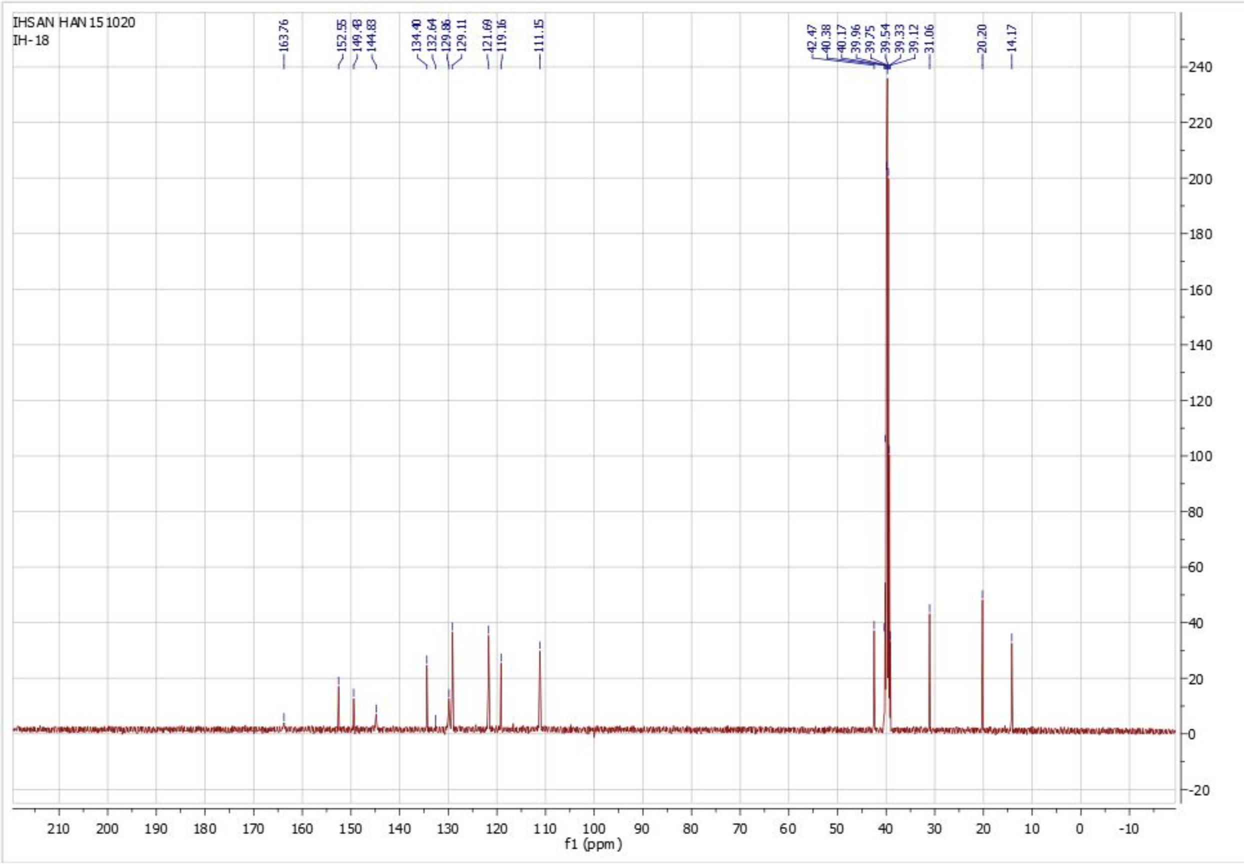

Figure S71. ^{13}C-NMR Spectra of compound **2r**

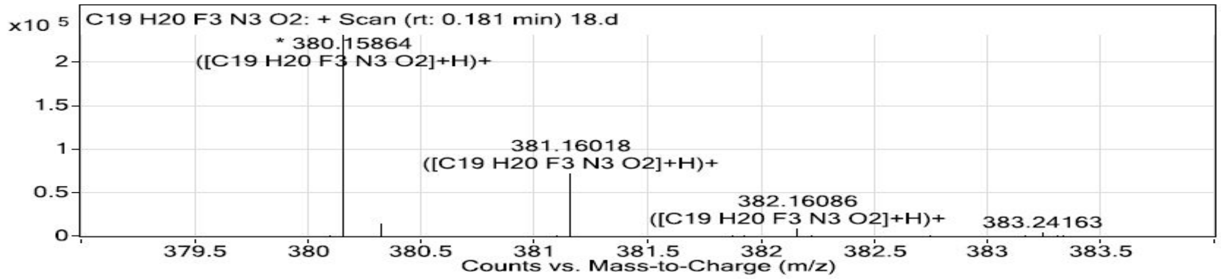

Figure S72. HR-Mass spectra of compound **2r**

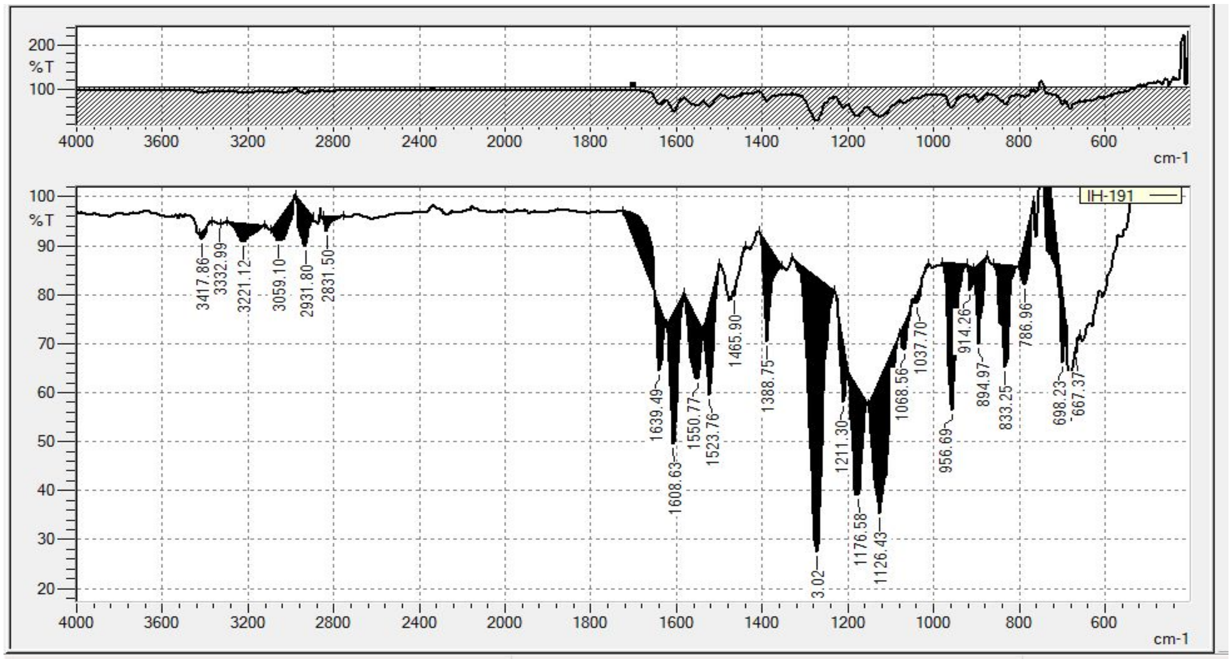

Figure S73. FT-IR spectrum of compound **2s**

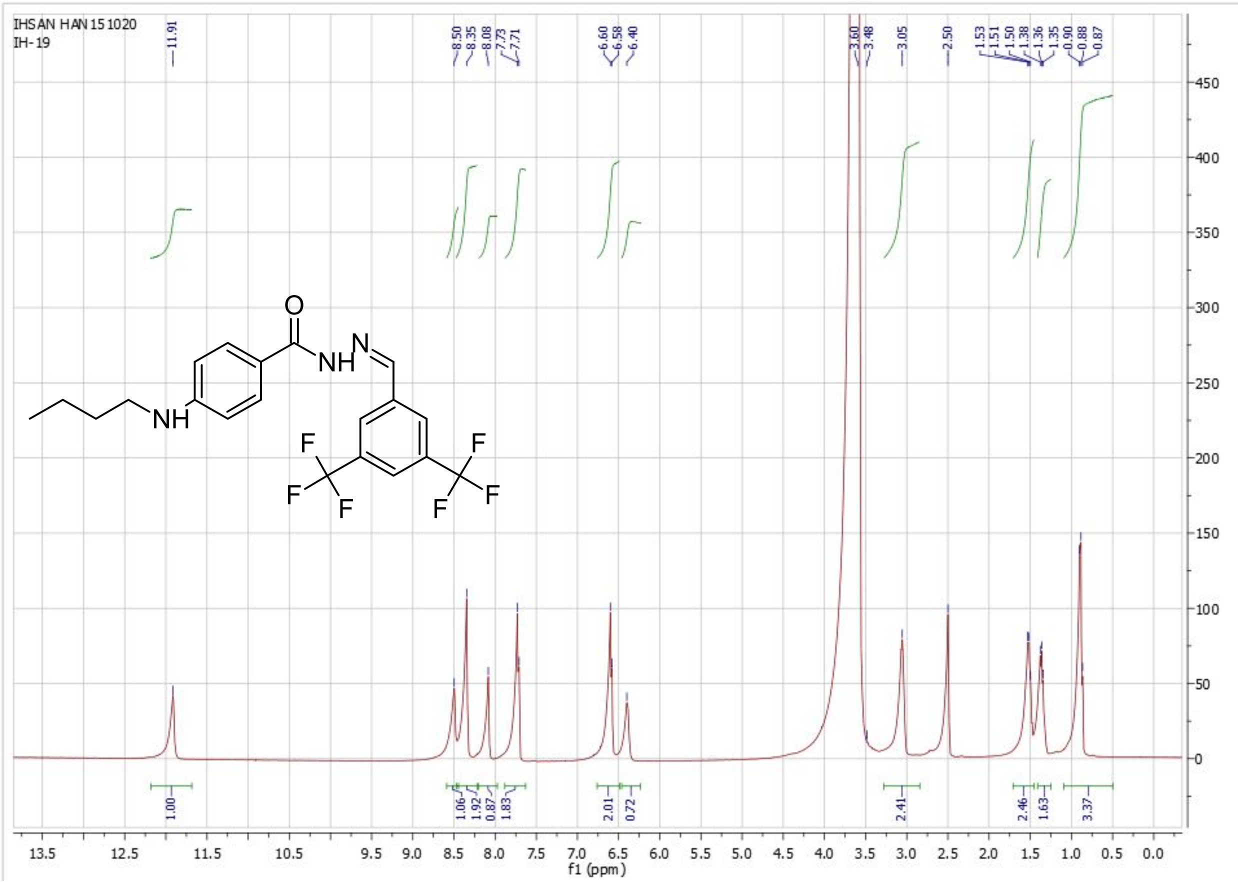

Figure S74. ^{1}H-NMR Spectra of compound **2s**

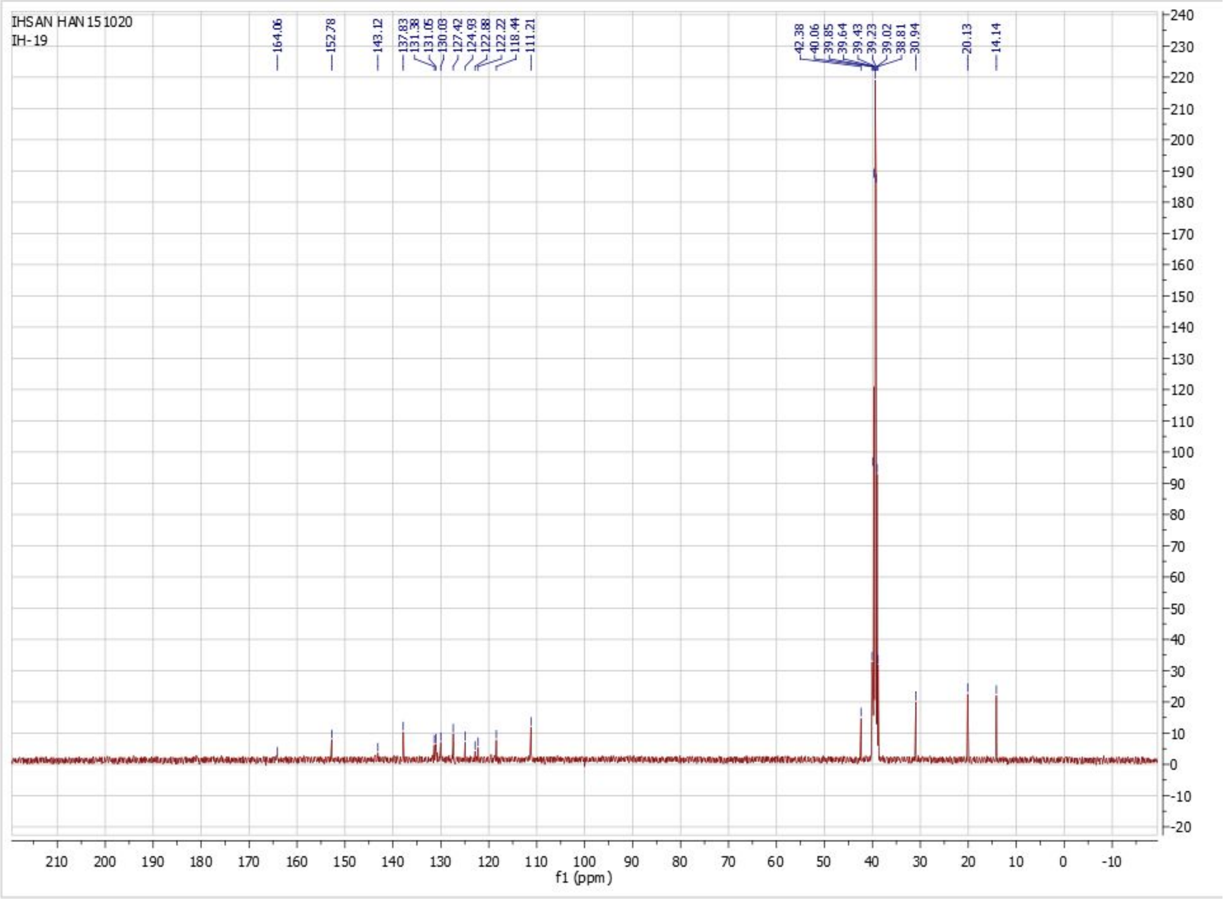

Figure S75. ^{13}C-NMR Spectra of compound **2s**

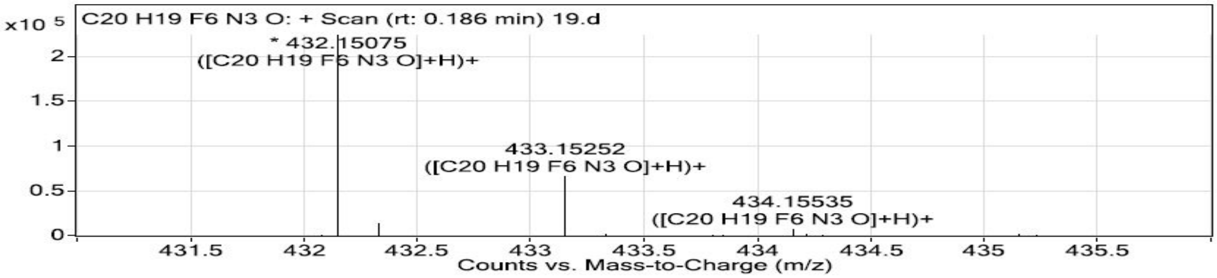

Figure S76. HR-Mass spectra of compound **2s**

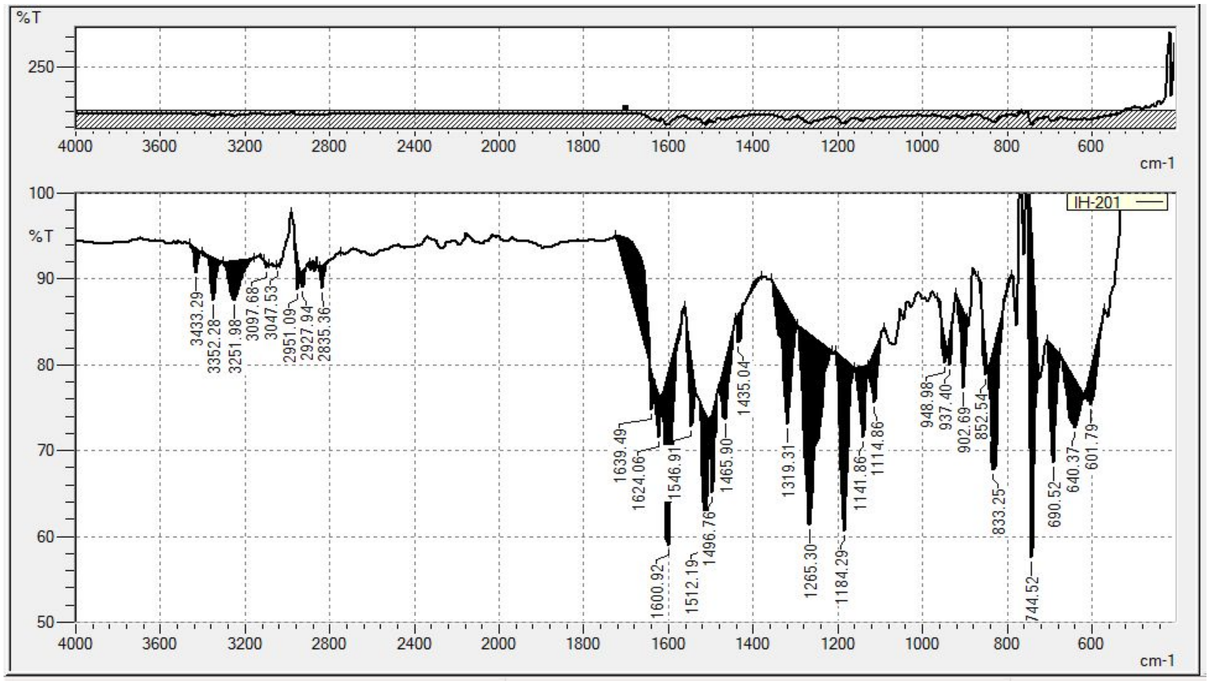

Figure S77. FT-IR spectrum of compound **2t**

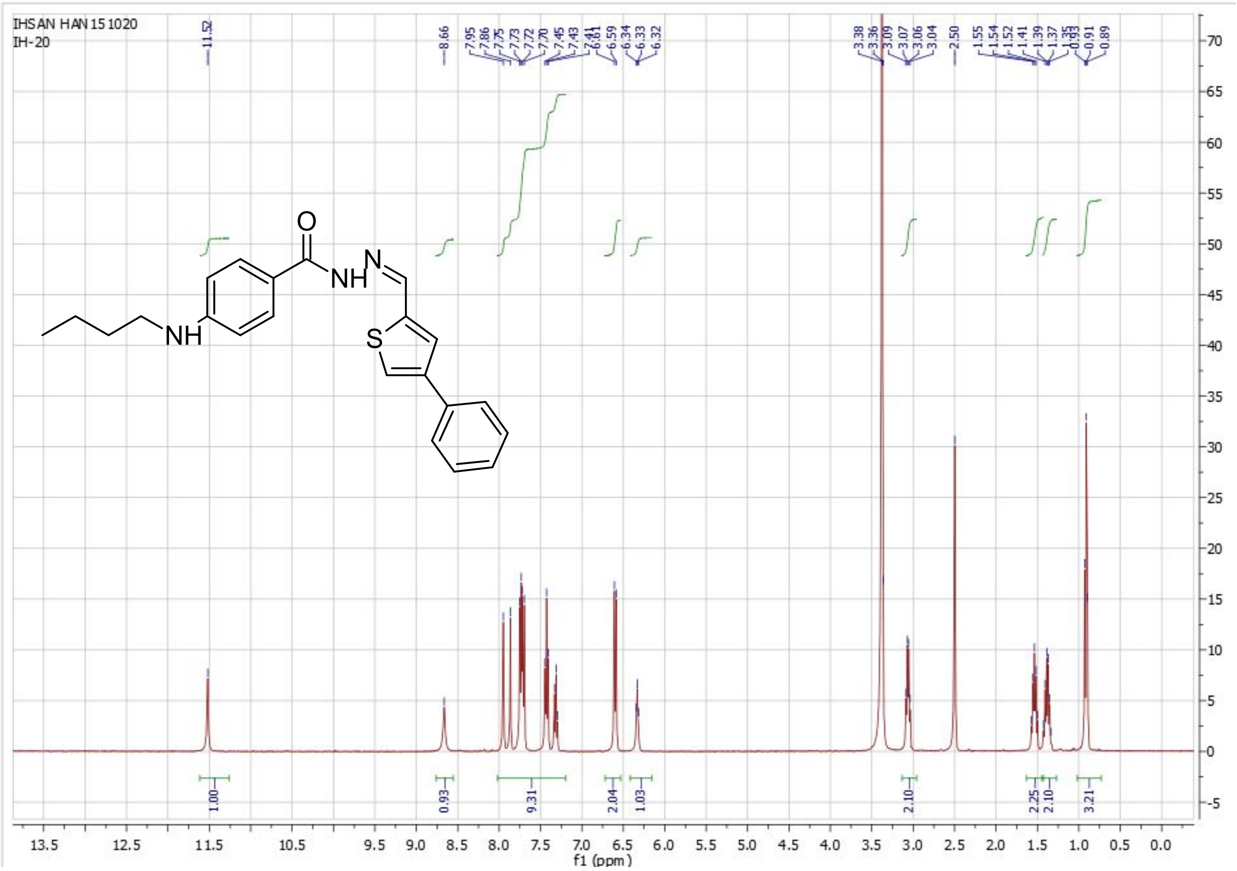

Figure S78. ^{1}H-NMR Spectra of compound **2t**

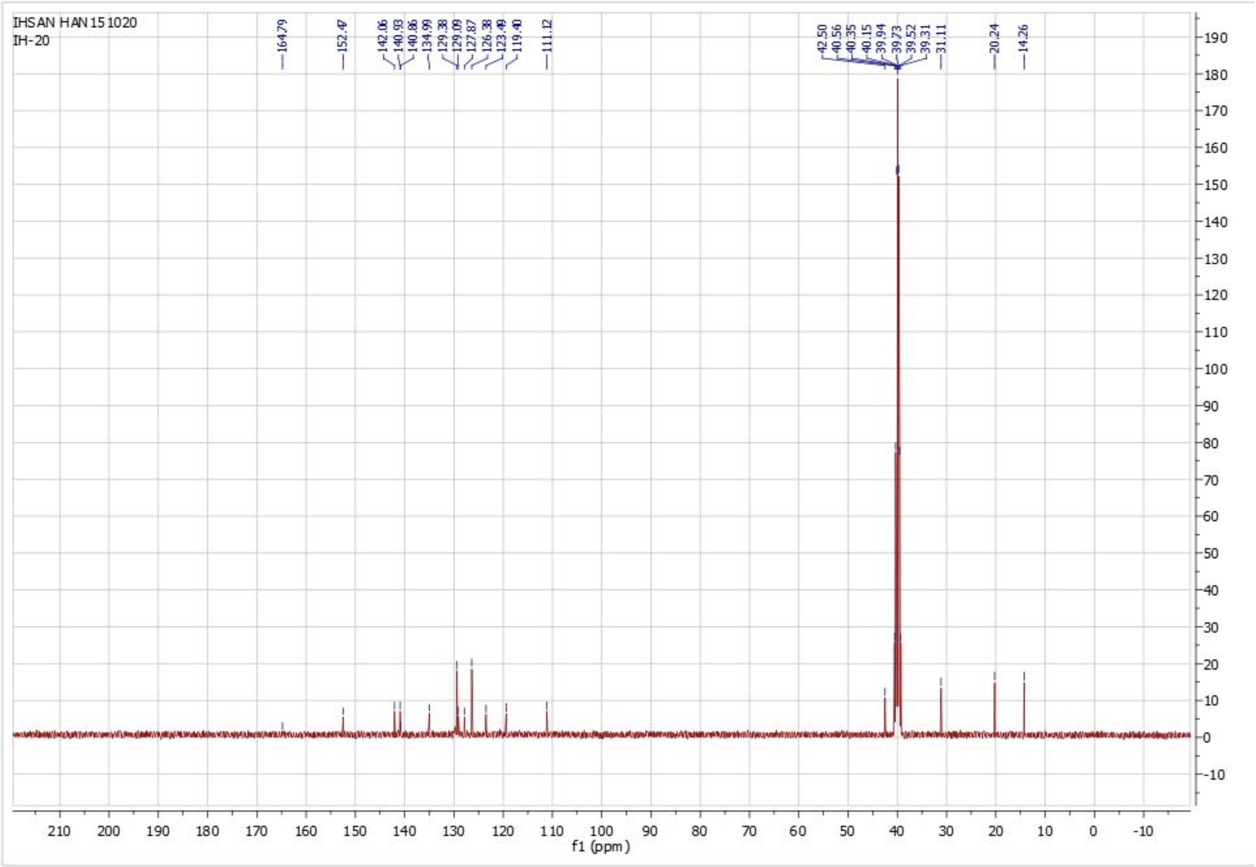

Figure S79. ^{13}C-NMR Spectra of compound **2t**

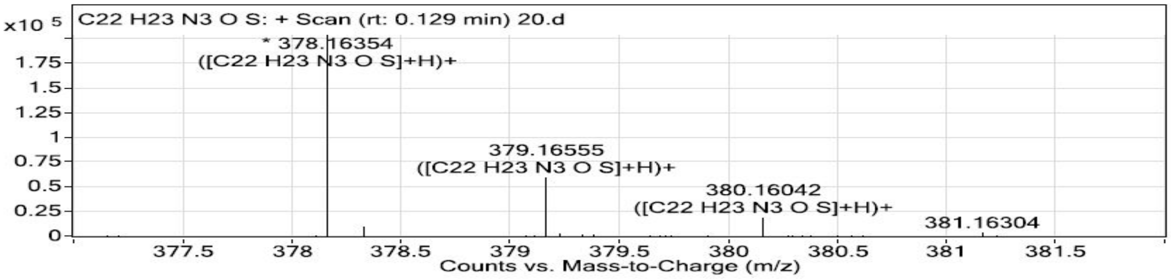

Figure S80. HR-Mass spectra of compound **2t**
